# Bioinformatic Identification and Analysis of Hydroxyproline-Rich Glycoproteins in *Populus trichocarpa*

**DOI:** 10.1186/s12870-016-0912-3

**Published:** 2016-10-21

**Authors:** Allan M. Showalter, Brian D. Keppler, Xiao Liu, Jens Lichtenberg, Lonnie R. Welch

**Affiliations:** 1 Department of Environmental and Plant Biology, Molecular and Cellular Biology Program, Ohio University, 504 Porter Hall, Athens, OH 45701-2979 USA; 2 Russ College of Engineering and Technology, Center for Intelligent, Distributed and Dependable Systems, Ohio University, Athens, OH 45701-2979 USA

**Keywords:** Arabinogalactan-protein, Bioinformatics, Extensin, Hydroxyproline-rich glycoprotein, Plant cell wall, Poplar, *Populus trichocarpa*, Proline-rich protein

## Abstract

**Background:**

Hydroxyproline-rich glycoproteins (HRGPs) constitute a plant cell wall protein superfamily that functions in diverse aspects of growth and development. This superfamily contains three members: the highly glycosylated arabinogalactan-proteins (AGPs), the moderately glycosylated extensins (EXTs), and the lightly glycosylated proline-rich proteins (PRPs). Chimeric and hybrid HRGPs, however, also exist. A bioinformatics approach is employed here to identify and classify AGPs, EXTs, PRPs, chimeric HRGPs, and hybrid HRGPs from the proteins predicted by the completed genome sequence of poplar (*Populus trichocarpa*). This bioinformatics approach is based on searching for biased amino acid compositions and for particular protein motifs associated with known HRGPs with a newly revised and improved BIO OHIO 2.0 program. Proteins detected by the program are subsequently analyzed to identify the following: 1) repeating amino acid sequences, 2) signal peptide sequences, 3) glycosylphosphatidylinositol lipid anchor addition sequences, and 4) similar HRGPs using the Basic Local Alignment Search Tool (BLAST).

**Results:**

The program was used to identify and classify 271 HRGPs from poplar including 162 AGPs, 60 EXTs, and 49 PRPs, which are each divided into various classes. This is in contrast to a previous analysis of the Arabidopsis proteome which identified 162 HRGPs consisting of 85 AGPs, 59 EXTs, and 18 PRPs. Poplar was observed to have fewer classical EXTs, to have more fasciclin-like AGPs, plastocyanin AGPs and AG peptides, and to contain a novel class of PRPs referred to as the proline-rich peptides.

**Conclusions:**

The newly revised and improved BIO OHIO 2.0 bioinformatics program was used to identify and classify the inventory of HRGPs in poplar in order to facilitate and guide basic and applied research on plant cell walls. The newly identified poplar HRGPs can now be examined to determine their respective structural and functional roles, including their possible applications in the areas plant biofuel and natural products for medicinal or industrial uses. Additionally, other plants whose genomes are sequenced can now be examined in a similar way using this bioinformatics program which will provide insight to the evolution of the HRGP family in the plant kingdom.

**Electronic supplementary material:**

The online version of this article (doi:10.1186/s12870-016-0912-3) contains supplementary material, which is available to authorized users.

## Background

The hydroxyproline-rich glycoproteins (HRGPs) constitute a diverse superfamily of glycoproteins found throughout the plant kingdom [1–6]. Based on their patterns of proline hydroxylation and subsequent glycosylation, HRGPs are separated into three families: arabinogalactan-proteins (AGPs), extensins (EXTs), and proline-rich proteins (PRPs). These differences in proline hydroxylation and glycosylation are ultimately determined by the primary amino acid sequence, particularly with respect to the location and distribution of proline residues. Specifically, AGPs typically contain non-contiguous proline residues (e.g., APAPAP) which are hydroxylated and glycosylated with arabinogalactan (AG) polysaccharides [7–9]. In contrast, EXTs typically contain contiguous prolines (e.g., SPPPP) that are hydroxylated and subsequently glycosylated with arabinose oligosaccharides [2, 10]. The PRPs typically contain stretches of contiguous proline residues which are shorter than those found in EXTs; these proline residues may be hydroxylated and subsequently glycosylated with arabinose oligosaccharides. Thus, AGPs are extensively glycosylated, EXTs are moderately glycosylated, and PRPs are lightly glycosylated, if at all. In addition, most HRGPs have an N-terminal signal peptide that results in their insertion into the endomembrane system and delivery to the plasma membrane/cell wall. Certain families of HRGPs, particularly the AGPs, are also modified with a C-terminal glycosylphosphatidylinositol (GPI) membrane anchor, which tethers the protein to the outer leaflet of plasma membrane and allows the rest of the glycoprotein to extend toward the cell wall in the periplasm [11–13]. These characteristic amino acid sequences and sequence features allow for the effective identification and classification of HRGPs from proteomic databases by bioinformatic approaches involving biased amino acid composition searches and/or HRGP amino acid motif searches [14–17]. In addition, Newman and Cooper [18] utilized another bioinformatic approach involving searching for proline-rich tandem repeats to identify numerous HRGPs as well as other proteins in a variety of plant species.

The AGP family can be divided into the classical AGPs, which include a subset of lysine-rich classical AGPs, and the AG peptides. In addition, chimeric AGPs exist, most notably the fasciclin-like AGPs (FLAs) and the plastocyanin AGPs (PAGs), but also other proteins which have AGP-like regions along with non-HRGP sequences. Classical AGPs are identified using a search for proteins whose amino acid composition consists of at least 50 % proline (P), alanine (A), serine (S), and theronine (T), or more simply, 50 % PAST [14, 16]. Similarly, AG peptides are identified with a search of 35 % PAST, but are size limited to be between 50 and 90 amino acids in length. EXTs contain characteristic SPPP and SPPPP repeats. As such, EXTs are identified by searching for proteins that contain at least two SPPP repeats. Finally, PRPs are identified by searching for proteins that contain at least 45 % PVKCYT or contain two or more repeated motifs (PPVX[KT] or KKPCPP). Similar to AGPs, chimeric versions of EXTs and PRPs also exist. Each HRGP identified here in this poplar study can then be subjected to BLAST searches against both the Arabidopsis and poplar databases for several purposes: 1) to ensure that the protein identified is similar in sequence to some known HRGPs in Arabidopsis, 2) to identify if the protein is similar to other proteins in poplar which were identified as HRGPs by using the BIO OHIO 2.O program, and 3) to identify similar proteins that may be HRGPs, but which do not meet the search criteria.

Although the numbers and types of HRGPs in Arabidopsis are well established [14, 16], much less is known in other plant species. As more plant genome sequencing projects are completed, comprehensive identification and analysis of HRGPs in these species can be completed. This knowledge can be used to facilitate and guide basic and applied research on these cell wall proteins, potentially with respect to plant biofuel research that utilizes cell wall components for energy production. In fact, a paper was recently published linking poplar EXTs to recalcitrance [19]. Moreover, comparisons can be made with what is already known in Arabidopsis, which will potentially provide further insight into the roles that these particular classes of HRGPs play in the plant as well as their evolution. A comprehensive inventory of HRGPs in poplar, or trees in general, is lacking, although a search for proline-rich tandem repeat proteins in poplar recently identified several HRGP sequences [18]. Additionally, 15 fasciclin-like AGPs (FLAs) were identified in *Populus tremula × P. alba*, a hybrid related to *Populus trichocarpa,* and found to be highly expressed in tension wood [20].

**Fig. 1:**
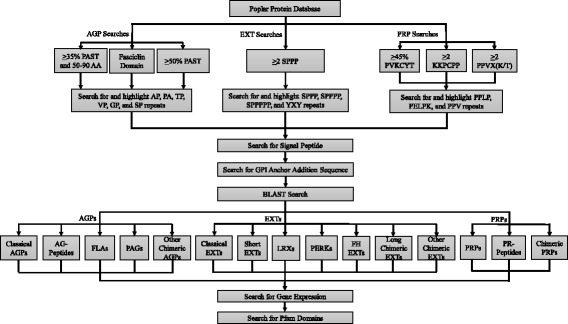
Workflow diagram for the identification, classification, and analysis of HRGPs (AGPs, EXTs, and PRPs) in poplar using a newly revised and improved BIO OHIO 2.0. Classical AGPs were characterized as containing greater than 50 % PAST. AG peptides were characterized to be 50 to 90 amino acids in length and containing greater than 35 % PAST. FLAs were characterized as having a fasciclin domain. Chimeric AGPs were characterized as containing greater than 50 % PAST coupled with one or more domain(s) not known in HRGPs. All AGPs feature the presence of AP, PA, TP, VP, GP, and SP repeats distributed throughout the protein. EXTs were defined as containing two or more SPPP repeats coupled with the distribution of such repeats throughout the protein; chimeric extensins, including LRXs, PERKs, FH EXTs, long chimeric EXTs (>2000 aa), and other chimeric EXTs, were similarly identified but were distinguished from the classical EXTs by the localized distribution of such repeats in the protein and the presence of non-HRGP sequences/domains, many of which were identified by the Pfam analysis; and short extensins were defined to be less than 200 amino acids in length coupled with the EXT definition. PRPs were identified to contain greater than 45 % PVKCYT or two or more KKPCPP or PVX(K/T) repeats coupled with the distribution of such repeats and/or PPV throughout the protein. Chimeric PRPs were similarly identified but were distinguished from PRPs by the localized distribution of such repeats in the protein. Other integrated functional modules include searching for the presence of a signal peptide to provide added support for the identification of an HRGP; the presence of a GPI anchor addition sequence for added support for the identification of AGPs, and BLAST searches to provide some support to the classification. Tissue/organ-specific expression data were also obtained for identified HRGPs to guide for future research

Here, the completed genome sequence, or more precisely the encoded proteome, of *Populus trichocarpa* was utilized to successfully conduct a comprehensive bioinformatics based approach for the identification of HRGPs in this species (Fig. 1). This approach utilizes a newly revised and improved BIO OHIO 2.0 program. Since Arabidopsis and poplar are both dicots, they are expected to have a similar inventory of HRGPs, as opposed to the monocots, which may prove to be considerably different. Nevertheless, Arabidopsis and poplar are morphologically different from one another with Arabidopsis being a small annual herbaceous plant and with poplar being a large woody deciduous tree. Distinct differences were reflected in their inventories of HRGPs, which can now be used to guide further research on the functional roles, commercial applications, and evolution of these ubiquitous and highly modified plant glycoproteins.

## Methods

### Identification of AGPs, EXTs, and PRPs using BIO OHIO 2.0

The *Populus trichocarpa* protein database (Ptrichocarpa_210_v3.0.protein.fa.gz) was downloaded from the Phytozome v11.0 website (www.phytozome.org) [21]. The protein database was searched for AGPs, EXTs, and PRPs using the newly revised and improved BIO OHIO 2.0 software [16, 22]. Compared to the previous version, this new version integrated more functional modules that include searching for the presence of a signal peptide at the SignalP server (www.cbs.dtu.dk/services/SignalP/) [23], searching for the presence of GPI anchor addition sequences using the big-PI plant predictor (mendel.imp.ac.at/gpi/plant_server.html) [24], as well as an automated BLAST search against Arabidopsis proteome. In cases where no signal peptide was identified using the default parameters for a sequence, the sensitive mode was then used which lowered the D-cutoff values to 0.34 [23]. These improvements make the program an ideal bioinformatic tool to study cell wall proteins/glycoproteins within any sequenced plant species. The program is freely available upon request. Briefly, classical AGPs were identified as proteins of any length that consisted of 50 % or greater of the amino acids P, A, S, and T (PAST). AG peptides were identified as proteins of 50–90 amino acids in length consisting of 35 % or greater PAST. FLAs were designated as proteins containing the following consensus motif:

$$ \left[\mathrm{MALIT}\right]\mathrm{T}\left[\mathrm{VILS}\right]\left[\mathrm{FLCM}\right]\left[\mathrm{CAVT}\right]\left[\mathrm{PVLIS}\right]\left[\mathrm{GSTKRNDPEIV}\right] + \left[\mathrm{D}\mathrm{N}\mathrm{S}\right]\left[\mathrm{D}\mathrm{S}\mathrm{ENAGE}\right] + \left[\mathrm{ASQM}\right] $$

EXTs were identified by searching with a regular expression for the occurrence of two or more SPPP repeats in the protein. Hits were examined for the location and distribution of SP3 and SP4 repeats as well as for the occurrence of other repeating sequences, including YXY. PRPs were identified by searching for a biased amino acid composition of greater than 45 % PVKCYT or for sequences containing two or more repeated motifs (PPVX[KT] or KKPCPP) [25].

### BLAST Analysis against Arabidopsis and poplar proteomes

All proteins identified by the BIO OHIO 2.0 searches were subjected to protein-protein BLAST (blastp) analysis. BLAST analysis against Arabidopsis HRGPs was conducted as an integrated module within BIO OHIO 2.0. BLAST analysis against the poplar database (Ptrichocarpa_210_v3.0.protein.fa) was conducted using NCBI BLAST+ (2.2.30) downloaded from the NCBI website. BLAST searches were conducted with the “filter query” option both on and off.

### Pfam database and poplar HRGP Gene Expression Database

All proteins identified in this study were subjected to a sequence search using Pfam database 30.0 (http://pfam.xfam.org/) to identify Pfam matches within the protein sequences [26], and the Poplar eFP Browser (http://bar.utoronto.ca/efppop/cgi-bin/efpWeb.cgi) for organ/tissue-specific expression data [27]. Specifically, protein sequences of poplar v3.0 were entered into the Pfam database, while poplar v2.0 identifiers were entered into the Poplar eFP Browser since the eFP browser currently does not recognize poplar v3.0 identifiers.

## Results

### Arabinogalactan-proteins (AGPs)

Among the 73,013 proteins in the poplar database, 86 proteins were found to have at least 50 % PAST, while 194 peptides have at least 35 % PAST, and are between 50 and 90 amino acids in length (Table 1). Several chimeric AGPs were identified in the 50 % PAST search, but the FLAs in particular required a unique test as they typically do not meet the 50 % PAST threshold. Previously in Arabidopsis, a consensus sequence for the fasciclin H1 domain was utilized to search for these proteins, and this consensus sequence was again utilized here [16]. A total of 43 proteins were found to contain this sequence.

**Table 1:** AGPs, EXTs, and PRPs identified from the *Populus trichocarpa* protein database based on biased amino acid compositions, size, and repeat units

Search Criteria	Total	Classical AGPs	Lys-Rich AGPs	AG Peptides	FLAs	PAGs	Other Chimeric AGPs	EXTs	Short EXTs	LRXs	PERKs	FH EXTs	Other Chimeric EXTs	PRPs	PR Peptides	Chimeric PRPs	Others
≥50 % PAST	86	10	5	0	1	5	0	7	4	0	0	0	0	1	16	0	37
≥35 % PAST and 50-90 AA	194	0	0	31	0	0	0	0	0	0	0	0	0	0	0	0	163
Fasciclin domain	43	0	0	0	24	0	0	0	0	0	0	0	0	0	0	0	19
≥2 SPPP	162	1	1	0	0	2	0	8	21	10	12	5	3	0	0	0	99
≥2 KKPCPP	0	0	0	0	0	0	0	0	0	0	0	0	0	0	0	0	0
≥2 PPV.[KT]	29	0	0	0	0	0	0	0	0	0	0	0	0	4	0	0	25
≥45 % PVKCYT	240	4	5	0	0	0	1	8	8	0	0	0	0	10	10	0	194

**Fig. 2:**
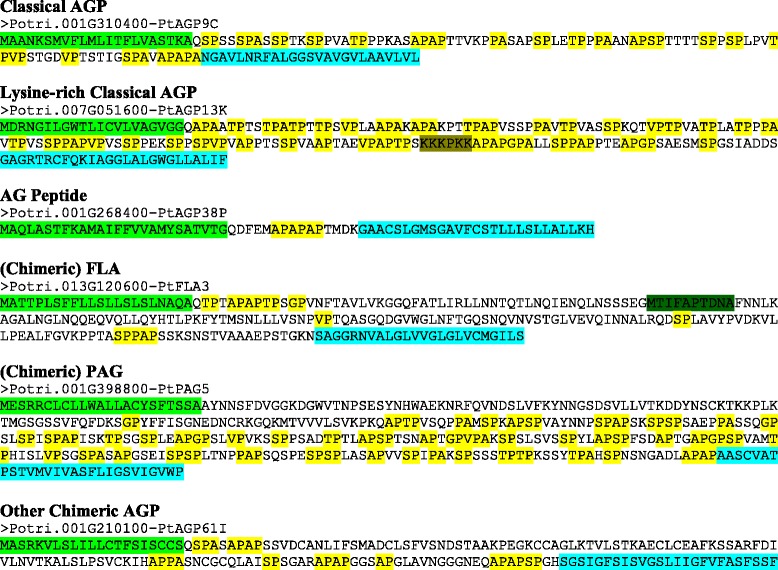
Protein sequences encoded by the representative AGP gene classes in *Populus trichocarpa*. The colored sequences at the N and C terminus indicate predicted signal peptides (green) and GPI anchor addition sequences (light blue) if present. AP, PA, SP, TP, VP, and GP repeats (yellow), lysine-rich regions (olive) and core fasciclin motif (dark green) are also indicated

In addition to meeting one of the search criteria, several other factors were considered in determining if the proteins were classified as HRGPs. All proteins were examined for signal peptides and for GPI membrane anchor addition sequences, as these are known to occur in AGPs. In addition, sequences were examined for certain dipeptide repeats which are characteristic of AGPs, including AP, PA, SP, TP, VP, and GP [3, 28]. The presence of these repeats was used to determine if a protein identified by the search was classified as an AGP. The various searches for AGPs combined with BLAST searches identified a total of 162 poplar proteins that were determined to be AGPs (Table 2). In total, 27 classical AGPs (which include six lysine-rich AGPs) and 35 AG peptides were identified. In terms of chimeric AGPs, FLAs were particularly abundant in poplar with 50 being identified. Using the consensus sequence that identifies all 21 of the Arabidopsis FLAs, a total of 24 FLAs were identified in poplar. However, because a single amino acid change in the consensus sequence would result in a particular FLA not being identified, the additional 26 FLAs were identified with BLAST searches. Another particularly common class of chimeric AGPs identified in Arabidopsis was the plastocyanin AGPs, or PAGs. Only five PAGs were identified with the 50 % PAST search, but 34 others were identified that fall below the 50 % PAST threshold with BLAST searches. Finally, 11 other chimeric AGPs were also identified. Representative AGP sequences from each class are shown in Fig. 2, while sequences from all 162 AGPs identified are available in Additional file 1: Figure S1.

**Table 2:** Identification and analysis of AGP genes in *Populus trichocarpa*

Locus Identifier 3.0 (ID 2.0) ^a^	Name	Class	AP/PA/SP/TP/GP/VP Repeats	% PAST	Amino Acids	Pfam^b^	SP^c^	GPI	Organ/tissue-specific Expression^d^	Arabidopsis HRGP BLAST Hits	Poplar HRGP BLAST Hits^e^
Potri.017G050200	PtAGP1C	Classical	3/3/12/2/1/1	66 %	137		Y	Y		AtAGP1C, AtAGP17K, AtAGP18K, AtAGP7C	PtAGP2C, PtAGP7C, PtAGP9C, PtAGP5C, Potri.005G077100
Potri.017G050300 (POPTR_0017s07700)	PtAGP2C	Classical	5/5/9/2/1/1	64 %	133		Y	Y	Female catkins	AtAGP1C, AtAGP10C, AtAGP3C, AtPAG11	PtAGP9C, PtAGP1C, Potri.004G161700, Potri.001G376400, Potri.009G009600
Potri.005G161100 (POPTR_0005s17440)	PtAGP3C	Classical	11/9/8/5/0/2	59 %	161		Y	N	Roots	AtAGP10C, AtAGP3C, AtAGP5C, AtAGP18K, AtPERK13	Potri.013G119700, Potri.009G124200, Potri.004G162500, Potri.001G376400, Potri.013G112500
Potri.014G135100 (POPTR_0014s12960)	PtAGP4C	Classical	4/4/6/1/2/0	54 %	140		Y	Y	Dark etiolated seedlings, light-grown seedling, young leaf	AtAGP26C, AtAGP27C, AtAGP25C	PtAGP47C, PtAGP48C, PtAGP49K, Potri.013G119700, Potri.004G196400
Potri.001G339700 (POPTR_0001s35940)	PtAGP5C	Classical	9/8/4/3/4/0	59 %	144		Y	Y	Male catkins	AtAGP6C, AtAGP11C, AtAGP17K	PtAGP50C, Potri.003G031800, PtAGP51C, PtAGP52C, Potri.003G143000
Potri.001G259700	PtAGP6C	Classical	1/3/20/3/0/1	57 %	197		Y	N	None		PtAGP43P, PtPtEXT7, PtPtEXT4
Potri.001G310300 (POPTR_0001s31780)	PtAGP7C	Classical	6/7/8/5/0/2	63 %	126		Y	Y	Young leaf	AtAGP6C	PtAGP1C, PtAGP9C, Potri.002G256200, Potri.002G235500, Potri.005G049100
Potri.001G367600	PtAGP8C	Classical	7/8/29/4/1/1	68 %	265		Y	Y	None	Potri.004G145800	
Potri.001G310400 (POPTR_0001s31790)	PtAGP9C	Classical	6/7/9/3/0/2	62 %	137		Y	Y	Young leaf	AtAGP18K, AtAGP1C, AtPEX4, AtAGP10C	PtAGP2C, Potri.009G085400, Potri.013G119700, PtAGP7C, Potri.005G043900
Potri.017G047500 (POPTR_0017s07480)	PtAGP10C	Classical	0/2/4/5/1/3	50 %	207		Y	Y	Female catkins	None	Potri.011G046900, Potri.010G094700, PtPRP23, Potri.004G038300, PtPRP28
*Potri.002G207500* (POPTR_0020s00250)	PtAGP47C	Classical	4/4/6/1/2/0	49 %	141		Y	N	Xylem	AtAGP26C, AtAGP27C	PtAGP4C, PtAGP48C, PtAGP49K, Potri.013G119700, Potri.003G164300
*Potri.010G031700 (POPTR_0010s03290)*	PtAGP48C	Classical	2/2/9/2/1/2	44 %	169		Y*	N	Xylem	AtAGP26C, AtAGP25C, AtAGP27C	PtAGP49K, PtAGP4C, PtAGP47C, Potri.008G153000, Potri.008G147100
*Potri.008G182400 (POPTR_0008s18270)*	PtAGP50C	Classical	3/2/1/0/3/1	47 %	101		Y	Y	Male catkins	AtAGP50C, AtAGP6C, AtAGP5C	PtAGP52C, PtAGP51C, PtAGP5C, Potri.013G011700, Potri.018G128000
*Potri.015G093700 (POPTR_0015s10580)*	PtAGP51C	Classical	6/3/0/0/2/1	49 %	115		Y	Y	Male catkins	AtAGP50C, AtAGP6C, AtAGP15P	PtAGP52C, PtAGP50C, PtAGP5C, Potri.014G159300, Potri.009G065300
*Potri.012G095900 (POPTR_0012s09790)*	PtAGP52C	Classical	6/5/0/0/2/1	49 %	115		Y	Y	Male catkins	AtAGP50C, AtAGP6C, AtAGP3C	PtAGP51C, PtAGP50C, PtAGP5C, Potri.014G159300, Potri.019G095800
*Potri.005G169000*	PtAGP64C	Classical	10/9/4/1/0/3	48 %	216	PF14368.4	Y	N		AtAGP29I	PtAGP60I, PtAGP57I, PtAGP58I, Potri.001G210100, PtAGP69C
*Potri.008G155200 (POPTR_0008s15500)*	PtAGP65C	Classical	4/4/3/4/0/7	45 %	219	PF14368.4	Y*	Y	Xylem, male catkins, female catkins	AtAGP29I	Potri.010G085200, PtAGP66C, PtAGP67C, PtAGP68C, PtAGP69C
*Potri.005G212000 (POPTR_0005s23360)*	PtAGP66C	Classical	4/4/5/4/2/2	45 %	207	PF14368.4	Y	Y	Roots	AtAGP29I	PtAGP67C, Potri.010G085200, PtAGP65C, PtAGP69C, PtAGP68C
*Potri.002G050200 (POPTR_0002s05110)*	PtAGP67C	Classical	4/5/5/4/2/2	46 %	205	PF14368.4	Y	N		AtAGP29I	PtAGP66C, Potri.010G085200, PtAGP65C, PtAGP68C, PtAGP69C
*Potri.010G085400 (POPTR_0010s09550)*	PtAGP68C	Classical	0/2/4/4/0/1	44 %	170	PF14368.4	Y	Y	Male catkins	AtAGP29I	PtAGP69C, Potri.005G211800, Potri.002G050500, Potri.002G050300, Potri.005G211900
*Potri.008G155100* *(POPTR_0008s15490)*	PtAGP69C	Classical	1/2/5/2/0/1	44 %	170	PF14368.4	Y	Y	Male catkins	AtAGP29I	PtAGP68C, Potri.005G211800, Potri.002G050500, Potri.010G085300, Potri.002G050300
Potri.009G092300 (POPTR_0009s09530)	PtAGP11K	Lysine-rich	11/19/8/11/1/2	69 %	196		Y	Y	Xylem	AtAGP17K, AtAGP18K, AtPRP1	PtAGP14K, Potri.004G181200, Potri.001G310900, PtAGP71I
Potri.010G132500 (POPTR_0010s14250)	PtAGP12K	Lysine-rich	18/24/10/12/0/4	65 %	241		Y	N	Xylem	AtAGP19K	PtAGP15K, Potri.013G003500, Potri.007G013600
Potri.007G051600 (POPTR_0007s10230)	PtAGP13K	Lysine-rich	12/12/9/11/2/5	60 %	204		Y	Y	Dark etiolated seedlings, young leaf	AtAGP17K, AtAGP18K	PtAGP14K, Potri.013G003500, PtAGP72I, Potri.018G122900
Potri.005G144900 (POPTR_0005s18840)	PtAGP14K	Lysine-rich	11/12/9/10/3/4	62 %	208		Y	Y	Female catkins	AtAGP18K, AtAGP17K, AtPRP1	PtAGP13K, Potri.002G008600, Potri.005G049100, Potri.006G234100
Potri.008G111000 (POPTR_0008s11040)	PtAGP15K	Lysine-rich	23/33/14/12/0/2	66 %	276		Y	Y	None	PtAGP12K, PtPtPAG5	
Potri.008G195700 (POPTR_0008s20030)	PtAGP49K	Lysine-rich	2/2/9/1/1/4	45 %	194		Y	N	Female catkins	AtAGP25C, AtAGP27C, AtAGP26C	PtAGP48C, PtAGP4C, PtAGP47C, Potri.008G147100, Potri.010G094700
Potri.009G063600 (POPTR_0006s05460)	PtAGP16P	AG peptide	2/2/1/0/0/0	48 %	60		Y	Y		AtAGP43P, AtAGP23P, AtAGP40P, AtAGP14P, AtAGP15P	PtAGP41P, PtAGP24P, Potri.016G052000, PtAGP29P, PtAGP28P
Potri.009G062700	PtAGP17P	AG peptide	2/2/0/0/0/0	36 %	68		Y	Y		AtAGP22P, AtAGP16P	PtAGP38P, PtAGP29P, PtAGP22P, PtAGP28P, PtAGP25P
Potri.009G063200	PtAGP18P	AG peptide	3/2/0/0/0/0	40 %	69		Y	Y		AtAGP43P	PtAGP39P, PtAGP19P, PtAGP29P, PtAGP38P, PtAGP53P
Potri.009G063000	PtAGP19P	AG peptide	3/2/0/0/0/0	41 %	70		Y	Y		None	PtAGP18P, PtAGP39P, PtAGP29P, PtAGP53P, PtAGP38P
Potri.013G057500 (POPTR_0013s05400)	PtAGP20P	AG peptide	2/2/1/0/0/1	41 %	60		Y	Y	Male catkins	AtAGP14P, AtAGP12P, AtAGP13P, AtAGP21P, AtAGP15P	PtAGP54P, PtAGP33P, PtAGP44P, PtAGP41P, PtAGP30P
Potri.003G136600 (POPTR_0003s13640)	PtAGP21P	AG peptide	3/2/0/0/0/0	39 %	69	PF06376.10	Y	Y	Female catkins, male catkins	AtAGP20P, AtAGP16P, AtAGP22P, AtAGP41P, AtAGP15P	PtAGP40P, PtAGP30P, PtAGP45P, PtAGP35P, PtAGP54P
Potri.006G056000 (POPTR_0831s00200)	PtAGP22P	AG peptide	3/2/0/0/0/0	36 %	68		Y	Y	Xylem	AtAGP40P, AtAGP43P	PtAGP53P, PtAGP28P, PtAGP29P, PtAGP27P, PtAGP25P
Potri.006G055700 (POPTR_0006s05460)	PtAGP23P	AG peptide	4/3/0/0/0/0	42 %	66		Y	Y	male catkins, dark etiolated seedlings	AtAGP16P, AtAGP43P	PtAGP29P, PtAGP27P, PtAGP22P, PtAGP25P, PtAGP28P
Potri.006G056200 (POPTR_0006s05490)	PtAGP24P	AG peptide	2/1/1/0/0/0	47 %	61		Y	Y	Male catkins	AtAGP43P, AtAGP23P, AtAGP40P, AtAGP13P, AtAGP14P	Potri.016G052000, PtAGP16P, PtAGP41P, PtAGP29P, PtAGP23P
Potri.006G055900	PtAGP25P	AG peptide	3/2/0/0/0/0	37 %	67		Y	Y		AtAGP43P, AtPAG2	PtAGP27P, PtAGP28P, PtAGP22P, PtAGP29P, PtAGP53P
Potri.006G055500 (POPTR_0006s05440)	PtAGP26P	AG peptide	4/3/1/0/0/0	39 %	69		Y	Y	Dark etiolated seedlings	AtAGP12P, AtAGP43P, AtAGP15P	PtAGP23P, PtAGP29P, PtAGP28P, PtAGP22P, PtAGP27P
Potri.006G055800	PtAGP27P	AG peptide	3/2/0/0/0/0	37 %	67		Y	Y		AtAGP43P, AtPAG2	PtAGP25P, PtAGP28P, PtAGP22P, PtAGP29P, PtAGP53P
Potri.016G052400 (POPTR_0016s05280)	PtAGP28P	AG peptide	3/2/0/0/0/0	37 %	67		Y	Y	Dark etiolated seedlings	AtAGP40P, AtAGP15P	PtAGP27P, PtAGP22P, PtAGP25P, PtAGP53P, PtAGP29P
Potri.016G052200 (POPTR_0016s05270)	PtAGP29P	AG peptide	3/2/1/0/0/1	38 %	67		Y	Y	Male catkins	AtAGP40P, AtAGP28I AtAGP43P, AtAGP12P	PtAGP22P, PtAGP27P, PtAGP25P, PtAGP28P, PtAGP53P
Potri.015G022600 (POPTR_0015s06130)	PtAGP30P	AG peptide	2/1/1/0/0/0	37 %	64	PF06376.10	Y	Y		AtAGP20P, AtAGP22P, AtAGP16P, AtAGP41P, AtAGP15P	PtAGP45P, PtAGP35P, PtAGP40P, PtAGP21P, Potri.001G070600
Potri.015G139200	PtAGP31P	AG peptide	2/0/0/1/0/0	35 %	57		Y	N		None	Potri.015G139100, Potri.012G137400, Potri.006G150100, Potri.008G094200, Potri.007G131100
Potri.002G226300 (POPTR_0002s21530)	PtAGP32P	AG peptide	1/1/4/0/1/1	37 %	74		Y	N		None	PtAGP34P, Potri.012G138200, Potri.001G274200, Potri.002G121800, Potri.015G140000
Potri.019G035500 (POPTR_0019s05110)	PtAGP33P	AG peptide	2/2/1/0/0/1	44 %	59		Y	Y		AtAGP14P, AtAGP12P, AtAGP13P, AtAGP21P, AtAGP22P	PtAGP20P, PtAGP54P, PtAGP44P, PtAGP41P, PtAGP30P
Potri.014G156600 (POPTR_0014s15480)	PtAGP34P	AG peptide	1/0/2/1/0/1	37 %	74		Y	N		None	PtAGP32P, Potri.001G274200, Potri.012G138200, Potri.015G140000, Potri.010G111200
Potri.014G094800 (POPTR_0014s09050)	PtAGP35P	AG peptide	3/3/2/0/0/0	42 %	76	PF06376.10	Y	N	Male catkins	AtAGP20P, AtAGP16P, AtAGP22P, AtAGP41P, AtAGP15P	PtAGP30P, PtAGP45P, PtAGP40P, PtAGP21P, PtAGP17P
Potri.T142100	PtAGP36P	AG peptide	1/2/2/1/0/0	36 %	90		Y	N		None	Potri.004G234800, Potri.014G034500, Potri.005G136800, Potri.007G041500, Potri.007G041400
Potri.001G387800 (POPTR_0001s39620)	PtAGP37P	AG peptide	1/0/3/0/0/0	37 %	78		Y	N	Female catkins, male catkins, young leaf	None	Potri.004G061300, Potri.011G070500, Potri.003G125800, Potri.008G019500, Potri.002G195300
Potri.001G268400 (POPTR_0001s27530)	PtAGP38P	AG peptide	3/2/0/0/0/0	39 %	68		Y	Y		AtAGP22P, AtPAG1	PtAGP17P, PtAGP29P, PtAGP22P, PtAGP28P, PtAGP27P
Potri.001G268500 (POPTR_0001s27540)	PtAGP39P	AG peptide	3/3/0/0/0/0	40 %	69		Y	Y		AtAGP15P, AtAGP14P, AtAGP28I AtAGP13P, AtPAG1	PtAGP18P, PtAGP19P, PtAGP29P, PtAGP53P, PtAGP38P
Potri.001G094700 (POPTR_0001s10310)	PtAGP40P	AG peptide	3/2/0/0/0/0	42 %	69	PF06376.10	Y	Y		AtAGP20P, AtAGP16P, AtAGP22P, AtAGP41P, AtAGP12P	PtAGP21P, PtAGP30P, PtAGP45P, PtAGP35P, Potri.016G086300
Potri.001G268800	PtAGP41P	AG peptide	2/1/1/0/0/0	46 %	60		Y	Y		AtAGP43P, AtAGP23P, AtAGP40P, AtAGP12P, AtAGP15P	PtAGP16P, PtAGP24P, Potri.016G052000, PtAGP29P, PtAGP28P
Potri.001G268900 (POPTR_0001s27570)	PtAGP42P	AG peptide	1/1/0/0/0/0	36 %	66		Y	Y		None	PtAGP29P, PtAGP56P, Potri.010G100200, Potri.011G126900, PtAGP23P
Potri.001G259500	PtAGP43P	AG peptide	0/0/3/1/0/0	37 %	67		Y	N		None	PtAGP6C, PtEXT7, PtEXT4, Potri.018G145800, Potri.007G096600
Potri.001G004100 (POPTR_0001s04130)	PtAGP44P	AG peptide	2/1/1/0/0/1	40 %	59		Y	Y		AtAGP14P, AtAGP12P, AtAGP13P, AtAGP21P, AtAGP15P	PtAGP54P, PtAGP20P, PtAGP33P, PtAGP41P, PtAGP60I
Potri.012G032000 (POPTR_0012s01350)	PtAGP45P	AG peptide	2/1/1/0/0/0	39 %	64	PF06376.10	Y	Y	Male catkins	AtAGP20P, AtAGP16P, AtAGP22P, AtAGP41P, AtAGP15P	PtAGP30P, PtAGP35P, PtAGP40P, PtAGP21P, PtAGP54P
Potri.012G144100	PtAGP46P	AG peptide	1/1/1/2/0/1	41 %	89		Y	N		None	Potri.002G258000, Potri.007G124600, Potri.003G086400, Potri.001G148100, Potri.013G051400
Potri.016G052300	PtAGP53P	AG peptide	3/2/1/0/0/0	32 %	110		Y*	Y		AtAGP15P, AtAGP40P, AtPAG11, AtAGP43P, AtPERK3	PtAGP22P, PtAGP28P, PtAGP27P, PtAGP25P, PtAGP29P
*Potri.003G220900 (POPTR_0003s21020)*	PtAGP54P	AG peptide	3/1/1/1/0/1	37 %	139		Y*	Y		AtAGP14P, AtAGP12P, AtAGP13P, AtAGP21P, AtAGP22P	PtAGP44P, PtAGP20P, PtAGP33P, PtAGP41P, Potri.004G067400
*Potri.006G056100 (POPTR_0006s05480)*	PtAGP55P	AG peptide	1/1/0/1/0/0	33 %	66		Y	N		None	PtAGP56P, PtAGP28P, PtAGP29P, PtAGP22P, PtAGP25P
*Potri.016G052100 (POPTR_0016s05260)*	PtAGP56P	AG peptide	1/1/0/1/0/0	31 %	66		Y	N	Xylem	None	PtAGP55P, PtAGP29P, PtAGP25P, PtAGP27P, PtAGP22P
Potri.010G244900 (POPTR_0010s25110)	PtFLA1	Chimeric	10/4/0/0/3/1	26 %	459	PF02469.20	Y	N		AtFLA17, AtFLA16, AtFLA18, AtFLA15, AtFLA12	PtFLA19, PtFLA6, PtFLA8, PtFLA41, Potri.012G006200
Potri.009G012200 (POPTR_0009s01740)	PtFLA2	Chimeric	8/7/3/2/2/0	39 %	254	PF02469.20	Y	N		AtFLA11, AtFLA12, AtFLA13, AtFLA9, AtFLA6	PtFLA34, PtFLA10, PtFLA23, PtFLA40, PtFLA48
Potri.013G120600 (POPTR_0013s12490)	PtFLA3	Chimeric	4/2/2/3/1/1	34 %	238	PF02469.20	Y	Y	Dark etiolated seedlings, roots, female catkins	AtFLA6, AtFLA9, AtFLA13, AtFLA11, AtFLA12	PtFLA15, PtFLA9, PtFLA7, PtFLA10, PtFLA23
Potri.013G152200 (POPTR_0013s14840)	PtFLA4	Chimeric	5/0/5/0/1/0	31 %	353	PF02469.20	N	N	Female catkins	AtFLA21, AtFLA19, AtFLA20, AtFLA15, AtFLA16	Potri.019G125200, PtFLA36, PtFLA42, PtFLA44, Potri.T118500
Potri.011G093500 (POPTR_0011s09590)	PtFLA5	Chimeric	7/4/2/2/1/2	32 %	408	PF02469.20	Y	Y		AtFLA1, AtFLA2, AtFLA8, AtFLA10, AtFLA14	PtFLA22, PtFLA16, PtFLA17, PtFLA21, PtFLA37
Potri.006G200300 (POPTR_0006s21460)	PtFLA6	Chimeric	8/2/1/0/3/1	27 %	466	PF02469.20	Y	N		AtFLA17, AtFLA18, AtFLA16, AtFLA15, AtFLA11	PtFLA8, PtFLA1, PtFLA19, PtFLA41, Potri.012G006200
Potri.006G129200 (POPTR_0006s13120)	PtFLA7	Chimeric	6/5/2/1/1/2	36 %	227	PF02469.20	Y	N		AtFLA11, AtFLA12, AtFLA6, AtFLA13, AtFLA9	PtFLA9, PtFLA10, PtFLA23, PtFLA32, PtFLA49
Potri.016G066500 (POPTR_0016s06680)	PtFLA8	Chimeric	7/2/2/1/3/1	27 %	466	PF02469.20	Y	N	Male catkins, and light etiolated seedlings, light grown seedling	AtFLA17, AtFLA18, AtFLA16, AtFLA15, AtFLA11	PtFLA6, PtFLA1, PtFLA19, PtFLA41, Potri.012G006200
Potri.016G088700 (POPTR_0016s09010)	PtFLA9	Chimeric	7/6/2/1/1/2	37 %	239	PF02469.20	Y	Y	Xylem	AtFLA11, AtFLA12, AtFLA6, AtFLA13, AtFLA9	PtFLA7, PtFLA10, PtFLA23, PtFLA32, PtFLA49
Potri.015G129400 (POPTR_0015s14570)	PtFLA10	Chimeric	5/5/3/2/1/1	37 %	240	PF02469.20	Y	Y	Xylem	AtFLA11, AtFLA12, AtFLA6, AtFLA13, AtFLA9	PtFLA23, PtFLA34, PtFLA2, PtFLA20, PtFLA28
Potri.T130300 (POPTR_0018s03790)	PtFLA11	Chimeric	8/3/3/1/2/2	40 %	271		Y	Y	Male catkins	AtFLA3, AtFLA5, AtFLA14, AtFLA8, AtFLA10	PtFLA25, PtFLA26, PtFLA21, PtFLA17, PtFLA16
Potri.002G223300 (POPTR_0002s22020)	PtFLA12	Chimeric	8/7/5/4/1/1	41 %	263	PF02469.20	Y	Y	Xylem	AtFLA7, AtFLA6, AtFLA11, AtFLA9, AtFLA12	PtFLA18, PtFLA3, PtFLA9, PtFLA7, PtFLA23
Potri.019G122600 (POPTR_0019s14350)	PtFLA13	Chimeric	7/5/1/0/0/2	39 %	215	PF02469.20	N	N		AtFLA12, AtFLA11, AtFLA13, AtFLA9, AtFLA6	PtFLA45, PtFLA35, PtFLA39, PtFLA29, PtFLA47
Potri.019G120800 (POPTR_0019s14320)	PtFLA14	Chimeric	10/10/2/1/0/1	43 %	214	PF02469.20	N	N		AtFLA12, AtFLA11, AtFLA9, AtFLA13, AtFLA6	PtFLA39, PtFLA28, 7PtFLA13, PtFLA45, PtFLA35
Potri.019G093300 (POPTR_0019s12310)	PtFLA15	Chimeric	6/5/3/0/1/1	34 %	245	PF02469.20	Y	Y	Dark etiolated seedlings	AtFLA6, AtFLA9, AtFLA13, AtFLA11, AtFLA12	PtFLA3, PtFLA9, PtFLA7, PtFLA10, PtFLA23
Potri.014G168100 (POPTR_0014s16610)	PtFLA16	Chimeric	9/1/0/0/1/0	30 %	397	PF02469.20	Y	Y	Roots	AtFLA2, AtFLA1, AtFLA8, AtFLA10, AtFLA4	PtFLA22, PtFLA5, PtFLA17, PtFLA21, PtFLA37
Potri.014G071700 (POPTR_0014s06740)	PtFLA17	Chimeric	13/7/7/4/1/3	42 %	421	PF02469.20	Y	Y	Xylem	AtFLA10, AtFLA8, AtFLA2, AtFLA1, AtFLA14	PtFLA16, PtFLA22, PtFLA5, PtFLA21, PtFLA25
Potri.014G162900 (POPTR_0014s16100)	PtFLA18	Chimeric	7/6/7/4/1/1	40 %	262	PF02469.20	Y	Y	Xylem	AtFLA7, AtFLA6, AtFLA9, AtFLA11, AtFLA12	PtFLA12, PtFLA3, PtFLA9, PtFLA7, PtFLA23
Potri.008G012400 (POPTR_0008s01310)	PtFLA19	Chimeric	11/4/1/0/3/1	27 %	463	PF02469.20	Y	N	Xylem	AtFLA17, AtFLA16, AtFLA18, AtFLA15, AtFLA12	PtFLA1, PtFLA6, PtFLA8, PtFLA41, Potri.012G006200
Potri.001G320800 (POPTR_0001s32800)	PtFLA20	Chimeric	7/6/3/1/1/1	37 %	243	PF02469.20	Y	Y	Xylem	AtFLA11, AtFLA12, AtFLA6, AtFLA13, AtFLA9	PtFLA10, PtFLA23, PtFLA39, PtFLA34, PtFLA13
Potri.001G037800 (POPTR_0001s07490)	PtFLA21	Chimeric	2/5/7/2/4/2	43 %	281	PF02469.20	Y	Y	Male catkins	AtFLA14, AtFLA8, AtFLA10, AtFLA3, AtFLA2	PtFLA26, PtFLA25, PtFLA11, PtFLA17, PtFLA5
Potri.001G367900 (POPTR_0001s37650)	PtFLA22	Chimeric	7/4/2/2/1/1	33 %	406	PF02469.20	Y	Y	Dark etiolated seedlings, young leaf	AtFLA1, AtFLA2, AtFLA8, AtFLA10, AtFLA14	PtFLA5, PtFLA16, PtFLA17, PtFLA21, PtFLA37
Potri.012G127900 (POPTR_0012s14510)	PtFLA23	Chimeric	5/3/2/2/2/1	35 %	240	PF02469.20	Y	Y	Xylem	AtFLA11, AtFLA12, AtFLA6, AtFLA9, AtFLA13	PtFLA10, PtFLA22, PtFLA34, PtFLA2, PtFLA20
Potri.001G440800 (POPTR_0001s43130)	PtFLA24	Chimeric	8/5/8/16/3/2	50 %	399		Y	Y	Male catkins	AtFLA20, AtFLA19, AtFLA21, AtFLA15, AtFLA17	Potri.T118500, PtFLA44, PtFLA36, Potri.019G125200, PtFLA19
*Potri.018G005100*	PtFLA25	Chimeric	8/3/3/1/2/2	40 %	271		Y	Y		AtFLA3, AtFLA5, AtFLA14, AtFLA8, AtFLA10	PtFLA11, PtFLA26, PtFLA21, PtFLA17, PtFLA16
*Potri.006G276200 (POPTR_0006s29110)*	PtFLA26	Chimeric	11/11/4/4/4/2	38 %	393		Y*	Y	Male catkins	AtFLA3, AtFLA14, AtFLA5, AtFLA8, AtFLA10	PtFLA11, PtFLA25, PtFLA21, PtFLA17, PtFLA16
*Potri.012G015000 (POPTR_0012s02210)*	PtFLA27	Chimeric	8/6/2/1/1/2	38 %	269	PF02469.20	Y	Y		AtFLA11, AtFLA12, AtFLA13, AtFLA6, AtFLA9	PtFLA48, PtFLA10, PtFLA23, PtFLA39, PtFLA28
*Potri.013G014200 (POPTR_0013s01570)*	PtFLA28	Chimeric	8/8/2/2/0/2	42 %	266	PF02469.20	Y	Y		AtFLA12, AtFLA11, AtFLA13, AtFLA9, AtFLA6	PtFLA39, PtFLA47, PtFLA50, PtFLA32, PtFLA49
*Potri.019G121200 (POPTR_0019s14420)*	PtFLA29	Chimeric	8/8/3/1/0/2	42 %	263	PF02469.20	Y	Y	Xylem	AtFLA11, AtFLA12, AtFLA13, AtFLA9, AtFLA6	PtFLA50, PtFLA32, PtFLA49, PtFLA28, PtFLA39
*Potri.006G174900 (POPTR_0006s18920)*	PtFLA30	Chimeric	1/4/5/3/0/2	38 %	426	PF02469.20	Y*	Y	Xylem	AtFLA4, AtFLA8, AtFLA10, AtFLA1, AtFLA2	PtFLA37, PtFLA17, PtFLA16, PtFLA5, PtFLA22
*Potri.008G127500 (POPTR_0008s12640)*	PtFLA31	Chimeric	1/0/3/1/0/1	29 %	292	PF02469.20	Y	N	Male catkins	AtFLA20, AtFLA19, AtFLA21, AtFLA10, AtFLA12	PtFLA36, PtFLA42, Potri.019G125200, PtFLA44, PtFLA4
*Potri.019G123200 (POPTR_0019s14430)*	PtFLA32	Chimeric	10/9/1/1/0/2	42 %	263	PF02469.20	Y	Y		AtFLA11, AtFLA12, AtFLA9, AtFLA13, AtFLA6,	PtFLA49, PtFLA50, PtFLA28, PtFLA39, PtFLA29
*Potri.019G120900 (POPTR_0019s14330)*	PtFLA33	Chimeric	8/8/3/1/0/2	42 %	227	PF02469.20	Y	Y	Xylem	AtFLA11, AtFLA12, AtFLA13, AtFLA9, AtFLA6	PtFLA43, PtFLA50, PtFLA32, PtFLA49, PtFLA29
*Potri.004G210600 (POPTR_0004s22030)*	PtFLA34	Chimeric	10/5/3/3/2/0	40 %	268	PF02469.20	Y	N	Xylem	AtFLA11, AtFLA12, AtFLA9, AtFLA13, AtFLA6	PtFLA2, PtFLA10, PtFLA23, PtFLA39, PtFLA40
*Potri.019G123000 (POPTR_0019s14410)*	PtFLA35	Chimeric	11/9/2/1/0/1	39 %	269	PF02469.20	Y	Y		AtFLA12, AtFLA11, AtFLA13, AtFLA9, AtFLA6	PtFLA45, PtFLA39, PtFLA28, PtFLA47, PtFLA13
*Potri.008G128200 (POPTR_0008s12720)*	PtFLA36	Chimeric	1/0/1/1/0/2	28 %	344	PF02469.20	Y	Y	Female catkins, male catkins	AtFLA20, AtFLA21, AtFLA19, AtFLA12, AtFLA6	PtFLA31, PtFLA42, PtFLA44, PtFLA4, Potri.T118500
*Potri.019G002300 (POPTR_0019s01620)*	PtFLA37	Chimeric	1/2/3/0/0/2	29 %	283		Y	N	Female catkins, young leaf	AtFLA19, AtFLA21, AtFLA20, AtFLA17, AtFLA16	Potri.001G306800, PtFLA4, Potri.T118500, PtFLA24, Potri.019G049600
*Potri.018G097000 (POPTR_0018s10600)*	PtFLA38	Chimeric	2/2/5/2/0/3	38 %	427	PF02469.20	Y*	N	Xylem	AtFLA4, AtFLA8, AtFLA10, AtFLA1, AtFLA2,	PtFLA30, PtFLA17, PtFLA16, PtFLA5, PtFLA22
*Potri.013G151300 (POPTR_0013s14760)*	PtFLA39	Chimeric	9/5/2/1/0/2	39 %	269	PF02469.20	Y	Y	Xylem	AtFLA12, AtFLA11, AtFLA13, AtFLA6, AtFLA9	PtFLA40, PtFLA28, PtFLA47, PtFLA45, PtFLA50
*Potri.013G151400 (POPTR_0013s14780)*	PtFLA40	Chimeric	9/9/2/1/0/2	40 %	269	PF02469.20	Y	Y	Xylem	AtFLA11, AtFLA12, AtFLA13, AtFLA9, AtFLA6	PtFLA39, PtFLA28, PtFLA47, PtFLA50, PtFLA32
*Potri.019G008400 (POPTR_0073s00210)*	PtFLA41	Chimeric	9/4/0/0/3/1	27 %	361	PF02469.20	N	N	Xylem	AtFLA17, AtFLA16, AtFLA18, AtFLA15, AtFLA7	PtFLA1, Potri.012G006200, PtFLA19, PtFLA6, PtFLA8
*Potri.017G111600 (POPTR_0017s14020)*	PtFLA42	Chimeric	5/2/4/2/0/2	30 %	352	PF02469.20	Y	N	Male catkins	AtFLA20, AtFLA21, AtFLA19, AtFLA10, AtFLA6	PtFLA36, PtFLA31, PtFLA44, PtFLA4, Potri.019G125200
*Potri.019G122800 (POPTR_0019s14390)*	PtFLA43	Chimeric	9/8/3/0/0/2	41 %	252	PF02469.20	Y	Y	Xylem	AtFLA11, AtFLA12, AtFLA9, AtFLA13, AtFLA6	PtFLA50, PtFLA32, PtFLA49, PtFLA29, PtFLA28
*Potri.005G079500 (POPTR_0005s08130)*	PtFLA44	Chimeric	3/3/5/2/1/6	33 %	442		Y	N	Male catkins	AtFLA21, AtFLA20, AtFLA19, AtFLA15	PtFLA36, PtFLA42, Potri.T118500, PtFLA24, PtFLA4
*Potri.019G121100 (POPTR_0019s14370)*	PtFLA45	Chimeric	10/9/2/1/0/1	41 %	262	PF02469.20	Y	N		AtFLA11, AtFLA12, AtFLA13, AtFLA9, AtFLA6	PtFLA35, PtFLA39, PtFLA13, PtFLA28, PtFLA47
*Potri.009G012100 (POPTR_0009s01730)*	PtFLA46	Chimeric	6/7/2/0/1/2	36 %	263	PF02469.20	Y	N	Xylem	AtFLA11, AtFLA12, AtFLA9, AtFLA13, AtFLA6	PtFLA2, PtFLA48, PtFLA27, PtFLA28, PtFLA10
*Potri.013G151500 (POPTR_0013s14790)*	PtFLA47	Chimeric	8/9/2/2/0/2	42 %	264	PF02469.20	Y	N	Xylem	AtFLA12, AtFLA11, AtFLA13, AtFLA9, AtFLA6,	PtFLA28, PtFLA39, PtFLA40, PtFLA50, PtFLA32
*Potri.015G013300 (POPTR_0015s01560)*	PtFLA48	Chimeric	7/5/2/0/1/3	36 %	267	PF02469.20	Y	Y	Xylem	AtFLA11, AtFLA12, AtFLA13, AtFLA9, AtFLA6	PtFLA27, PtFLA23, PtFLA10, PtFLA2, PtFLA34
*Potri.019G121300*	PtFLA49	Chimeric	10/9/1/1/0/2	42 %	263	PF02469.20	Y	Y		AtFLA11, AtFLA12, AtFLA9, AtFLA13, AtFLA6	PtFLA32, PtFLA50, PtFLA28, PtFLA39, PtFLA29
*Potri.019G123100*	PtFLA50	Chimeric	8/8/3/1/0/2	42 %	263	PF02469.20	Y	Y		AtFLA11, AtFLA12, AtFLA13, AtFLA9, AtFLA6	PtFLA29, PtFLA32, PtFLA49, PtFLA28, PtFLA39
Potri.011G117800 (POPTR_0011s11860)	PtPAG1	Chimeric	10/10/22/9/4/3	52 %	343	PF02298.15	Y	Y	Roots	AtPAG17, AtPAG11, AtPAG10, AtPAG14, AtPAG7	PtPAG5, PtPAG6, PtPAG7, PtPAG8, PtPAG9
Potri.006G067300 (POPTR_0006s06640)	PtPAG2	Chimeric	9/13/13/13/1/0	54 %	322	PF02298.15	Y*	Y	Male catkins	AtPAG4, AtPAG3, AtPAG5, AtPAG16, AtPAG7	PtPAG3, PtPAG10, PtPAG11, PtPAG4, PtPAG12
Potri.018G129200 (POPTR_0018s12930)	PtPAG3	Chimeric	4/7/14/12/0/0	60 %	250	PF02298.15	Y	Y	Roots	AtPAG5, AtPAG4, AtPAG7, AtPAG17, AtPAG3	PtPAG2, PtPAG10, PtPAG11, PtPAG4, PtPAG12
Potri.018G129400 (POPTR_0018s12950)	PtPAG4	Chimeric	1/1/3/4/1/0	50 %	183	PF02298.15	Y	Y		AtPAG16, AtPAG5, AtPAG7, AtPAG3, AtPAG8	PtPAG11, PtPAG10, PtPAG13, PtPAG2, PtPAG3
Potri.001G398800 (POPTR_0001s40940)	PtPAG5	Chimeric	15/11/23/8/5/3	51 %	377	PF02298.15	Y	Y	Light-grown seedling, young leaf	AtPAG17, AtPAG11, AtPAG10, AtPAG14, AtPAG7	PtPAG1, PtPAG6, PtPAG7, PtPAG9, PtPAG14
*Potri.017G011200 (POPTR_0017s04390)*	PtPAG6	Chimeric	1/3/5/2/2/0	33 %	212	PF02298.15	Y	Y		AtPAG11, AtPAG14, AtPAG17, AtPAG10, AtPAG7	PtPAG7, PtPAG1, PtPAG5, PtPAG16, PtPAG14
*Potri.017G012300 (POPTR_0017s00580)*	PtPAG7	Chimeric	1/3/5/2/2/0	33 %	212	PF02298.15	Y	Y		AtPAG11, AtPAG14, AtPAG17, AtPAG10, AtPAG7	PtPAG6, PtPAG1, PtPAG5, PtPAG16, PtPAG14
*Potri.011G135400 (POPTR_0011s13870)*	PtPAG8	Chimeric	2/2/3/2/2/2	35 %	208	PF02298.15	Y	Y	Roots, young leaf	AtPAG7, AtPAG13, AtPAG2, AtPAG12, AtPAG17	PtPAG14, PtPAG16, PtPAG1, PtPAG5, PtPAG15
*Potri.018G018200 (POPTR_0018s02630)*	PtPAG9	Chimeric	1/2/2/0/2/0	26 %	178	PF02298.15	Y	Y	Young leaf	AtPAG13, AtPAG2, AtPAG15, AtPAG12, AtPAG1	PtPAG16, PtPAG15, PtPAG1, PtPAG5, PtPAG6
*Potri.001G192100 (POPTR_0001s19280)*	PtPAG10	Chimeric	2/1/5/3/1/1	41 %	210	PF02298.15	Y	Y	Male catkins	AtPAG2, AtPAG4, AtPAG3, AtPAG16, AtPAG7	PtPAG2, PtPAG3, PtPAG4, PtPAG11, PtPAG17
*Potri.006G067400 (POPTR_0006s06650)*	PtPAG11	Chimeric	0/1/3/0/1/0	39 %	163	PF02298.15	Y	Y	Light-grown seedling	AtPAG16, AtPAG5, AtPAG8, AtPAG3, AtPAG13	PtPAG4, PtPAG2, PtPAG3, PtPAG10, PtPAG13
*Potri.003G047300 (POPTR_0003s04580)*	PtPAG12	Chimeric	1/0/4/2/1/2	35 %	217	PF02298.15	Y	Y	Female catkins	AtPAG16, AtPAG4, AtPAG5, AtPAG3, AtPAG8	PtPAG18, PtPAG19, Potri.006G259100, PtPAG20, Potri.006G259000
*Potri.014G049600 (POPTR_0014s04850)*	PtPAG13	Chimeric	2/1/1/5/1/1	48 %	192	PF02298.15	Y	Y	Dark etiolated seedlings	AtPAG9, AtPAG8, AtPAG6, AtPAG3, AtPAG5	PtPAG21, PtPAG22, PtPAG290, PtPAG23, PtPAG12
*Potri.001G419200 (POPTR_0001s44510)*	PtPAG14	Chimeric	4/5/2/3/0/2	35 %	221	PF02298.15	Y	Y	Roots	AtPAG7, AtPAG17, AtPAG15, AtPAG11, AtPAG12	PtPAG8, PtPAG15, PtPAG6, PtPAG1, PtPAG7
*Potri.006G184100 (POPTR_0006s19770)*	PtPAG15	Chimeric	2/2/3/0/2/0	29 %	178	PF02298.15	Y	Y		AtPAG13, AtPAG2, AtPAG15, AtPAG12, AtPAG1	PtPAG16, PtPAG9, PtPAG8, PtPAG14, PtPAG1
*Potri.006G264600 (POPTR_0006s28040)*	PtPAG16	Chimeric	2/3/3/0/2/0	28 %	179	PF02298.15	Y	Y	Young leaf	AtPAG13, AtPAG2, AtPAG15, AtPAG1, AtPAG12	PtPAG9, PtPAG15, PtPAG8, PtPAG1, PtPAG6
*Potri.013G061300 (POPTR_0013s05800)*	PtPAG17	Chimeric	2/2/3/1/0/1	29 %	155	PF02298.15	Y	N	Female catkins, male catkins	AtPAG5, AtPAG4, AtPAG3, AtPAG16, AtPAG13	PtPAG39, PtPAG24, PtPAG25, PtPAG26, PtPAG27
*Potri.002G161300 (POPTR_0002s16270)*	PtPAG18	Chimeric	2/2/2/0/1/0	31 %	169	PF02298.15	Y	Y	Male catkins	AtPAG16, AtPAG4, AtPAG3, AtPAG5, AtPAG13	PtPAG19, Potri.002G156100, Potri.002G156400, Potri.006G259000, Potri.006G259100
*Potri.001G268700 (POPTR_0001s27560)*	PtPAG19	Chimeric	1/2/4/0/0/0	31 %	165	PF02298.15	Y	Y	Male catkins	AtPAG16, AtPAG4, AtPAG3, AtPAG5, AtPAG13	PtPAG18, Potri.002G156100, Potri.002G156400, Potri.006G259000, PtPAG20
*Potri.002G052500 (POPTR_0002s05340)*	PtPAG20	Chimeric	0/1/2/0/1/0	28 %	169	PF02298.15	Y	Y	Young leaf	AtPAG16, AtPAG4, AtPAG3, AtPAG5, AtPAG13	PtPAG18, PtPAG19, Potri.002G156100, Potri.002G156400, Potri.006G259000
*Potri.001G080700 (POPTR_0001s11680)*	PtPAG21	Chimeric	1/2/0/0/0/1	30 %	184	PF02298.15	Y	Y		AtPAG5, AtPAG8, AtPAG9, AtPAG16, AtPAG3	PtPAG22, PtPAG13, PtPAG28, PtPAG23, PtPAG290
*Potri.003G150300 (POPTR_0003s15000)*	PtPAG22	Chimeric	1/1/1/0/0/0	31 %	183	PF02298.15	Y	Y		AtPAG5, AtPAG16, AtPAG8, AtPAG3, AtPAG4	PtPAG21, PtPAG13, PtPAG28, PtPAG23, PtPAG290
*Potri.002G101300 (POPTR_0002s10170)*	PtPAG23	Chimeric	0/1/3/1/0/4	42 %	188	PF02298.15	Y	Y	Xylem	AtPAG5, AtPAG8, AtPAG6, AtPAG3, AtPAG9	PtPAG290, PtPAG13, PtPAG12, PtPAG22, PtPAG24
*Potri.013G030000 (POPTR_0013s03090)*	PtPAG24	Chimeric	0/1/3/2/1/3	31 %	168	PF02298.15	Y	Y	Male catkins	AtPAG5, AtPAG4, AtPAG3, AtPAG16, AtPAG13	PtPAG25, PtPAG30, PtPAG26, PtPAG27, Potri.001G114200
*Potri.013G030200 (POPTR_0986s00200)*	PtPAG25	Chimeric	0/1/3/2/1/3	31 %	168	PF02298.15	Y	Y	Male catkins	AtPAG5, AtPAG4, AtPAG3, AtPAG16, AtPAG13	PtPAG24, PtPAG30, PtPAG26, PtPAG27, Potri.001G114200
*Potri.019G037800*	PtPAG26	Chimeric	1/1/1/2/0/0	32 %	155	PF02298.15	Y	Y		AtPAG5, AtPAG16, AtPAG4, AtPAG9, AtPAG3	PtPAG27, PtPAG39, PtPAG24, PtPAG25, PtPAG30
*Potri.T070900 (POPTR_0019s05370)*	PtPAG27	Chimeric	1/1/1/2/0/0	32 %	155	PF02298.15	Y	Y	Male catkins	AtPAG5, AtPAG16, AtPAG4, AtPAG9, AtPAG3	PtPAG26, PtPAG39, PtPAG24, PtPAG25, PtPAG30
*Potri.007G120200 (POPTR_0007s02750)*	PtPAG28	Chimeric	2/6/13/7/1/0	49 %	247	PF02298.15	Y	Y	Dark etiolated seedlings	AtPAG5, AtPAG17, AtPAG4, AtPAG3, AtPAG8	PtPAG21, PtPAG22, PtPAG13, PtPAG12, PtPAG31
*Potri.002G101200 (POPTR_1040s00200)*	PtPAG29	Chimeric	0/1/4/3/0/4	37 %	249	PF02298.15	Y*	Y		AtPAG5, AtPAG8, AtPAG3, AtPAG6, AtPAG9	PtPAG23, PtPAG13, PtPAG12, PtPAG22, PtPAG21
*Potri.003G117900 (POPTR_0003s11780)*	PtPAG30	Chimeric	0/0/6/1/0/2	33 %	167	PF02298.15	Y	Y	Male catkins, female catkins	AtPAG5, AtPAG4, AtPAG3, AtPAG16, AtPAG9	PtPAG24, PtPAG25, PtPAG26, PtPAG27, PtPAG17
*Potri.001G332200 (POPTR_0001s33960)*	PtPAG31	Chimeric	1/1/2/1/0/0	33 %	168	PF02298.15	Y	Y	Xylem	AtPAG5, AtPAG4, AtPAG3, AtPAG13, AtPAG16	PtPAG24, PtPAG25, Potri.009G136200, PtPAG28, PtPAG23
*Potri.008G151000 (POPTR_0008s15040)*	PtPAG32	Chimeric	3/1/2/0/1/3	35 %	185	PF02298.15	Y	N	Xylem	AtPAG16, AtPAG3, AtPAG4, AtPAG5, AtPAG13	PtPAG38, PtPAG18, Potri.006G259000, Potri.006G259100, PtPAG19
*Potri.017G088500 (POPTR_0017s12450)*	PtPAG33	Chimeric	2/2/1/1/0/0	23 %	175	PF02298.15	Y*	Y	Roots	AtPAG16, AtPAG9, AtPAG1, AtPAG5, AtPAG2,	Potri.001G219900, Potri.001G219800, Potri.017G088600, Potri.003G183300, Potri.001G043600
*Potri.015G114300 (POPTR_0015s12570)*	PtPAG34	Chimeric	0/2/0/0/0/1	20 %	131	PF02298.15	Y	N		AtPAG11, AtPAG7, AtPAG13, AtPAG2, AtPAG14	Potri.015G114700, Potri.015G113300, Potri.015G115600, Potri.015G117100, Potri.015G114600
*Potri.010G243600 (POPTR_0010s24980)*	PtPAG35	Chimeric	3/3/6/0/1/2	34 %	214	PF02298.15	Y	Y	Male catkins	AtPAG11, AtPAG5, AtPAG17, AtPAG2, AtPAG4,	PtPAG2, PtPAG4, PtPAG3, PtPAG18, PtPAG12
*Potri.001G187700 (POPTR_0001s18820)*	PtPAG36	Chimeric	1/1/2/2/1/0	27 %	181	PF02298.15	Y	Y	Male catkins, female catkins	AtPAG11, AtPAG7, AtPAG2, AtPAG17, AtPAG14	PtPAG37, Potri.015G052000, PtPAG8, PtPAG1, Potri.001G338800
*Potri.003G050500 (POPTR_0003s04900)*	PtPAG37	Chimeric	2/0/2/1/0/0	26 %	180	PF02298.15	Y	Y		AtPAG17, AtPAG2, AtPAG13, AtPAG7, AtPAG15	PtPAG36, Potri.015G052000, PtPAG15, Potri.001G338800, PtPAG1
*Potri.010G089900 (POPTR_0010s10020)*	PtPAG38	Chimeric	1/2/2/1/1/2	34 %	185	PF02298.15	Y	N	Xylem	AtPAG16, AtPAG3, AtPAG4, AtPAG5, AtPAG13	PtPAG32, PtPAG18, Potri.006G259000, Potri.006G259100, Potri.002G156100
*Potri.013G054500 (POPTR_0013s05140)*	PtPAG39	Chimeric	2/1/0/1/0/0	29 %	156	PF02298.15	Y	N	Female catkins	AtPAG5, AtPAG16, AtPAG4, AtPAG3, AtPAG9	PtPAG26, PtPAG27, PtPAG24, PtPAG25, PtPAG17
*Potri.002G092800 (POPTR_0002s09340)*	PtAGP57I	Chimeric	10/7/3/0/0/1	46 %	193	PF14368.4	Y	N		AtAGP29I	PtAGP60I, PtAGP64C, PtAGP58I, PtAGP61I, PtAGP69C
*Potri.003G020200 (POPTR_0003s01440)*	PtAGP58I	Chimeric	6/5/2/1/1/0	43 %	179	PF14368.4	Y	Y	Xylem, young leaf	AtAGP29I	PtAGP61I, PtAGP60I, PtAGP64C, PtAGP57I, PtAGP68C
*Potri.006G261800 (POPTR_0006s27770)*	PtAGP59I	Chimeric	3/11/9/5/2/4	36 %	484	PF00704.26	Y	N	Male catkins	None	Potri.018G112100, Potri.006G188400, Potri.006G188300, Potri.018G111600, Potri.006G262000
*Potri.005G167500 (POPTR_0005s16550)*	PtAGP60I	Chimeric	10/9/4/1/0/3	48 %	216	PF14368.4	Y	N	Male catkins, female catkins	AtAGP29I	PtAGP64C, PtAGP57I, PtAGP58I, PtAGP61I, PtAGP69C
*Potri.001G210100 (POPTR_0001s21750)*	PtAGP61I	Chimeric	8/5/3/0/0/0	41 %	178	PF14368.4	Y	Y	Young leaf	AtAGP29I, AtAGP3C	PtAGP58I, PtAGP60I, PtAGP64C, PtAGP57I, Potri.001G231400
*Potri.010G085200 (POPTR_0010s09530)*	PtAGP62I	Chimeric	4/1/6/5/2/4	47 %	216	PF14368.4	Y	Y	Male catkins	AtAGP29I	PtAGP65C, PtAGP66C, PtAGP67C, PtAGP68C, PtAGP69C
*Potri.005G003500 (POPTR_0005s00550)*	PtAGP63I	Chimeric	7/15/6/9/0/5	41 %	624	PF07983.11	Y	Y		AtPRP13, AtPEX4	Potri.013G003500, PtAGP70I, PtAGP71I, PtAGP72I, PtAGP73I
*Potri.002G059600 (POPTR_0002s06050)*	PtAGP70I	Chimeric	0/1/4/7/0/3	47 %	255	PF07983.11	Y	N		AtPRP13	PtAGP73I, PtAGP71I, PtAGP72I, PtAGP63I, Potri.011G094400
*Potri.001G353400 (POPTR_0001s34420)*	PtAGP71I	Chimeric	1/7/5/9/1/5	49 %	286	PF07983.11	Y	N		AtPRP13	PtAGP72I, PtAGP70I, PtAGP73I, PtAGP63I, Potri.013G003500
*Potri.011G078500 (POPTR_0011s02870)*	PtAGP72I	Chimeric	1/7/5/10/1/1	46 %	304	PF07983.11	Y	Y		AtPRP13	PtAGP71I, PtAGP70I, Potri.013G003500, PtAGP63I, PtAGP73I
*Potri.005G202400*	PtAGP73I	Chimeric	1/2/4/5/0/3	44 %	261	PF07983.11	Y	N		AtPRP13	PtAGP70I, PtAGP71I, PtAGP72I, PtAGP63I, Potri.013G003500

^a^ Protein identifiers of the version 2.0 are shown in the parenthesis. Italics indicates a protein that was identified only by a BLAST search

^b^ The domains indicated by the Pfam number are: PF14368.4, LTP_2 domain (Probable lipid transfer); PF06376.10, AGP domain (Arabinogalactan peptide); PF02469.20, Fasciclin domain (Fasciclin domain); PF02298.15, Cu_bind_like domain (Plastocyanin-like domain); PF00704.26, Glyco_hydro_18 domain (Glycoside hydrolase family 18); PF07983.11, X8 domain (X8 domain)

^c^ Asterisk indicates a protein that is predicted to have a signal peptide either using the sensitive mode in the SignalP website or only if amino acids at the N terminus are discarded

^d^ Expression data are shown only when available at http://bar.utoronto.ca/efppop/cgi-bin/efpWeb.cgi

^e^ A locus ID indicates that it is not identified as an HRGP

The vast majority (97 %) of the identified AGPs were predicted to have a signal peptide and many (70 %) were predicted to have a GPI anchor, both of which are characteristic features of the AGP family. Of the 162 AGPs identified, only four FLAs were predicted to lack a signal peptide. A total of 114 of the 162 AGPs (70 %) were predicted to have a GPI anchor addition sequence. BLAST searches against the Arabidopsis protein database found that all but 21 of the putative AGPs were similar to at least one known Arabidopsis AGP, providing further evidence that these proteins are likely AGPs.

### Extensins (EXTs)

**Fig. 3:**
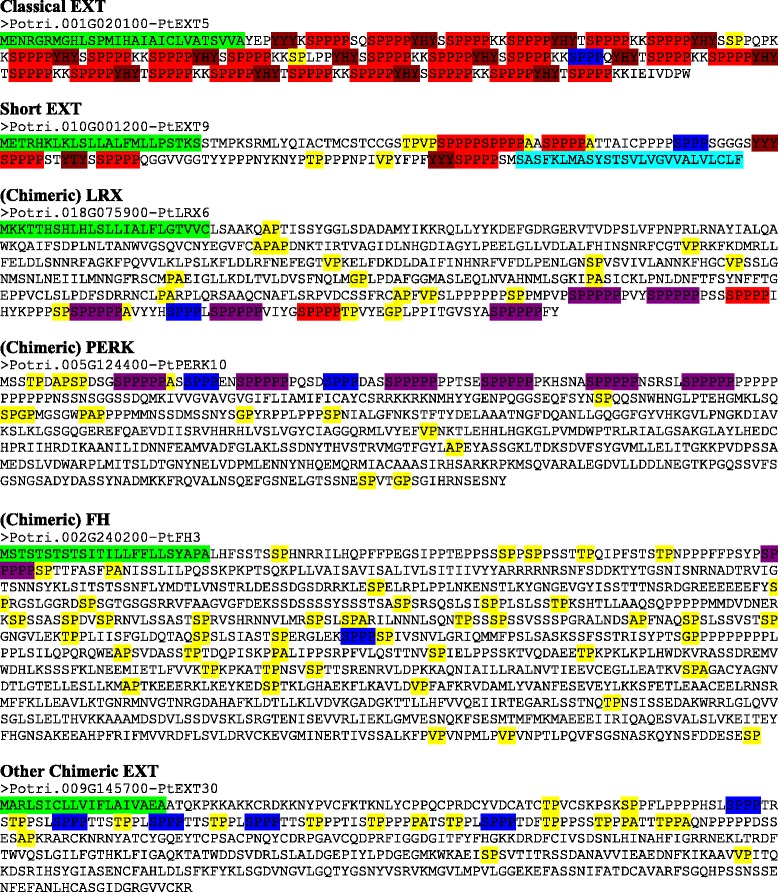
Protein sequences encoded by the representative EXT gene classes in *Populus trichocarpa*. The colored sequences at the N and C terminus indicate predicted signal peptides (green) and GPI anchor addition sequences (light blue) if present in the sequences. The SP3 (blue), SP4 (red), SP5 (purple), and YXY (dark red) repeats are also indicated in the sequences. The sequences typical of AGPs, specifically AP, PA, SP, TP, VP, and GP repeats, are also indicated (yellow)

Poplar had a smaller number of the classical EXTs containing large numbers of SPPPP repeats compared to Arabidopsis. For instance, a search for proteins with at least 15 SPPPP repeats in Arabidopsis found 21 “hits” while a similar search in poplar yielded only six, two of which are chimeric EXTs. The largest number of SPPPP repeats found in a single protein in poplar is 25, while in Arabidopsis one EXT contains 70 SPPPP repeats. Interestingly, although the abundance of these classical EXTs is decreased, many chimeric EXTs found in Arabidopsis were also in poplar in similar numbers, including the leucine-rich repeat extensins (LRXs) and proline-rich extensin-like receptor protein kinases (PERKs). By searching for proteins that contain at least two SPPP repeats, 162 poplar proteins were identified (Table 1). In all, 59 proteins identified in the search criteria were determined to be EXTs (Table 3). The only exception is a short EXT (i.e., Potri.T139000 or PtEXT33) identified by a BLAST search with one SPPPP that is homologous to several other short EXTs. These 60 proteins included 8 classical EXTs, 22 Short EXTs, 10 LRXs, 12 PERKs, 5 Formin Homology proteins (FHs), and 3 other chimeric EXTs (Fig. 3 and Additional file 2: Figure S2). YXY repeats were observed in 45 % of the EXT sequences; such sequences are involved in cross-linking EXTs [29–33]. Twenty-seven of the 60 EXTs identified contained YXY sequences in which X is quite variable. In contrast, 40 of the 59 EXTs in Arabidopsis (i.e., 68 %) contained YXY sequences in which X was often V [16]. Many of the classical EXTs and some of the LRXs also contained a SPPPP or SPPPPP sequence and Y residue at the C-terminus of their sequences as previously observed in Arabidopsis EXTs [33].

**Table 3:** Identification and analysis of EXT genes in *Populus trichocarpa*

Locus Identifier 3.0 (ID 2.0)^a^	Name	Class	SP3/SP4/SP5/YXY Repeats	Amino Acids	Pfam^b^	SP^c^	GPI	Organ/issue-specific Expression^11^	Arabidopsis HRGP BLAST Hits	Poplar HRGP BLAST Hits^e^
Potri.018G050100 (POPTR 0018 s05480)	PtEXT1	Classical EXT	1/6/4/5	190	PF04554.11	Y	N	Young leaf	AtEXT22, AtEXT21	Potri.001G201800
Potri.001G019700 (POPTR 0001 s05720)	PtEXT2	Classical EXT	1/21/0/11	213		Y	N		AtEXT3/5	PtEXT8
Potri.001G122100 (POPTR_0001 s00420)	PtEXT3	Classical EXT	2/5/6/0	238	PF14547.4	Y	N	Male catkins	AtPRP16, AtPRP15, AtPRP14, AtHAE4	Potri.013G128800, Potri.002G200100, Potri.018G025900, Potri.001G158400, Potri.014G059800
Potri.001G259600 (POPTR 0001 s26690)	PtEXT4	Classical EXT	2/8/2/0	500		Y	N		AtAGP51C	PtEXT7, AGP6C, AGP43P
Potri.001G020100 (POPTR 0001 s05740)	PtEXT5	Classical EXT	1/22/0/13	257		Y	N		None	PtEXT6, PtEXT8
Potri.001G019900	PtEXT6	Classical EXT	1/25/0/14	259		Y*	N		None	PtEXT8, PtEXT5
Potri.001G260200 (POPTR_0001 s26680)	PtEXT7	Classical EXT	4/6/1/0	222		Y	N		None	AGP43P, AGP6C, PtEXT4, Potri.003G074200
Potri.001G020000	PtEXT8	Classical EXT	1/23/0/16	267		Y*	N		AtEXT3/5	PtEXT6, PtEXT5
Potri.010G001200 (POPTR_0010s003 50)	PtEXT9	Short EXT	1/6/0/3	174		Y	Y		AtEXT37, AtEXT41	PtEXT24, Potri.008G129200, Potri.010G128900, Potri.008G117500, FLA21
Potri.010G113300 (POPTR_0010s12360)	PtEXT10	Short EXT	0/2/0/0	131		Y	N		AtEXT31, AtEXT33	PtEXT23, Potri.006G106800, Potri.005G033000, Potri.001G371600, PossiblePtEXT5
Potri.T091000	PtEXT11	Short EXT	1/1/0/0	106		Y	N		None	PtEXT12, PtEXT19, Potri.005G079400
Potri.013G045700 (POPTR 0013 s04290)	PtEXT12	Short EXT	1/1/0/0	111		Y	N		None	PtEXT11, PtEXT19
Potri.003G064900 (POPTR_0003 s063 50)	PtEXT13	Short EXT	1/1/3/0	167		Y	N		AtEXT32, AtAGP57C, AtPERK5	PtEXT26, Potri.009G013500, Potri.006G276200
Potri.006G225400 (POPTR_0006s24190)	PtEXT14	Short EXT	2/0/1/3	186		Y	Y	Male catkins, roots	AtEXT38, AtEXT7	Potri.015G147200, Potri.008G168300, Potri.010G094700, Potri.012G144400, PtFH2
Potri.002G070100	PtEXT15	Short EXT	0/1/2/2	102		Y	N		AtEXT3/5, AtEXT1/4, AtEXT22	PtEXT20, Potri.017G110900, PtEXT1, PtLRX3
Potri.019G015900 (POPTR_0019s03210)	PtEXT16	Short EXT	0/2/0/0	108		Y	N		None	PtEXT18, PtEXT33, PtEXT17, Potri.019G015700, Potri.T139100
Potri.019G015800 (POPTR_0019s03200)	PtEXT17	Short EXT	0/2/0/0	107		Y	N	Male catkins	None	PtEXT33, PtEXT18, PtEXT16, Potri.T139100, Potri.019G015700
Potri.019G016000	PtEXT18	Short EXT	0/2/0/0	116		Y	N		None	PtEXT16, PtEXT33, PtEXT17, Potri.019G015700, Potri.T139100
Potri.019G017300 (POPTR_0019s03400)	PtEXT19	Short EXT	0/2/0/0	110		Y*	N	Dark etiolated seedlings	AtPERK6, AtAGP45P	PtEXT11, PtEXT12, Potri.005G257000, Potri.010G244800, Potri.006G136900
Potri.005G190100	PtEXT20	Short EXT	1/2/0/2	115		Y	N		AtEXT3/5, AtEXT1/4, AtPRP3, AtPRP1	Potri.019G083200, Potri.013G112500, PtLRX3, Potri.007G090300, Potri.005G077700
Potri.014G124700	PtEXT21	Short EXT	0/2/0/0	168		Y	N		AtEXT34, AtEXT41, AtPERK3, AtPERK5	Potri.015G147200, Potri.012G144400, Potri.001G371600, Potri.004G143700, PtFH2
Potri.T082000	PtEXT22	Short EXT	1/1/1/0	177		Y*	N		None	PtAEH4, PtEXT28, PtEXT27, Potri.001G042100, Potri.008G043900
Potri.008G129100 (POPTR_0008s12800)	PtEXT23	Short EXT	0/3/0/0	155		Y	Y	Female catkins, xylem	AtEXT31, AtEXT33, AtPAG10	PtEXT10, Potri.010G094700, Potri.015G147200, Potri.006G163700, Potri.018G086100
Potri.008G213600 (POPTR_0008s22980)	PtEXT24	Short EXT	0/1/1/2	172		Y	Y	Male catkins	AtEXT37, AtPERK6, AtEXT41	PtEXT9, Potri.008G129200, PossiblePtEXT15, Potri.010G094700, Potri.004G143700
Potri.008G125400 (POPTR_0008s12430)	PtEXT25	Short EXT	2/0/0/0	80		Y*	N		None	Potri.005G239200, Potri.010G094700, Potri.010G006800, Potri.002G189300, Potri .005G239200
Potri.001G169200 (POPTR 0001 s16930)	PtEXT26	Short EXT	0/0/2/0	147		Y	N		None	PtEXT13, Potri.010G006800
Potri.001G042200 (POPTR 0001 s03370)	PtEXT27	Short EXT	2/2/0/1	177		Y	N		None	PtEXT28, PtEXT22, PtAEH4, Potri.001G042100, Potri.001G316500
Potri.T179500 (POPTR_0523s00220)	PtEXT28	Short EXT	1/0/1/0	176		Y*	N		None	PtAEH4, PtEXT22, PtEXT27, Potri.001G042100, Potri.005G030300
Potri.T101300 (POPTR_0017 s06820)	PtEXT29	Short EXT	0/2/0/0	151		Y*	N		AtAGP56C	Potri.007G120100, Potri.002G054100, Potri.001G371600, Potri.015G147200, Potri.002G235500
Potri.T139000	PtEXT33	Short EXT	0/1/0/0	107		Y	N		None	PtEXT17, PtEXT18, PtEXT16, Potri.019G015700, Potri.T139100
Potri.009G108100 (POPTR_0009s 11130)	PtLRX1	Chimeric	5/16/6/1	982	PF13855.4	Y	N	Female catkins	AtPEX3, AtPEX1, AtPEX4, AtPEX2, AtLRX4	PtLRX2, PtLRX10, PtLRX3, PtLRX6, PtLRX7
Potri.004G146400 (POPTR_0004s15360)	PtLRX2	Chimeric	2/19/1/1	603	PF13855.4	Y	N	Male catkins	AtPEX3, AtPEX4, AtPEX1, AtPEX2, AtLRX4	PtLRX1, PtLRX10, PtLRX3, PtLRX4, PtLRX7
Potri.006G081200	PtLRX3	Chimeric	2/1/3/0	584	PF13855.4 PF08263.10	Y*	N		AtLRX2, AtLRX1, AtLRX4, AtLRX3, AtLRX5	PtLRX7, PtLRX6, PtLRX4, PtLRX2, PtLRX10
Potri.006G245600 (POPTR_0006s26190)	PtLRX4	Chimeric	2/2/5/1	549	PF08263.10	Y	N	Xylem	AtLRX4, AtLRX3, AtLRX5, AtLRX7, AtLRX6	PtLRX8, PtLRX5, PtLRX9, PtLRX6, PtLRX3
Potri.006G162300 (POPTR_0024s00730)	PtLRX5	Chimeric	2/3/3/0	569	PF13855.4	Y	N	Male catkins	AtLRX4, AtLRX3, AtLRX2, AtLRX1, AtPEX4	PtLRX9, PtLRX6, PtLRX4, PtLRX8, PtLRX3
Potri.018G075900 (POPTR_0018s06150)	PtLRX6	Chimeric	1/2/5/0	509	PF13855.4	Y	N	Male catkins, young leaf, xylem	AtLRX3, AtLRX5, AtLRX2, AtLRX7, AtLRX1	PtLRX5, PtLRX9, PtLRX4, PtLRX8, PtLRX3
Potri.018G151000 (POPTR_0018s14790)	PtLRX7	Chimeric	1/6/1/0	481	PF08263.10 PF13855.4	Y	N	Male catkins	AtLRX2, AtLRX1, AtLRX4, AtLRX3, AtLRX5	PtLRX3, PtLRX6, PtLRX5, PtLRX9, PtLRX4
Potri.018G035100 (POPTR_0018s01010)	PtLRX8	Chimeric	0/3/2/1	496	PF08263.10	Y	N	Male catkins	AtLRX4, AtLRX3, AtLRX5, AtLRX7, AtLRX6	PtLRX4, PtLRX6, Potri.010G083000, PtLRX3, PtLRX7
Potri.T016600 (POPTR_0028s00200)	PtLRX9	Chimeric	2/3/4/0	573	PF13855.4	Y	N	Male catkins	AtLRX4, AtLRX3, AtLRX2, AtLRX1, AtPEX4	PtLRX5, PtLRX6, PtLRX8, PtLRX3, PtLRX7
Potri.014G036700 (POPTR_0014s03600)	PtLRX10	Chimeric	1/5/1/1	474	PF13855.4	Y	N	Male catkins	AtPEX3, AtPEX1, AtPEX4, AtPEX2, AtLRX4	PtLRX2, PtLRX1, PtLRX3, PtLRX7, Potri.007G139200
Potri.010G041400 (POPTR_0010s05110)	PtPERK1	Chimeric	5/0/2/1	700	PF07714.15	N	N		AtPERK13, AtPERK12, AtPERK11, AtPERK10, AtPERK8	PtPERK11,PtPERK3, PtPERK6, PtPERK3, PtPERK12
Potri.010G132900 (POPTR_0010s14290)	PtPERK2	Chimeric	5/4/2/1	765	PF00069.23	N	N		AtPERK8, AtPERK13, AtPERK1, AtPERK15, AtPERK4	PtPERK12, PtPERK11, PtPERK1, PtPERK8, PtPERK10
Potri.017G110400 (POPTR_0017s14140)	PtPERK3	Chimeric	5/5/0/1	724	PF07714.15	N	N	Dark etiolated and light-grown seedlings	AtPERK8, AtPERK10, AtPERK13, AtPERK12, AtPERK3	PtPERK6, PtPERK12, PtPERK2, PtPERK1, PtPERK11
Potri.009G115200 (POPTR_0009s 11810)	PtPERK4	Chimeric	1/6/2/1	649	PF07714.15	N	N	Male catkins	AtPERK5, AtPERK4, AtPERK15, AtPERK3, AtPERK13	PtPERK10, PtPERK9, PtPERK8, Potri.001G183000, Potri.T140000
Potri.004G153600 (POPTR_0004s16100)	PtPERK5	Chimeric	3/3/3/1	656	PF07714.15	N	N		AtPERK5, AtPERK7, AtPERK4, AtPERK6, AtPERK15	PtPERK4, PtPERK10, PtPERK9, PtPERK8, Potri.001G183000
Potri.004G105200 (POPTR_0004s10490)	PtPERK6	Chimeric	6/4/0/2	724	PF07714.15	N	N	Dark etiolated seedlings	AtPERK10, AtPERK12, AtPERK13, AtPERK3, AtPERK15	PtPERK3, PtPERK2, PtPERK1, PtPERK11, PtPERK10
Potri.006G242800	PtPERK7	Chimeric	2/0/0/1	706	PF07714.15	N	N		AtPERK1, AtPERK5, AtPERK14, AtPERK15, AtPERK3	PtPERK10, PtPERK9, Potri.001G183000, Potri.003G053300, Potri.T140000
Potri.018G081300 (POPTR_0018s08800)	PtPERK8	Chimeric	0/2/2/0	672	PF07714.15	N	N	Xylem	AtPERK1, AtPERK4, AtPERK5, AtPERK15, AtPERK6	Potri.001G183000, PtPERK10, PtPERK9, Potri.003G053300, PtPERK5
Potri.007G027000 (POPTR_0007s12680)	PtPERK9	Chimeric	2/3/5/1	639	PF07714.15	N	N		AtPERK5, AtPERK7, AtPERK6, AtPERK15, AtPERK13	PtPERK10, PtPERK8, PtPERK5, Potri.003G053300, Potri.T140000
Potri.005G124400 (POPTR_0005s12590)	PtPERK10	Chimeric	2/1/5/0	592	PF07714.15	N	N	Female catkins, male catkins	AtPERK4, AtPERK5, AtPERK7, AtPERK6, AtPERK1	PtPERK9, PtPERK8, PtPERK5, PtPERK4, Potri.001G183000
Potri.008G189700 (POPTR_0008s19400)	PtPERK11	Chimeric	5/3/1/1	733	PF07714.15	N	N	Male catkins	AtPERK13, AtPERK11, AtPERK8, AtPERK10, AtPERK15	PtPERK1, PtPERK3, PtPERK6, PtPERK12, PtPERK2
Potri.008G111600 (POPTR_0008s11080)	PtPERK12	Chimeric	0/6/2/1	728	PF07714.15	N	N		AtPERK13, AtPERK1, AtPERK5, AtPERK15, AtPERK3	PtPERK2, PtPERK1, PtPERK8, PtPERK11, Potri.001G183000
Potri.003G103800 (POPTR_0003 s10280)	PtFH1	Chimeric	1/0/2/0	1226	PF02181.21 PF10409.7	N	N	Female catkins, male catkins	None	Potri.018G019600, PtFH5, Potri.018G108000, Potri.006G263700, Potri.015G061000
Potri.011G131700 (POPTR 0011 s13510)	PtFH2	Chimeric	1/0/2/0	987	PF02181.21	Y	N	Roots	None	Potri.001G416100, Potri.007G119900, Potri.007G054900, PtFH4, Potri.017G009900
Potri.002G240200 (POPTR_0002s24130)	PtFH3	Chimeric	1/0/1/0	1066	PF02181.21	Y	N	Young leaf, male catkins	None	PtFH4, Potri.007G140200, Potri.017G009900, Potri.007G054900, Potri.013G017900
Potri.014G174700 (POPTR_0014s17310)	PtFH4	Chimeric	0/0/2/0	1071	PF02181.21	Y	N	Roots, light-grown seedling	AtPERK5	PtFH3, Potri.007G140200, Potri.017G009900, Potri.007G054900, Potri.013G017900
Potri.012G067900 (POPTR_0012s06980)	PtFH5	Chimeric	0/0/2/0	1400	PF10409.7 PF02181.21	N	N	Xylem, male catkins	None	Potri.015G061000, Potri.018G019600, Potri.006G185500, Potri.018G108000, PtFH1
Potri.009G145700 (POPTR_0009s14810)	PtEXT30	Chimeric	5/0/0/0	467	PF06830.9	Y	N	Male catkins, roots	AtEXT51	Potri.009G097400, Potri.012G145400, Potri.011G127900, Potri.009G012600, Potri.009G012500
Potri.014G115700 (POPTR_0014s11110)	PtEXT31	Chimeric	8/0/0/0	526	PF00295.15	Y*	N	Roots	None	Potri.002G190600, Potri.005G005500, Potri.013G005000, Potri.010G152000, Potri.008G100500
Potri.011G066900 (POPTR_0011s07300)	PtEXT32	Chimeric	0/1/2/2	498	PF00112.21 PF00396.16 PF08246.10	Y	N	Female catkins, male catkins	AtAGP4C	Potri.011G066800, Potri.004G057700, Potri.005G232900, Potri.014G024100, Potri.001G302100
Potri.004G024500	PtAEH1	AGP EXT Hybrid	0/1/1/1	673	PF01657.15 PF07714.15	Y	N		None	Potri.004G024600, PtAEH2, Potri.004G025800, Potri.011G028400, Potri.004G025900
Potri.004G024800	PtAEH2	AGP EXT Hybrid	0/1/1/1	678	PF01657.15 PF07714.15	Y	N		None	Potri.004G024600, Potri.004G025800, PtAEH1, Potri.011G028400, Potri.004G025900
Potri.003G082300 (POPTR_0003 s08030)	PtAEH3	AGP EXT Hybrid	2/0/0/0	188		Y*	Y	Dark and light-grown seedlings, young leaf	AtPRP1	Potri.005G191900, Potri.016G025300, Potri.004G162500, PossibleHybrid2, Potri.015G147200
Potri.003G184500	PtAEH4	AGP EXT Hybrid	1/1/1/0	177		Y*	N		None	PtEXT22, PtEXT28, PtEXT27, Potri.001G042100, Potri.019G047600

^a^ Protein identifiers of the version 2.0 are shown in the parenthesis. Italics indicates a protein that was identified only by a BLAST search

^b^ The domains indicated by the Pfam number are: PF04554.11, Extensin_2 domain (Extensin-like region); PF14547.4, Hydrophob_seed domain (Hydrophobic seed protein); PF13855.4, LRR_8 domain (Leucine rich repeat); PF08263.10, LRRNT_2 domain (Leucine rich repeat N-terminal domain); PF07714.15, Pkinase_Tyr domain (Protein tyrosine kinase); PF00069.23, Pkinase domain (Protein kinase domain); PF02181.21, FH2 domain (Formin Homology 2 Domain); PF10409.7, PTEN_C2 domain (C2 domain of PTEN tumour-suppressor protein); PF06830.9, Root_cap domain (Root cap); PF00295.15, Glyco_hydro_28 domain (Glycoside hydrolase family 28); PF00112.21, Peptidase_C1 domain (Papain family cysteine protease); PF00396.16, Granulin domain (Granulin); PF08246.10, Inhibitor_I29 domain (Cathepsin propeptide inhibitor domain); PF01657.15, Stress-antifung domain (Salt stress response/antifungal); PF07714.15, Pkinase_Tyr domain (Protein tyrosine kinase)

^c^ Asterisk indicates a protein that is predicted to have a signal peptide either using the sensitive mode in the SignalP website or only if amino acids at the N terminus are discarded

^d^ Expression data are shown only when available at http://bar.utoronto.ca/efppop/cgi-bin/efpWeb.cgi

^e^ A locus ID indicates that it is not identified as an HRGP

In addition to the presence of SPPP and SPPPP repeats, the presence of a signal peptide was another factor in determining if a protein was considered an EXT. As with the AGPs, all the potential EXTs identified by the search were examined for signal peptides and GPI anchors. Signal peptides are known to occur in EXTs, but certain chimeric EXTs, notably the PERKs, lack a signal peptide [34]. In total, 46 of the 60 EXTs (77 %) identified have a signal peptide. Only four EXTs with GPI anchor addition sequences were identified, all of which were classified as short EXTs. This novel class of short EXTs with GPI anchor addition sequences was also observed in Arabidopsis [16].

Because EXTs were identified by searching for proteins with at least two SPPP sequences, many proteins were identified that contain only a few SPPP or SPPPP repeats among a much larger protein sequence. Many of these potential chimeric EXTs are not included in Table 3, but the sequences are available in Additional file 3: Figure S3 for further review. These may in fact be chimeric EXTs, but many lack a signal peptide and have only a few SPPP or SPPPP repeats among a much larger protein that does not belong to a class of previously characterized chimeric EXTs, such as PERKs, LRXs, or FHs.

### Proline-rich Proteins (PRPs)

**Fig. 4:**
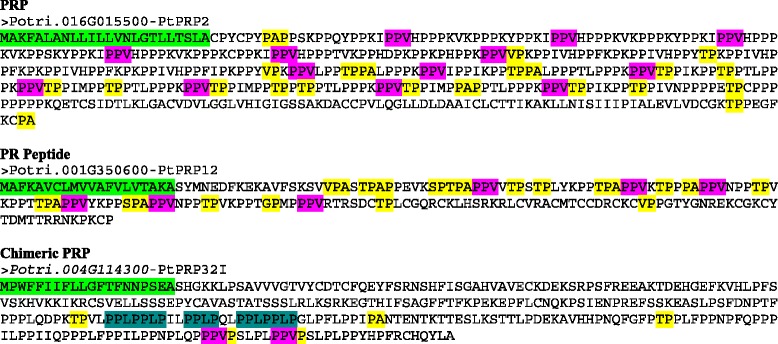
Protein sequences encoded by the representative PRP gene classes in *Populus trichocarpa*. The colored sequences at the N terminus indicate predicted signal peptides (green). PPV (pink) repeats typical of PRPs are indicated. The sequences typical of AGPs, specifically AP, PA, SP, TP, VP, and GP repeats, are also indicated (yellow) if present

PRPs were identified by searching for proteins that contain at least 45 % PVKCYT or contain two or more repeated motifs (PPVX[KT] or KKPCPP) (Table 1). Although this search generates a large number of false positives and proteins identified as AGPs and EXTs by other searches as described above, it was effective in the identification of PRPs in Arabidopsis [16]. Of the 240 poplar proteins meeting the 45 % PVKCYT criteria, 20 of the proteins were determined to be PRPs based on sequence analysis, the presence of a signal peptide, and BLAST analysis. The PPVX[KT] motif search returned 29 candidate proteins of which four were determined to be PRPs, while the other motif (KKPCPP) search returned no candidate protein despite its effectiveness in Arabidopsis (Table 4 and Additional file 4: Figure S4). Additional proteins were identified by BLAST searches that fall below the 45 % threshold. Some of these proteins were also determined to be PRPs based on a spectrum of information, including the presence of a signal peptide and Pfam domains, the number of motif repeats, and BLAST hits against Arabidopsis HRGPs. BLAST searches against the Arabidopsis database were particularly beneficial in determining if a protein was a PRP. In total, 49 proteins were determined as PRPs, including 16 PRPs, 30 PR-peptides, and three chimeric PRPs (Fig. 4 and Additional file 4: Figure S4). Indeed, each of the 49 putative PRPs identified here is similar to at least one PRP previously identified in Arabidopsis [16].

**Table 4:** Identification and analysis of PRP genes in *Populus trichocarpa*

Locus Identifier 3.0 (ID 2.0)^a^	Name	Class	% PVKCYT	PPV/PPLP/PELPK Repeats	Amino Acids	Pfam^b^	SP^c^	GPI	Organ/issue - Specific Expression^d^	Arabidopsis HRGP BLAST Hits	Poplar HRGP BLAST Hits^e^
Potri.004G168600 (POPTR 0004 s17590)	PtPRP1	PRP	64 %	24/8/0	554	PF01190.15	Y	N	Dark etiolated seedlings	AtPRP2, AtPRP1, AtPRP11	PtPRP6, PtPRP32, PtPRP33, PtPRP143, Potri.016G006200
Potri.016G015500 (POPTR_0016s01720)	PtPRP2	PRP	70 %	13/0/0	449	PF14547.4	Y	N	Dark and +3 h light etiolated seedlings	AtPRP18, AtPEX4	Potri.012G076700, Potri.015G071500, Potri.019G083900, Potri.T155100, Potri.005G239100
Potri.014G126200 (POPTR 0014 s12100)	PtPRP3	PRP	51 %	0/0/0	372	PF01190.15	Y	N		AtPRP9, AtPRP10	PtPRP24, PtPRP22, PtPRP28, PtPRP26, PtPRP21
Potri.014G126500 (POPTR_0014s12120)	PtPRP4	PRP	52 %	0/0/0	366	PF01190.15	Y	N		AtPRP7, AtPRP3, AtPRP1, AtAGP30I, AtAGP31I	PtPRP35, PtPRP3, PtPRP4, Potri.014G126300, PtPRP39
Potri.018G126000 (POPTR 0018 s12630)	PtPRP5	PRP	62 %	15/9/0	310	PF14547.4	Y*	N		AtPRP9, AtPRP10, AtPERK15	PtPRP44, PtPRP42, PtPRP41, PtPRP43, Potri.011G060200
Potri.009G129900 (POPTR 0009 s13250)	PtPRP6	PRP	48 %	2/1/0	283	PF01190.15	Y*	N		AtPRP9, AtPRP10, AtPRP1	Potri.019G082700, PtPRP21, PtPRP26, PtPRP18, PtPRP28
Potri.003G111300 (POPTR 0003 s11060)	PtPRP7	PRP	46 %	4/1/0	234	PF14547.4	Y*	N	Male catkins	AtPRP9, AtPRP10, AtPRP15	PtPRP27, PtPRP30, PtPRP21, PtPRP26, PtPRP22
Potri.006G008300	PtPRP8	PRP	59 %	8/0/0	234	PF14547.4	Y	N		AtPRP9, AtPRP10	PtPRP49, PtPRP26, PtPRP22, PtPRP23, PtPRP24
Potri.T162800 (POPTR 0006 s01030)	PtPRP9	PRP	50 %	2/0/0	216	PF14547.4	Y	N		AtPRP9, AtPRP10	PtPRP48, PtPRP26, PtPRP22, PtPRP28, PtPRP23
Potri.006G008600	PtPRP10	PRP	53 %	4/0/0	214	PF14547.4	Y	N	Young leaf	AtPRP16, AtPRP14, AtPRP17, AtPRP15, AtHAE4	PtPRP15, PtPRP13, PtPRP5, PtPRP11, Potri.018G025900
*Potri. 002G201800 (POPTR 0002 s20290)*	PtPRP34	PRP	37 %	0/0/0	213	PF01190.15	Y	N	Young leaf, male catkins	AtPRP9, AtPRP10	PtPRP22, PtPRP23, PtPRP26, PtPRP24, PtPRP29
*Potri. 017G145800 (POPTR 0017 s01230)*	PtPRP35	PRP	42 %	0/0/0	272	PF01190.15	Y	N		AtPRP9, AtPRP10	PtPRP22, PtPRP26, PtPRP21, PtPRP23, PtPRP24
*Potri. 001G060500 (POPTR_0001s13450)*	PtPRP38	PRP	39 %	0/7/0	332	PF01190.15	Y	N	Dark and +3 h light etiolated seedlings	AtPRP11, AtAGP31I, AtPRP1	PtPRP33, PtPRP36, Potri.001G326200, Potri.017G068400, PtPRP38
*Potri. 003G167100 (POPTR_0003s16550)*	PtPRP40	PRP	39 %	0/2/0	299	PF01190.15	Y	N	Female catkins	AtPRP7, AtPRP1, AtPRP3, AtAGP30I, AtAGP31I	PtPRP34, PtPRP4, PtPRP3, Potri.014G126300, PtPRP39
*Potri.007G114400*	PtPRP44	PRP	43 %	0/1/10	275		Y	N	Roots	AtPRP7, AtPRP3, AtPRP1, AtAGP30I, AtAGP31I	PtPRP34, PtPRP35, PtPRP4, PtPRP3, Potri.014G126300
*Potri. 013 G111600 (POPTR 0013 s11600)*	PtPRP46	PRP	39 %	0/4/0	216		Y	N		AtPRP9, AtPRP10, AtPERK5	PtPRP45, PtPRP44, PtPRP42, PtPRP43, PtPRP28
Potri.006G065500 (POPTR 0006 s06430)	PtPRP11	PR Peptide	56 %	5/2/0	198	PF14547.4	Y	N	Dark and +3 h light etiolated seedlings	AtPRP7, AtPRP3, AtPRP1, AtAGP30I, AtPRP9	PtPRP4, PossiblePtPRP6, Potri.002G201700, PtPRP34, PtPRP35
Potri.001G350600 (POPTR_0001s34750)	PtPRP12	PR Peptide	63 %	6/0/0	191	PF02704.12	Y	N		AtPRP7, AtPRP3, AtPRP1, AtPRP9, AtAGP30I	PtPRP3, PossiblePtPRP6, Potri.002G201700, PtPRP34, PtPRP35
Potri.T162900 (POPTR_0006s01020)	PtPRP13	PR Peptide	52 %	4/0/0	184	PF14547.4	Y	N	Young leaf	AtPRP15, AtPRP14, AtPRP17, AtPRP2, AtPRP1	PtPRP11, PtPRP7, PtPRP13, PtPRP15, PtPRP8
Potri.010G072200 (POPTR 0010 s08290)	PtPRP14	PR Peptide	50 %	6/0/0	179	PF02095.13	Y	N	Mature leaf	AtPRP2, AtPRP4, AtPRP11	PtPRP1.8, PtPRP32, PtPRP33, PtPRP36, Potri.005G041400
Potri.006G008500	PtPRP15	PR Peptide	53 %	4/0/0	179	PF14547.4	Y	N	Roots	AtPRP14, AtPRP15, AtPRP16, AtPRP17	PtPRP11, PtPRP5, PtPRP2, PtPRP13, PtPRP15
Potri.007G113900 (POPTR_0007s03420)	PtPRP16	PR Peptide	47 %	0/4/0	130		Y	N		AtPRP16, AtPRP17, AtPRP15, AtPRP14, AtHAE4	PtPRP15, PtPRP13, PtPRP9, PtPRP2, PtPRP11
Potri.007G114100 (POPTR_0007s03400)	PtPRP17	PR Peptide	46 %	0/3/0	119		Y	N		AtPRP16, AtPRP17, AtPRP14, AtPRP15, AtHAE4	PtPRP10, PtPRP13, PtPRP8, PtPRP2, PtPRP11
Potri.007G113700 (POPTR_0007s03440)	PtPRP18	PR Peptide	47 %	0/4/0	119		Y	N		AtPRP16, AtPRP17, AtPRP14, AtPRP15, AtAGP30I	PtPRP9, PtPRP13, PtPRP8, PtPRP2, PtPRP15
Potri.017G047400 (POPTR_0017s07470)	PtPRP19	PR Peptide	46 %	0/3/0	113		Y	N	Dark etiolated seedlings, light-grown seedling	AtPRP15, AtPRP14, AtPRP17, AtPRP2	PtPRP5, PtPRP7, PtPRP13, PtPRP15, PtPRP8
Potri.019G082600 (POPTR_0019s11220)	PtPRP20	PR Peptide	45 %	0/4/0	112		Y	N	light-grown seedling	AtPRP16, AtPRP17, AtPRP14, AtPRP15, AtHAE4,	PtPRP15, PtPRP8, PtPRP10, PtPRP9, PtPRP11
Potri.017G047200 (POPTR_0017s07450)	PtPRP21	PR Peptide	43 %	0/3/0	130		Y	N	Young leaf, male catkins	AtPRP1, AtPRP2, AtPEX4	Potri.004G110100, Potri.010G211100, Potri.004G109000, Potri.T018900, Potri.004G109900
Potri.017G045800 (POPTR_0017 s07310)	PtPRP22	PR Peptide	43 %	0/3/0	116		Y	N		AtPRP16, AtPRP17, AtPRP14, AtPRP15, AtHAE4, AtPERK5	PtPRP13, PtPRP10, PtPRP2, PtPRP9, PtPRP11
Potri.017G046700 (POPTR 0017 s07400)	PtPRP23	PR Peptide	40 %	0/3/0	116		Y	N		AtPRP9, AtPRP10, AtPRP15	PtPRP21, PtPRP26, PtPRP31, Potri.017G046800, PtPRP27
Potri.017G046400 (POPTR 0017 s07370)	PtPRP24	PR Peptide	43 %	0/3/0	116		Y	N	Roots	AtPRP9, AtPRP10	PtPRP21, PtPRP30, PtPRP27, Potri.017G046800, PtPRP18
Potri.017G045900 (POPTR 0017 s07320)	PtPRP25	PR Peptide	43 %	0/3/0	116		Y	N		AtPRP9, AtPRP10, AtPRP15	PtPRP19, PtPRP21, PtPRP27, PtPRP30, Potri.017G046800
Potri.017G047000 (POPTR_0017 s07430)	PtPRP26	PR Peptide	42 %	0/3/0	116		Y	N		AtPRP9, AtPRP10	PtPRP18, PtPRP21, Potri.017G046800, PtPRP27, PtPRP30
Potri.017G047100	PtPRP27	PR Peptide	44 %	0/4/0	134		Y	N	Female catkins	AtPRP9, AtPRP10, AtPRP15	PtPRP21, PtPRP18, PtPRP26, PtPRP37, PtPRP19
Potri.017G045600 (POPTR 0017 s07290)	PtPRP28	PR Peptide	44 %	0/3/0	126		Y	N	Roots	AtPRP9, AtPRP10	PtPRP30, Potri.017G046800, PtPRP27, PtPRP18, PtPRP17
Potri.017G046100 (POPTR 0017 s07340)	PtPRP29	PR Peptide	42 %	0/3/0	116		Y	N		AtPRP9, AtPRP10	PtPRP26, PtPRP25, PtPRP24, PtPRP23, PtPRP29
Potri.T178800 (POPTR 2000 s00200)	PtPRP30	PR Peptide	42 %	0/4/0	135		Y	N	Xylem	AtPRP9, AtPRP10	PtPRP22, PtPRP23, PtPRP26, PtPRP21, PtPRP28
Potri.007G114200 (POPTR 0007 s03390)	PtPRP31	PR Peptide	44 %	0/4/0	121		Y	N		AtPRP9, AtPRP10	PtPRP22, PtPRP26, PtPRP21, PtPRP23, PtPRP28
*Potri. 017G045000*	PtPRP37	PR Peptide	40 %	0/3/0	105		Y	N	Roots	AtPRP9, AtPRP10, AtPRP15	PtPRP16, PtPRP21, PtPRP26, Potri.017G046800, PtPRP27
*Potri. 002G201900 (POPTR_0002s20300)*	PtPRP39	PR Peptide	33 %	0/0/0	179	PF01190.15	Y	N		AtPRP11, AtAGP31I, AtPRP1	PtPRP32, PtPRP36, Potri.001G326200, Potri.017G068400, PtPRP38
*Potri. 017G044800 (POPTR_0017s07230)*	PtPRP41	PR Peptide	34 %	0/1/3	112		Y	N	Young leaf, male catkins	AtPRP11, AtPRP1, AtAGP31I, AtPRP2	PtPRP32, Potri .001G326200, Potri.017G068400, PtPRP38, PtPRP40
*Potri. 017G044900*	PtPRP42	PR Peptide	39 %	0/0/5	109		Y	N		AtPRP9, AtPRP10	PtPRP26, PtPRP21, PtPRP22, PtPRP28, PtPRP23
*Potri. 018G146200*	PtPRP43	PR Peptide	42 %	0/1/2	114		Y	N	Young leaf	AtPRP9	PtPRP40, Potri.017G068400, Potri.001G326200, PtPRP32, PtPRP33
*Potri.007G114700 (P0PTR_0007s03340)*	PtPRP45	PR Peptide	38 %	0/0/4	107		Y	N		AtPRP11	PtPRP38, Potri.017G068400, Potri.001G326200, PtPRP33, PtPRP32
*Potri. 017G046800 (POPTR 0017 s07440)*	PtPRP47	PR Peptide	41 %	0/5/0	174		Y*	N		AtPRP9, AtPRP10, AtPEX2	PtPRP45, PtPRP44, PtPRP43, PtPRP41, PtPRP18
*Potri. 017G045700 (POPTR 0017 s07300)*	PtPRP48	PR Peptide	38 %	0/2/0	97		Y	N		AtPRP9, AtPRP10	PtPRP44, PtPRP45, PtPRP42, PtPRP41, PtPRP37
*Potri. 017G046500 (POPTR 0017 s07380)*	PtPRP49	PR Peptide	38 %	0/3/0	97		Y*	N		AtPRP10, AtPRP9, AtPEX2	PtPRP45, PtPRP43, PtPRP42, PtPRP41, Potri.017G052100
*Potri. 004G114300 (POPTR 0004 s11300)*	PtPRP32I	Chimeric	41 %	2/5/0	319	PF01190.15	Y	N		AtPRP9, AtPRP10	PtPRP22, PtPRP21, PtPRP23, PtPRP28, PtPRP24
*Potri. 004G114400*	PtPRP33I	Chimeric	41 %	0/6/0	365	PF01190.15	Y	N		AtPRP9, AtPRP10	PtPRP30, Potri.017G046800, PtPRP21, PtPRP17, PtPRP18
*Potri. 017G100600 (POPTR_0017s13490)*	PtPRP36I	Chimeric	43 %	0/5/0	410	PF01190.15	Y	N		AtPRP9, AtPRP10	PtPRP27, PtPRP21, Potri.017G046800, PtPRP17, PtPRP18

^a^ Protein identifiers of the version 2.0 are shown in the parenthesis. Italics indicates a protein that was identified only by a BLAST search

^b^ The domains indicated by the Pfam number are: PF01190.15, Pollen_Ole_e_I domain (Pollen proteins Ole e I like); PF14547.4, Hydrophob_seed domain (Hydrophobic seed protein); PF02704.12, GASA domain (Gibberellin regulated protein); PF02095.13, Extensin_1 domain (Extensin-like protein repeat)

^c^ Asterisk indicates a protein that is predicted to have a signal peptide either using the sensitive mode in the SignalP website or only if amino acids at the N terminus are discarded

^d^ Expression data are shown only when available at http://bar.utoronto.ca/efppop/cgi-bin/efpWeb.cgi

^e^ A locus ID indicates that it is not identified as an HRGP

Interestingly, 30 short PRPs were identified in poplar, most of which contain a single SPPP repeat at the C-terminus. Nearly all of the 30 proteins show similarity to AtPRP9 and AtPRP10 based on BLAST searches. These novel 30 proteins were grouped into a new class known as the proline-rich peptides (PR peptides) due to their much shorter amino acid length compared to the typical PRPs identified. These PR peptides can be further subdivided based on the presence of two pentapeptide repeat sequences, PPLP and PELPK. The PPLP repeat is present in 23 of these PR peptides and in a few other PRPs and chimeric PRPs, while the PELPK repeat is found only in one PRP and four PR peptides including two that contain PPLP repeats. It is also interesting to note that the 23 genes encoding the PPLP-containing PR peptides are clustered on chromosome 17, while the genes encoding only the PELPK-containing PR peptides are clustered on chromosome 7. All of the 49 PRPs had a predicted signal peptide, while none had a GPI anchor predicted.

## Discussion

### A Bioinformatics Approach for Identifying HRGPs

As more plant genome sequencing projects are completed, vast amounts of biological data are being generated. Bioinformatics and in particular the BIO OHIO 2.0 program, which was recently revised and improved to provide a more rapid, reliable, and efficient method to identify proteins with biased amino acid compositions and known repetitive motifs [16, 22]. For instance, the BIO OHIO/Prot-Class program can search through over 73,000 proteins in the poplar proteomic database and identify those containing at least 50 % PAST in one minute. Using the various search criteria, we have predicted 271 HRGPs in poplar, including 162 AGPs, 60 EXTs, and 49 PRPs.

Although HRGPs were identified primarily through searching for biased amino acid compositions and repetitive motifs, the possibility that other HRGPs could be found in the poplar genome exists. Not all AGPs meet the 50 % PAST threshold, for instance, one classical AGP, PtAGP51C, contains only 49 % PAST. Similar problems exist for identifying chimeric AGPs. Because these proteins may contain only a small AGP region within a much larger sequence, they are likely to contain less than 50 % PAST. The possibility remains that other classes of chimeric AGPs or individual proteins that contain AGP-like regions exist and were not identified by the search parameters used in this study. A similar problem could exist for AG peptides that fall below the 35 % PAST cut-off or for PRPs that fall below 45 % PVKCYT.

One possible solution is to simply lower the thresholds and continue to search, but the number of false positives increases markedly as thresholds are lowered, making such searches less feasible. For instance, lowering the threshold for the AG peptide search to 30 % would identify 877 proteins compared to the 194 identified with a 35 % threshold.

In such a scenario, BLAST provides an alternative means to find additional candidate proteins. When using identified proteins as queries, BLAST is effective in finding a few related family members. For example, when using identified FLAs as queries, BLAST is capable of finding additional FLAs that don’t meet the criteria of the BIO OHIO 2.0 program. However, it is not particularly effective in finding other members of HRGP superfamily and thus could not be utilized in a comprehensive manner.

Indeed, a bioinformatics search that identifies HRGPs, especially chimeric HRGPs without also identifying a very large number of false positives remains difficult. Nevertheless, the search parameters and BLAST searches used here provide an efficient means to identify HRGPs and distinguish them from a limited number of false positive sequences. Of course, future molecular and biochemical analysis of the HRGPs predicted from this study will be necessary to validate these predictions more completely and elucidate their biological functions. Only when such work is completed will it become possible to conclusively distinguish HRGPs from false positive sequences.

### HRGPs exist as a spectrum of proteins

Although HRGPs are divided into AGPs, EXTs, and PRPs, the distinction between these categories is not always clear, since many HRGPs appear to exist as members of a spectrum of proteins rather than distinct categories. Indeed, several HRGPs identified here as well as some previously identified in Arabidopsis have characteristics of multiple families and can be considered hybrid HRGPs. For instance, many of the PRPs identified here, particularly some chimeric PRPs, also contain dipeptide repeats that are characteristic of AGPs. As such, it is difficult to determine if these should be considered as AGPs, PRPs, or classified as a hybrid HRGP. Determining whether these are actually AGPs or PRPs would depend on whether the proline residues are hydroxylated and subsequently glycosylated with arabinogalactan polysaccharides, which are characteristic of AGPs. Similarly, PtEXT4 also contains large numbers of characteristic AGP repeats (Additional file 2: Figure S2). In addition, BLAST searches revealed that it is similar in sequence to AtAGP51. Given that it contains many SPPP and SPPPP repeats, it was classified as an EXT. However, there is a possibility that this protein may also be glycosylated with the addition of AG polysaccharides, in which case it could potentially be grouped as a hybrid HRGP. Another example is the novel class identified here as the PR peptides (Table 4). Although grouped here as PRPs, these short sequences (i.e., PtPRP16-31 and PtPRP37) also contain a SPPP sequence characteristic of an EXT as well as the dipeptide repeats characteristic of AGPs, particularly AP, PA, and VP (Additional file 4: Figure S4).

Other difficulties arise when chimeric HRGPs are considered. For instance, the plastocyanins range from those that contain a majority of AGP repeats and easily pass the 50 % PAST test to those that contain only a few AP, PA, SP, VP, and GP repeats to those that contain no characteristic AGP repeats. The exact cutoff between proteins that are considered chimeric AGPs and those that are simply plastocyanin proteins is difficult to determine. Again, biochemical studies would be required to examine which of the proteins are actually glycosylated to make a final determination for classification. However, all those proteins annotated here as PAGs have at least a few characteristic AGP repeats, contain a signal peptide, and most have predicted GPI membrane anchor addition sequences, all of which is consistent with the chimeric AGP designation (Additional file 1: Figure S1).

A similar situation also exists for the chimeric EXTs, such as the PERKs and LRXs. How many SPPP or SPPPP repeats are required for a protein to be considered a LRX and not simply a leucine-rich repeat (LRR) protein? Here the cutoff was arbitrarily set to at least two repeats. As such, there may be LRR proteins that contain one SPPP that are not considered here as LRXs. Another example which illustrates this classification difficulty concerns the four proteins (PtAGP70I, PtAGP71I, PtAGP72I, and PtAGP73I) which are similar to AtPRP13 based on BLAST searches. However, these four proteins also contain numerous SP and AP repeats that would be more characteristic of an AGP. Exactly how proteins such as these should be classified is certainly debatable. Indeed it is human nature to group and classify items to facilitate understanding, while Mother Nature operates without such regard.

### Comparisons with previously identified poplar HRGPs

This study identified 271 poplar HRGPs (162 AGPs, 60 EXT, and 49 PRPs) in contrast to the 24 HRGPs (3 AGPs, 10 EXT, and 11 PRPs) identified by Newman and Cooper [18]. The more stringent search criteria for proline-rich tandem repeats and a less comprehensive poplar proteomic database based on EST and NCBI Non-Redundant protein sequences data from10/04/09 likely account for the fewer poplar HRGPs identified in this earlier study. In addition, homologs of the 15 FLA AGPs reported by Lafarguette et al. [20] in a *Populus tremula × P. alba* hybrid related to *Populus trichocarpa* were also identified in addition to 35 other FLAs. Thus, the present study represents the most comprehensive and detailed picture of the HRGP inventory in poplar to date.

### Comparisons with Arabidopsis

Findings here allow for a comparison of the HRGPs identified in Arabidopsis to those in poplar (Table 5). For AGPs, the classical AGPs identified in poplar showed a similar number as in Arabidopsis. Specifically, 27 classical AGPs including six lysine-rich AGPs were identified in poplar, while 25 classical AGPs including three lysine-rich AGPs were identified in Arabidopsis. Among other AGPs, particularly notable is the large increase the number of FLAs, PAGs, and AG peptides in poplar compared to Arabidopsis. While 21 FLAs, 17 PAGs and 16 AG peptides were identified in Arabidopsis, 50 FLAs, 39 PAGs and 35 AG peptides are identified here in poplar. There is also a noticeable increase in the number of other chimeric AGPs in poplar compared to Arabidopsis. Here, 11 other chimeric AGPs were identified in poplar, while only 6 were found in Arabidopsis.

**Table 5:** Comparison of HRGPs identified in *Populus trichocarpa* and *Arabidopsis thaliana*

HRGP family	HRGP subfamily	Poplar	Arabidopsis^a^
AGPs	Classical AGPs	21	22
	Lysine-Rich Classical AGPs	6	3
	AG-Peptides	35	16
	(Chimeric) FLAs	50	21
	(Chimeric) PAGs	39	17
	Other Chimeric AGPs	11	6
	All AGP subfamilies	162	85
EXTs	Classical EXTs	8	20
	Short EXTs	22	12
	(Chimeric) LRXs	10	11
	(Chimeric) FHs	5	6
	(Chimeric) PERKs	12	13
	Other Chimeric EXTs	3	3
	All EXT subfamilies	60	59
PRPs	PRPs	16	11
	PR Peptides	30	1
	Chimeric PRPs	3	6
	All PRP subfamilies	49	18
Total		271	168

^a^ The Arabidopsis HRGP data shown here are from Showalter et al. [16] with the exceptions that 6 chimeric FH EXTs were added and that one PR-peptide was found out of originally identified 12 PRPs as part of this study

Among EXTs, the classical EXTs with large numbers of SPPPP repeats are markedly decreased in poplar, while similar numbers of the chimeric EXTs exist in both species. The reduction in the number of classical EXTs in poplar is dramatic and likely indicates that many EXT genes or EXT functions are dispensable in poplar, and therefore not conserved in evolution. A similar loss of EXTs has also been observed in analysis of certain monocot species [unpublished data,18]. Moreover, far fewer poplar EXTs contain putative cross-linking YXY sequences compared to Arabidopsis, and this can be largely explained by the reduced number of classic EXT sequences, which typically contain such cross linking sequences. The various chimeric EXTs, namely the LRXs/PEXs, PERKs, and FHs, are conserved in both species. Although FHs were not reported in Showalter et al. [16], a reexamination of the Arabidopsis proteome shows 6 FH sequences (AtFH1-At3g2550, AtFH5-At5g54650, AtFH8-At1g70140, AtFH13-At5g58160, AtFH16-At5g07770, and AtFH20-At5g07740) contain two or more SPPP sequences. These 6 formins are included in Table 5 and are a subset of the 21 reported formins in Arabidopsis [35]. Similar to the chimeric EXTs, the short EXTs are also conserved in Arabidopsis and poplar. The short EXTs are a particularly interesting class because EXTs are not known to have GPI membrane anchors, a feature commonly found in many AGPs and associated with proteins found in lipid rafts [36]. The finding that several of these short EXTs encode a predicted GPI-anchor sequence are conserved in poplar and Arabidopsis certainly prompts the question of what role these proteins are playing in the plant. Currently, no publications verifying their biochemical existence or examining their roles exist, but this class stands out in terms of having interesting candidates for further investigation, particularly with respect to confirming their plasma membrane localization, hydroxylation, and glycosylation.

PRPs are similar in both species with the notable exception of the PR-peptides, which is a much expanded class in poplar compared to Arabidopsis, which is now recognized to have only one PR-peptide following a reexamination prompted by this study. All of the PR-peptides in poplar are similar in sequence with most containing LPPLP repeats and having a single SPPP repeat at the C terminus, although some contained PELPK repeats. In addition, most of these PR-peptides are similar to AtPRP9 and AtPRP10 based on BLAST analysis; both of these Arabidopsis proteins contain PELPK repeats as well. Indeed, AtPRP9 is quite short and similar in sequence to the PR peptides found in poplar but lacks the C terminal SPPP repeat. However, this is the only such protein found in Arabidopsis, while 30 were observed in poplar. AtPRP10 contains some similarity in sequence but is much longer than the poplar PR-peptides. Indeed, the large number of LPPLP- and PELPK- containing PR-peptides in poplar clustered respectively in two chromosomal locations indicates that these two gene subfamilies likely result from tandem gene duplication events, analogous to a unique, clustered set of PEHK-containing PRP genes in the grape family [18].

Although most sub-families of HRGPs exist in both the Arabidopsis and poplar inventories, certain species-specific differences do exist, which is reflected in the difference of number of certain groups and the total number of HRGPs (271 in poplar versus 168 in Arabidopsis). Precisely why certain classes of HRGPs are increased or decreased in abundance in a particular species remains to be determined, but these results lay the groundwork for future experimentation in this area.

### Poplar HRGPs genome 2.0 release and expression analysis

The study revealed that the poplar genome 3.0 release is quite different from 2.0 release in terms of HRGPs. Only 33 % of HRGPs identified in 3.0 are the same as counterparts in 2.0, others may differ from a few amino acids in sequence to a distinct start and/or stop position. For several such cases, a green highlight indicated a likely signal sequence placed internally, either because these signal sequences were at the N terminus in the 2.0 release or they should be at N terminus based on analysis of sequences in this study.

In addition, tissue/organ-specific HRGP expression data were obtained from the poplar eFP browser. However, this database does not contain all HRGP data, and it only accepts query IDs in poplar genome version 2.0 format. Judging from the available information, one could observe that HRGPs in general have high expression in seedlings, leaves, and reproductive tissues (Tables 2, 3, and 4). In particular, a number of FLAs were specifically expressed in xylem, while some PAGs were found to be highly expressed in male catkins. Many PRPs have high expression in seedlings and leaves. Interestingly, several LRXs are found to be uniquely expressed in male catkins; this finding is consistent with previous research in Arabidopsis and rice that a group of LRXs are pollen-specific LRXs, or PEXs [37].

### Pfam analysis of poplar HRGPs

All 271 poplar HRGPs identified in this study were subjected to Pfam analysis to identify specific domains within them. Pfam domains were found in 160 of the 271 proteins (59 %). More specifically, Pfam domains were identified in 105 of the 162 AGPs, 32 of the 62 EXTs, and 23 of the 49 PRPs. In particular, Pfam analysis exceled at finding domains within chimeric HRGPs, such as FLAs, PAGs, LRXs, PERKs, and FH EXTs. In contrast, such analysis often failed to find domains in classical AGPs or EXTs, possibly due to the variable sequences and numbers of sequence repeats associated with many of the HRGPs. Interestingly, many of the PRPs were found to contain Pollen Ole domains and Hydrophob seed domains. Pfam analysis also has merit in identifying domains in the chimeric HRGPs identified in the study. Indeed, while Pfam analysis alone is not sufficient for identifying HRGPs in a comprehensive manner, it can add valuable information to identified HRGPs, and thus a Pfam analysis module will likely be incorporated into future versions of the BIO OHIO program.

## Conclusions

The new and improved BIO OHIO 2.0 bioinformatics program was used to identify and classify the current inventory of HRGPs in poplar. This information will allow researchers to determine the structure and function of individual HRGPs and to explore potential industrial applications of these proteins in such areas as plant biofuel production, food additives, lubricants, and medicine. Other plant proteomes/genomes can also be examined with the program to provide their respective HRGP inventories and facilitate comparative evolutionary analysis of the HRGP family in the plant kingdom [16, 38]. Finally, while this program was specifically developed for HRGP identification, it can also be used to examine other plant or non-plant genomes/proteomes in order to identify proteins or protein families with any particular amino acid bias and/or amino acid sequence motif, making it useful throughout the tree domains and six kingdoms of life.

## Additional files

Additional file 1: Figure S1. Protein sequences encoded by the predicted AGP genes in *Populus trichocarpa*. The colored sequences at the N and C terminus indicate predicted signal peptides (green) and GPI anchor addition sequences (light blue) if present in the sequences. AP, PA, SP, TP, VP, and GP repeats (yellow) and lysine-rich regions (olive) are also indicated. Additionally, EXT SP_3_ (blue), SP_4_ (red), SP_5_ (purple) repeats and sequences typical of PRPs, PPV repeats, are indicated (pink) if present. Note that green font indicates a predicted signal peptide using the sensitive mode from the SignalP website. Internal green highlights indicate the presence of a predicted signal peptide only if amino acids at the N terminus are discarded. (PDF 69 kb)
Additional file 2: Figure S2. Protein sequences encoded by the predicted EXT genes in *Populus trichocarpa*. The colored sequences at the N and C terminus indicate predicted signal peptides (green) and GPI anchor addition sequences (light blue) if present in the sequences. The SP3 (blue), SP4 (red), SP5 (purple), and YXY (dark red) repeats are also indicated in the sequences. The sequences typical of AGPs, specifically AP, PA, SP, TP, VP, and GP repeats, are also indicated (yellow) in the sequences. Note that green font indicates a predicted signal peptide using the sensitive mode from the SignalP website. Internal green highlights indicate the presence of a predicted signal peptide only if amino acids at the N terminus are discarded. (PDF 72 kb)
Additional file 3: Figure S3. Protein sequences encoded by the potential chimeric EXT genes in *Populus trichocarpa*. The colored sequences at the N and C terminus indicate the predicted signal peptides (green) and GPI anchor addition sequences (light blue) if present in the sequences. The SP3 (blue), SP4 (red), SP5 (purple), and YXY (dark red) repeats are also indicated in the sequences. The sequences typical of AGPs, specifically AP, PA, SP, TP, VP, and GP repeats, are also indicated (yellow) in the sequences. (PDF 23 kb)
Additional file 4: Figure S4. Protein sequences encoded by the predicted PRP genes in *Populus trichocarpa*. The colored sequences at the N terminus indicate the predicted signal peptides (green). PPV (pink) repeats typical of PRPs are indicated. Repetitive motifs PPLP (teal) and PELPK (dark yellow) are also indicated. Additionally, EXT SP3 (blue) repeats, YXY (dark red) and sequences typical of AGPs, specifically AP, PA, SP, TP, VP, and GP repeats, are indicated (yellow) if present. Note that green font indicates a predicted signal peptide using the sensitive mode from the SignalP website. Internal green highlights indicate the presence of a predicted signal peptide only if amino acids at the N terminus are discarded. (PDF 47 kb)

## Abbreviations

AGPs: Arabinogalactan-proteins
EXTs: Extensins
FHs: Formin homology proteins
FLAs: Fasciclin-like AGPs
GPI: Glycosylphosphatidylinositol
HRGPs: Hydroxyproline-rich glycoproteins
LRXs: Leucine-rich repeat extensins
PAGs: Plastocyanin AGPs
PERKs: Proline-rich extensin-like receptor protein kinases
PRPs: Proline-rich proteins
