# Supplementary material for: Bioinformatic Identification and Analysis of Hydroxyproline-Rich Glycoproteins in Populus trichocarpa
Source: BMC Plant Biol. 2016 Oct 21;16:229. doi: 10.1186/s12870-016-0912-3 (PMC5073881; doi:10.1186/s12870-016-0912-3)
Supplement: Additional file 1: Figure S1. — Protein sequences encoded by the predicted AGP genes in Populus trichocarpa. The colored sequences at the N and C terminus indicate predicted signal peptides (green) and GPI anchor addition sequences (light blue) if present in the sequences. AP, PA, SP, TP, VP, and GP repeats (yellow) and lysine-rich regions (olive) are also indicated. Additionally, EXT SP3 (blue), SP4 (red), SP5 (purple) repeats and sequences typical of PRPs, PPV repeats, are indicated (pink) if present. Note that green font indicates a predicted signal peptide using the sensitive mode from the SignalP website. Internal green highlights indicate the presence of a predicted signal peptide only if amino acids at the N terminus are discarded. (PDF 69 kb) [file 12870_2016_912_MOESM1_ESM.pdf]

Supplemental Figure 1

>Potri.017G050200-PtAGP1C
MATSNILALVVLMVLFLTSTMAQSPTSSPTSSPTKSPVPPASTPTSAPPPTATSPPPTSSPPVSPPAPKKSPTTPPV
ASPPSPTTVSSPPPASSIPPSSISNSPSDAPGPSANDAVLNRVTVGGSLLAGLFAAVLVI

>Potri.017G050300-PtAGP2C
MAGNKSIVFLMLITFLASSTIAQPPASSPATSPSKSPPKASSPAPSTVKPPASAPSPLTTPPPSADAPSPVTTTSPP
SPPPETAPSSSPTGVPTSTISDTPAAAPGPNSGAVLNRFAFGGSVAVGVFAAVLVL

>Potri.005G161100-PtAGP3C
MAYKTLVCLMLLALLAGSALAQAPGAAPTAQPTKSPSPAPAAPTTPPPAPTPAPSVPAPTPAPSVPAPTPATAPSTS
PSSSPASSPPSPLAPGTGGGSIATPPSDTASPPSPSNADGLNRATMAGALIGVAVIIDCNCDLLDDQLDLNSHFDIL
TRETLSE

>Potri.014G135100-PtAGP4C
MASFCSTFLAMSTVFMAFFSSPALASQIHARISTISAAPAFLPVAPLSSPPTLSPDIEPLLPTPGAGAPSPTESSLP
TIPSSRSPPNPGDILAPGPARFSISPSGSLPASSSVSTTSSGPLNLAFFLGLLVLCSMQLSGV

>Potri.001G339700-PtAGP5C
MARQVLALALIFVAVVGAFAAEAPSATPLASPSPKASVAPAKAPTADTPKASASAPTISGAPSGSASSPVAEEGPAG
APEEEISSPPAPASLSAEGPAGPAAGPIADVVSDAPATPPAPQKDGAATIKISTAAVVAGLLVFLSF

>Potri.001G259700-PtAGP6C
MASPSRRHLVHAMVFGLLAINAAANIYDNEEPPSPPHDHEDPPLPYNHKNFVFQPSGLLNRGTPSPPRSQPPPNKKK
MPPPPPKIHHLQPPPQAKSPPPPPPAKSPTLPSASPPTPSSAPILPPLPSYISPVTSPSKVPPIPSPSPSPSPASNL
FPSPPYTSPASPPRISPIPSPSPFPSPNLSQSHSHTPPTSPSK

>Potri.001G310300-PtAGP7C
MASTSVVLMTLMAILLVGSTMARSSKSSPTPSPEAHVPAHSPTPAPASVPTPPSPSPSKAPPKAAPTPAPTVGSPPS
PPPSSTPAPANPTASYSPADAPKPSNAVLKKVSIGGVLSVGLFAAVLVA

>Potri.001G367600-PtAGP8C
MLQHMHSVLLLLVFAFCISNSNFTVNGDNAPAPSPTSSSIPKSPSSSSPSPSTTSPPSSPSKSPATSPSTSPPTLPV
SPSKSPATSPSPSTSLPTIPVSPSKSPATSPSPSTSLPTIPVSPSKSPATSPSSSTSPPSPTVSPPTPSPNAAAPSG
SPPASSPAVSTPVTAPTLAPLGAPPMSTEGPVTAATPEASASIPSSSATPAEAPMVFPSSSSPPSPSTESSMSPETA
KVPSVNDESGSRSLYEVGVILSGLVGGAALALAI

>Potri.001G310400-PtAGP9C
MAANKSMVFLMLITFLVASTKAQSPSSSPASSPTKSPPVATPPPKASAPAPTTVKPPASAPSPLETPPPAANAPSPT
TTTSPPSPLPVTPVPSTGDVPTSTIGSPAVAPAPANGAVLNRFALGGSVAVGVLAAVLVL

>Potri.017G047500-PtAGP10C
MASVHYLLIIAFFISIPLSCTNVALATRHLLHSAKNVPPLPRPKLPPLPPNLTSTPTTPVPTLPSPAETFGPPTLSL
VPPSISVLTPPNPPKTPPVTVESPTLPSPTSMSPPKDETPPSTSSKSPPLPNPISHKLPTLPKFPPVKSWPMPNPKS
LPTIPKFPHLKWPSLPPFHWIPKTLRVCTYHHVSGQRWRQQPPAVIVLSRLAT

>*Potri.002G207500*-PtAGP47C
MASFCSIFLAMFTVFMAYFSSPALTSQIHVQFSTISAAPAFLHDAPSFSPPTLSPDIEPLFPTPGVGAPSPTESSIP
TIPSNPSPPNPDDMLAPGPAGSSISPSGSLPAYSSVSLTSSGPLNLAVFLGLLVFCLVQPSGIM

>*Potri.010G031700*-PtAGP48C
DPILSTGTPFFLKQNFHYPHILLLCPSKCDIDFLIIRMASFCFFIVLIVPLMIFPLLSSSTQLNSKTSPYPISTSPP
FLTNPPPPSPLQELSPDIAPLLPSPGGVLPSPTVSSVPTIPSTPSPPNPDEVVAPGPASAFSPLGALPASSASPRNL
INFAIAVGCIAYWSI

>*Potri.008G182400*-PtAGP50C
MASKVIVLALVFVAIVGLASAAGPAPSTTALPAEAPLSDDFIGTDDAAAAGAPSGGDAVVPGPMGSVEAAGGPSGSP
KSDSAALKFSAITGVAAVAGYLFF

>*Potri.015G093700*-PtAGP51C
MARKLIVLALVFVTIVGLAAAAAPAPSTTDFPPAAAPAPSTTDFTAAEAPLSNDFIGTDDGGAASAPSADGTTVVPG
PMGSTAVAEGPSEKDGVATLKFSAVAGVAAVAGYFFFF

>*Potri.012G095900*-PtAGP52C
MAHKLIVLALVFVAIFGLAAAAAPAPSTTYLPAAAAPAPSTTDLPAGEAPLSHDFIGTNDGDAAGAPSADGSTVVPG
PMGSTTLAGGPASEKDGAATLKFSAIVGAAAVAGYFFF

>*Potri.005G169000*-PtAGP64C
MKRSLFIGCILATLALLANSAHHESSPRKSPAPSPSADCTDVAFDMLDCITYLSDGSEAAKPTASCCAGFEAVLSLD
AECLCFALKHSADFGVALNLTRAAALSSKCGVSAPPLSKCGISVPATGAPANPPSSVPEPAPPTESPPYPVIEPATN
NQPSAPAPAPSNSDDNGVSAAAPVIIEVPAQAPAKGMAYSISAPFSVLISCAVASTPLFLWV

>*Potri.008G155200*-PtAGP65C
MHHNFLHSSPTSIDPRYKNTHSHMERFVPFPRTVPFLAVALAVFVFPVYGQINAACTASVLATFAPCMTFLTSSTAN
GSSPTAGCCGSLKNLTSDGMDCLCLVVTGSVPFGVPINRTLAISLPRACNMPGVPVQCEATGAPIPAPAAVVPEPTP
SALPPASGTTPLLTPPSSTGDSGAPASTTGSHPVLTPPSASVPSDSLSPSLLLFALGFVLFKYYY

>*Potri.005G212000*-PtAGP66C
MEGLRVFNLIAILSTLLVISVNGQISTPCTTSMISSFTPCINFITGSTNNGSSPTGSCCSSFKSLMSTGMDCACLLI
TANVPLQLPINRTLAITLPRACKMSGVPMLCKASGTPLPAPGPVLLGPTLPPTAAYPLSPRASKAVALAPAPESEIT
LPLTPASPPEPVEAPPATAGIRPVLSPSASMPSYVSPPSSLLIFLAIMVFKFY

>*Potri.002G050200*-PtAGP67C
MEGLKVIKLMALLSTLLVISVNGQINTPCTMSMISSFTPCVNFITGSTSNGSPPTASCCSSLKSLMSTGMDCACLLL
TANVPVQLPINRTLAISLPGACGVPGQCKSSGTPLPAPGPFSLGPTLPPPAAAPLSPRASKAVALAPAPESEITLPL
TPASPPVQVASLPTTAGIRPVLSPPASMSSHVSPPSSLLIFLAIMVFKKFY

>*Potri.010G085400*-PtAGP68C
MAHTAMSMGLTMVLVTMLWAGAMAQSDCTNVLISMSPCLNYITGNSSTPSSQCCTQLASVVRSSPQCLCQVLNGGGS
SLGIEVNKTQAIALPGACNVQTPPISSCNGASPAASPAGTPEASSTPSGTASKTVPSTQTDGTSGGSSLDFSISLLF
FLLFAASYGSTFTVIF

>*Potri.008G155100*-PtAGP69C
MAHTAMSMDLAMVLVTMLCARAMAQSDCTSVLISMSPCLNYITGNSSTPSSQCCTQLASVVRSSPQCLCQVLNGGGS
SLGINVNQTQAIALPGACNVQTPPISSCNGASPAASPAGTSEAPSSPSGTGSKTVPSTQTDGTSGASSIEFSIPLLL
LLLFAASYGSAFTKVF

>Potri.009G092300-PtAGP11K
MMLKKAVILLSLICISIAGVSGQAPATSPTATPAPPTPTSSPPPATTPPPVSAPPPVTQSPPPATPPPVSAPPPATP
PPATPPPATPPPATPPPATPPPATPPPATPPPAVPPPAPLAAPPALVPAPAPSKPKLKSPAPSPLALSPPSPPTGAP
APSLGASSPGPAGTDMSGVEKMGSVQKMVLSLVFGSAFWLLT

>Potri.010G132500-PtAGP12K
MASVLWALILACLSFPLAITDAQTPAVSPSTPTTTTPPPTTTPTTPTQSPASAVTPPTATTPISSPSPKVAPATTPV
VPPPQPPQSSPAATPIPPPATPPPQVSPAPAPATPPPAPAPAKEVPAPAPATPPPAPVPAPAIPPPAPSPSLVPSPA
PAPGKHKMKRKHKHKHHHHAPAPAPNPPSPPAPPTVTTASEDTAPAPSPNGGNTLYHQEGRARMWARMWARTVLAFA
SLLVVTGYSF

>Potri.007G051600-PtAGP13K
MDRNGILGWTLICVLVAGVGGQAPAATPTSTPATPTTPSVPLAAPAKAPAKPTTPAPVSSPPAVTPVASSPKQTVPT
PVATPLATPPPAVTPVSSPPAPVPVSSPPEKSPPSPVPVAPPTSSPVAAPTAEVPAPTPSKKKPKKAPAPGPALLSP
PAPPTEAPGPSAESMSPGSIADDSGAGRTRCFQKIAGGLALGWGLLALIF

>Potri.005G144900-PtAGP14K
MDRNGMLGLALLCIIIAGVGGQAPAATPTSTPATPTTPAVPVAAPTKPTTPAPVSSPPAATPAASPPKQTVSVPVAA
PLATPPPSATPVSSPPTPVPVSSPPAKSPPSPEPVASPTSAPPASTPVAPPTAEVPAPSHSKKKPKKHQAPAPGPAL
LSPPAPPTEAPGPSAESVSPGPSLSDNSGAETKRCLQKMAGGLALGWGLLALIF

>Potri.008G111000-PtAGP15K
MALVLWALIVACLSFPLAITDAQTPAASPSTTSPATPAPTTTTPPPSTTPAQSPISAMTLPPATTPISPPSPKVAPA
TSPIVPPPQPLQSPPTAAPIQTPALPPPPATPPPSLPPATPPPSLPPPRVSPAPAPAPAKETPSPSPAKEVPAPAPA
PATPAPTPAPAPATPAPAPAIPPPAPSPALVLSPAPAPGKHKKKRKHKHKHKRHHHAPAPAPNPPSPPAPPTVTTVS
EDTAPAPSPNPNGGNSLYHHEGWAGMLTRTVLAIAYLLVVTGYSF

>Potri.008G195700-PtAGP49K
MASFWFLTALIVPLVIFPSPSSSSSSTQLNSKTSPYPISTSPAFLTNSPPIPPFQELSPDIAPLLPSPGGVLPSPTV
SSVPTIPSTPSPPNPDEVVASGPDSAFSPLGALLASSAAPRNLINSVIVVGFIAYRSIQLFKIKKHNSLTSASLEKL
VPLQLGHVTGVSMNIVPVLARKGFSDPGWSSNWIPAGTWF

>Potri.009G063600-PtAGP16P
MEMKKIACAILFAAASVSAVMADEVAAPAPSPASGASASLPIVGSLVGASLASFIALYLQ

>Potri.009G062700-PtAGP17P
MAQLVCTFKAMAIFFVVAMYFATVTAQDLEMEPAPAPTMDKGAAYSLGMSAAVFCSSLLLSLLAFLKH

>Potri.009G063200-PtAGP18P
MAFAAMKLFAAAVVMAMLASVAVSAQDLGELAPAPAPGKDKGAASFSLGMSGALICSSLFLSMLSLLRH

>Potri.009G063000-PtAGP19P
MAFAAMKLFAAAAVVMAMLASVAVSAQDLGELAPAPAPGMDKGAASFSLGMSGALICSSLFLSMLSLLRH

>Potri.013G057500-PtAGP20P
MEALKMRVFLAIVVVLMAVSAVQNVAAAEAPAPSPTSDATTFVPAVLASLVALAFGQLLF

>Potri.003G136600-PtAGP21P
MAGVSRVVAAVVALLVVFAIVLPIAQAQAPAPAPTSDGTSIDQGIAYVLMMVALALTYLIHAADLSHSF

>Potri.006G056000-PtAGP22P
MAAQVIIPKAFLLFLVIAAFAVVSAQDSESMAPAPAPGMEAGAGFSLPVSGAIVGFSLVVSLLGFLKH

>Potri.006G055700-PtAGP23P
MAKVSVSKAVLFTFIVAMLSVAASVSAQLAPAPAPSMNTGTGFSLPAPGVIVGFSLMVSLLAFLKL

>Potri.006G056200-PtAGP24P
MELKKISCAVLVVAASMSAALAADEISAPAPSPTSGASATLPVVGSLIGASLVSFFAYYLQ

>Potri.006G055900-PtAGP25P
MAATQVTLSRAFLLLLVIVTFAVVSAQESEMAPAPAPMDAGAGFSLPVSGAIVGFSLVVSLLGFLKH

>Potri.006G055500-PtAGP26P
MAKVRISKDIMVIYVVALFMVATNVSAQVEAPAPAPAPSMDTGAGFSLPVSSAVITFSLIISFISLLKL

>Potri.006G055800-PtAGP27P
MAATQVTISRAFLLLLVIVTFAVVSAQESEMAPAPAPMDAGAGFSLPVSGAIVGFSLVVSLLGFLKH

>Potri.016G052400-PtAGP28P
MVTQVIISKAFFLLLAIAAFAVVSAQESEMAPAPAPGMDAGAGFSLPVSGAVVGFSLALSLLGFLKR

>Potri.016G052200-PtAGP29P
MAQFSIPKALVLMLVIATFAAAVSAQDSEMAPAPAPGMDAGAGFSLPVSGAIVGFSLVVSLLGFLKH

>Potri.015G022600-PtAGP30P
MAVSNISFGVLAAIVAMICAIFMPLAHGQSSAPAPSPTSDGTAIDQGIACILMLVALVLTYLNH

>Potri.015G139200-PtAGP31P
MAALKSLAPQAALLLLLVALAVLQAQLAQSQQCVSQLNRLNVCAPFVTPGASYEHQS

>Potri.002G226300-PtAGP32P
MKLFQLCLIVALLIVFGSSPRRSHAIRGSSSAPSTSQQVFRSPFSPSPFAQRAEEFASQKRRVPAGPNPLHNKR

>Potri.019G035500-PtAGP33P
MEALKMRVFLAIMVVLMALSAVQDVAAADAPAPSPTSDATTFVPAAFASLVALAFGLLF

>Potri.014G156600-PtAGP34P
MKLFHLFLLLALLIFFSSTPRSSHAARRSFSAPSTSQQVFRSPFRASPFAERAKEFESQKRKVPTGSNPLHNKR

>Potri.014G094800-PtAGP35P
MAVCASFKAFIAVLAVVSLILAVVSPSVEAQSPAPAPAPTSDGTSIDQGIAYLLMLVALVLTYLIHPLDASSYTFF

>Potri.T142100-PtAGP36P
MADKTRSFMLTFFTVVLLLLHQHFDLTAASRPLDIHSPAIPRSGSEPPPTDVHDRWYRINRYKNLESDAFRPTTPGH
SPGVGHENPPAAP

>Potri.001G387800-PtAGP37P
MARLATLALILVLVSFSHSPEARKLLEGTLVLNRLSKGASPRGEGNPITSSASRKLTTLHLAETERFLLAAPSPGIG
N

>Potri.001G268400-PtAGP38P
MAQLASTFKAMAIFFVVAMYSATVTGQDFEMAPAPAPTMDKGAACSLGMSGAVFCSTLLLSLLALLKH

>Potri.001G268500-PtAGP39P
MEFATMKFLAAVLVMAMFASVHVSAQDFGEMAPAPAPAIGAGAASYSFGTSGALICSSLFLSMLALLRH

>Potri.001G094700-PtAGP40P
MAGVSRIAVAVVALALASAFVLPVAQAQAPAPAPTSDGTSIDQGIAYVLMLVALVLTYLIHAADLSHSF

>Potri.001G268800-PtAGP41P
MEMKKIACAILFAAASVSAVMADEVAAPAPSPTSGASVSLPVVGSLVGASLASLIALYLQ

>Potri.001G268900-PtAGP42P
MASMKLSLLASLVFVAVVCSKLAVLAYDDVLAPMPAMATGSAAGYSASVAAMGFSLMVYVFEVMLH

>Potri.001G259500-PtAGP43P
MASPSRCHLVHAMVFGLLAINAAANIYDNEEPPSPPHDHEDPPLPYNHKNSVFQPSGLLNRGTPSPP

>Potri.001G004100-PtAGP44P
MEAMKMKIFVVLMVVLMAFSTMQKAAAADAPAPSPTSDATIFVPTFLASLVALAFGLLF

>Potri.012G032000-PtAGP45P
MAVSNISFGVFAAIVAIIFAISMPLAHGQSSAPAPSPTSDGAAIDQGVACILMLVALVLTYLIH

>Potri.012G144100-PtAGP46P
MTLGRCLTKLFACLILPLLPIEAMASQHTYIPHCIPISSVPYGAPPQIPPPPLSSFNTSPFINHHDFLVFPAAEIQC
STPPTVILTTPL

>Potri.016G052300-PtAGP53P
MSKDRSLRHSFCLKPEPKLHRKIRKQLISLVSLIYLLLLLSMAAQVIVSKTFLLVLVIAAFAVASAQDSESMAPAPA
PGMDAGAAGFSSPVSGAIVGLSLIVSLLGFLKH

>*Potri.003G220900*-PtAGP54P
PTKEHWTAVWQPLSLPPCSHTSLQNLSLPLYICAPTPTHKHLPSTFLQAVVSILSLQLLSKQISFQRGIVVFLSLLN
NKKMEAMKMKVFAVLMVVLMAFSTMQKATAANAPAPSPTSDATIFVPTFLASFVALAFGFFL

>*Potri.006G056100*-PtAGP55P
MAQFGGSFVHIVMAMVVVALLHSCGMMAQSIAPTPAMDAGAGLELPVSMAIVSSSMILSLLALMLQ

>*Potri.016G052100*-PtAGP56P
MARFGGSFTRIVMAMVVVALLRICGVMAQDIAPTPAMDTGAGLELPLSMAVISSSMIFSLLALMLH

>Potri.010G244900-PtFLA1
MAIALSSFNIFFTFLLVSTFHLGFSFSALQENHSNGTYSGQINSNSVLVALLDSHYTELAELVEKALLLQTLEDAVG
KHNITIFAPKNEALERDLDPEFKRFLLEPGNLKSLQTLLLYHIVPNRINPSHNSSLQHHSTLCRDRVKLSSQESGEK
LIDSAKIIQVNAVERPDGVIHGIERLLIPRSVQQDFNNRRSLQSISAVKPEGAPEVDPRTHRLKKPAPPAKPGSAPV
LPIYDAMAPGPSLAPAPAPGPGGPHHHFNGEKQVKDFIETLLLYGGYNEMADILVNLTSLATEMGRLVSEGYVLTVL
APNDEAMAKLTTDQLSEPGAPEQIIYYHVIPEYQTEESMYNAVRRFGKISYDTLRLPHKVLAEEADGSVKFGHTENS
AYLFDPDIYTDGRISVQGIDGVLFPLEEKEKSETKKEIKSVKVAVKPQRRGRLLEVACRMLGTFGQDSHFTTCQ

>Potri.009G012200-PtFLA2
MRPQSFILALSLIFFFLHCTKTLCQSPAAAPAMAPPKTPVATSPGPVDVNKILQKAGHFTVFARLMQATTEDTELNK
ELNTTNNGITILAPTDNAFSSLKAGFLNSLSDEDKTELVKFHVLPAFISTSQFQTVSNPVRTQAGTGPRVTLNVTTT
GNFVNISSGLTNTSISGTVYTDSQLAIYQLDKVLFPLDIFTPKPPAPAPEPALGKPRKAAPDAESPTAPKDISGAPA
LLFLHNNALLLAVSCAFGAIIHS

>Potri.013G120600-PtFLA3
MATTPLSFFLLSLLSLSLNAQAQTPTAPAPTPSGPVNFTAVLVKGGQFATLIRLLNNTQTLNQIENQLNSSSEGMTI
FAPTDNAFNNLKAGALNGLNQQEQVQLLQYHTLPKFYTMSNLLLVSNPVPTQASGQDGVWGLNFTGQSNQVNVSTGL
VEVQINNALRQDSPLAVYPVDKVLLPEALFGVKPPTASPPAPSSKSNSTVAAAEPSTGKNSAGGRNVALGLVVGLGL
VCMGILS

>Potri.013G152200-PtFLA4
MASCSHWWHAPVYFIASAVLAFIAISTAMNSPSNNATRPTRPTSNYLSLNASRTLRESGFNIMATLLLISPEMFFLS
PNTTIFAIKDSSLVNTSLPPWFLKNLLQYHTSPLKLSMEDVFKKPQGSCFPTLVDRKKLAVTKIDAKERLAEINHVL
VSHPDMVLERRITIHGVLAPFSSLRSKDVYFGWESIQAPICDANSSLVSDANGPRIILEWTRIIHLLSSHRFVSFAI
GLNSVLDRILADHKNLSSVTIFAPPELEFVASSSPMLEKIVRLHILPQRATYIELAALPDKQRLRTLLPDEDLKITK
GVGVTQGLAINGVEIAAPEIFSSKEFIVHGITQAFKIAKFPNASR

>Potri.011G093500-PtFLA5
MQRLTILLSLLFLLSTSTTFTRGHNITHILGKHPSFSTFNHYLTLTHLAGEINSRNTITVCAVDNAAMSELLSKHPS
IATIKNILSLHVLLDYFGTKKLHQIREGTALAATMFQATGSAPGSTGFVNITDVKGGKVAFGPEDNEGNLDVFYVKS
LEEIPYNISVIQISKVLPSDVAAAPTPEPSAMNITDIMSAHGCKVFADTLIANPEASKTYQDSVDGGLTVFCPLDDP
FKAFFPKFKNLTASGKVSFLEFFGVPIYQSLAMLKSNNGIMNTLATDGEKKFDFTVQNDGEDVTLKTRSITAKIVGT
LIDEQPLAIYTIDKVLLPKELFKAAPTPAPAPAPEKEVADAPKSSKHKKPSSDVVPSDSPADSPDGDLADQTADDNA
SVTLYGGRLVAMLLSLCSGLLLL

>Potri.006G200300-PtFLA6
MDSHIYGVSKKTLLLFTLLCLSVSSISALPHQNKTGNSTGTGQMINSNSVLVALLDSHYTELAELVEKALLLQTLEE
AVGKHNITIFAPRNEALERQLDPEFKRFLLEPGNLKSLQTLVLFHIIPQRVGSNDWPGHKSNPSRHTTLCNDHLHLI
TKNSGKKVVGSADVTRPDDVTRPDGVIHGIERLLVPQSVQEDFNRRRNLRSISAVLPEGAPEVDPRTHRLKKPEPPV
RAGSPPVLPIYDAMAPGPSLAPAPAPGPGGPHHHFDGESQVKDFIQTLLLYGGYNEMADILVNLTSLATEMGRLVSE
GYVLTVLAPNDEAMAKLTTDQLSEPGAPEQIIYYHIIPEYQTEESMYNAVRRFGKIGYDTLRLPHKVAAQEADGSVK
FGSGDGSAYLFDPDIYTDGRISVQGIDGVLFPEVEKESTSVKKSVSSVKVATTKPRRGKLMEVACIMLGTLGQDSRF
TTCQ

>Potri.006G129200-PtFLA7
MRKQLLSPFVPFLMFFLYGSTTVAQTPSPAPSGPTNITAILEKAGQFTTLIRLMKSTQEADQINTQLNNSNQGLTVF
APPDNAFTNLKAGTLNSLSDQQKVQLVQFHIIPNFFSMSSFQTVSNPLRTQAGNSADGEFPLNVTTSGNQVNITTGV
NTATVANTIFTDGQLVVYQVDQVLLPLDLFGTAAAPAPAPSKPDKDVPAKAPAGSKEDASVDASGATIATVSVSVVL
IAAISLKL

>Potri.016G066500-PtFLA8
MDSHIYGVSEKTLFLFTLLCFSVASISALPHQNRTGNSTVTGQMINSNSVLVALLDSHYTELAELVEKALLLQTLEE
AVGKHNITIFAPKNEALERQLDPEFKRFLLEPGNLKSLQTLLLFHIIPQRVGSNDWPGHKSNPTRHTTLCNDHLHLI
TKNSGKKLVGAAVLTRPDDVTRPDGVIHGIERLLVPQSVQEDFNRRRNLRSISAVLPEGAPEVDPRTHRLKKPEPPV
RAGSPPVLPVYDAMSPGPSLAPAPAPGPGGPHHHFDGESQVKDFIQTLVHYGGYNEMADILVNLTSLATEMGRLVSE
GYVLTVLAPNDEAMAKLTTDQLSEPGAPEQIIYYHIIPEYQTEESMYNAVRRFGKIGYDTLRLPHKVVAQEADGSVK
FGSGDGSAYLFDPDIYTDGRISVQGIDGVLFPEVEKESTSVKKSVSSVKVATTTPRRGKLMEVACRMLGSLGQESHF
TTCQ

>Potri.016G088700-PtFLA9
MRKQLLSPFVPFLMFFLYSSTTFAQTPSPAPSGPTNITAILAKAGQFTTLIRLLKSTQEADQINTQLNNSNQGLTVF
APTDNSFANLKAGTLNSLSDQQKVQLVQFHILPNFLSMSNFQTVSNPLRTQAGNSADGEFPLNVTTSGNQVNITTGV
NTATVANTIYTDGQLVVYQVDQVLLPLDLFGTAPAPAPAPSKPEKDVPAKAPAGSKEDASVDSSGASIATVSFGVVL
IAAISLKL

>Potri.015G129400-PtFLA10
MVPQFLFSASFILFFLLHCPPTLAQSPAAAPAPPGPTNVTKVLEKGGQFSVFIRLLKATQEDVTLNGQLNNTNNAIT
IFAPSDNAFSSLKSGTLNSLSDQEKAELVQFHIIPQFLSSSQFQTVSNPLTTQAGSGGRLELNVTTTGNSVNITTGL
TNTSVSGTIYTDNQLAVYQVDKVLLPLDIFTPKPPTPAPAPEKPKKRSKAAASPESPADTSGAVSFTVLNNVVFFGV
CMVAAIYSL

>Potri.T130300-PtFLA11
MDSKVSSLLFIAILCLISSTSTAFNITKILAQYPEFVSFNDLLTQSGLAKEMSSRETITVLALDNSSIGGLNGRPLD
IAKRILSAHVILDYYDQIKLSKLKKASTIVTTLYQASGAADDRQGFLNISRTAEGIKFGSAVKGAPLVASLVKPVYA
QPYNISVLQVSEPIEAPGIENMAPPPPPAAVPKKAPAPTAKSPSKAPAPSKVEPSTPTESPTEGPVAADGPVADVPT
ASPLADAPTADETTAEAASSQMHAGGAVVVIGLLACMMGF

>Potri.002G223300-PtFLA12
MEFSMIIMFSSTLLFLCTPVAYAQTAASPPAPTPTPSSSPAPAPTPPYVSLTDLLSVAGPFHTFLSYLESTKVVDTF
QNQANNTDEGITIFVPKDDAFKNLKKPSLSNLTQDQVKQLILFHALPHYYALADFKNLSQVSPVSTFAGAGGYALNF
TDVSGTVHLDSGWSKTKVSSSVHSTDPVAVYQVDKVLLPEAIFGADIPPAPAPAPAPETSLAADSPSSDSTGDGSAP
GTSPPNSSYRIFGVDIWSQLVLALIGVLVLFL

>Potri.019G122600-PtFLA13
SYTAWHHTNVTKILEKAGHFTIFIRLLRSTQEENHLFSALNDSSTGLTIFAPTDSAFSELKSGTLNTLSDGDKSELV
KFHVVPTFLSTSQFQTVSNPLGTWAGTGSRLPLNVTSYPNSVNITTGLTNTSLSGTVYTDNQLAIYKIEKVLLPKDI
FASNAPAPAPVAPAPEKPTKAVPAVTVESPAAPVDISSAVWFMHNNVVGSVGIVAATVFSL

>Potri.019G120800-PtFLA14
SPAAAPAHAPAVVVTQPPAATPAQAAAPHGITNIHPRGNHLFSALNDSSTGLTIFAPTDSAFSELKSGTLNTLSDGD
KSELVKFHVIPTFLSTSQLQTVSNPLGTWARTGSRLPLNVTSYPNSVNITTGLTNTSLSGTVYTDNQLAIYKIEKVL
LPKDIFASHAPAPAPVAPAPEKPAKAVPAANVESPVAPVDISSAVWFMHNNVVGFMESVE

>Potri.019G093300-PtFLA15
MATSPLSLVLLSLFLSLSLHAQAQAPAAPAPAPSGPVNFTAVLVKGGQFVTFISLLNKTQTFNQIENQINSSSEGMT
IFAPTDNAFSNLKSGALNGLSQQQQVQLLQYHMLPKFYSLSNLLLVSNPVPTQASGQEGVWGLNFTGQSNQVNVSTG
LVEVQVNNALRQDFPLAVYPVDKVLLPDELFGVKPPSASPPAPATKGSSSGKSNSSDTAAEPSPGKNSAGGRNVALG
LIFGLGFVSMGILS

>Potri.014G168100-PtFLA16

MPRPLPLLTLAISLVLLASTTTVNAHNITRILAKHPQFSTFNHYLTVTHLAAEINRRQTITVLALDNAAMSSLISKQ
LSVYTLRNVLSLHVLVDYFGTRKLHQITNGTELTATMFQATGSAPGASGYVNITDLNGGKVAFGAEDNDGKLNAVYV
KSLEEIPYNISILQISQPLNSAEAEAPTAAPTLNVTAILSNQGCKAFSDLLIASGAHTTFEENVDGGLTVFCPTDPV
INGFMPKYKNLTAPQKVSLLLYHGIPIYQSLQMLKTSNGIMNTLATNGANKYDFTVQNDGEVVTLETKVTTATITGT
VKDEEPLVVYKINKVLLPRELFKAAPEKKAPAPKGEKDVADGPNADAPSDESDDQTADNDNGVNKMGGGRLAVVAPS
FFFGVVMFFLFD

>Potri.014G071700-PtFLA17
MATLQYSLLLSFTLSALVSTILAHNITDILSGFPEYSEFNKYLTQTKLADEINTRQTITVLALNNGAMTALAAKHPL
SVIKNALSLLVVLDYYDPTKLHQISKGTTLSTTLYQTTGNAPGNLGFVNITDLQGGKVGFGSAAPGSKLDSSYTKSV
KQVPYNISILEISQPIIAPGILTAPAPTPSSVNITALLEKAGCKTFASLLQTSGVIKTYQSAADKGLTIFAPNDEAF
KAAGVPDLSKLTNAEIVSLLQYHATATYSPFGSLKTSKDPISTLASNGAGKFDLTVTSAGDSVTLHTGIGPSRVAET
VLDSTPLVIFTVDNVLLPVELFGKAPSPAPAGEPVSAPSPSPVASSPAPASVEAPSPLAASPPAPPVETPGGAPAET
PFGSENSTADGSAAVHVSVPVQVTVFATVICSILMS

>Potri.014G162900-PtFLA18
MKFSMIIVLSSTLLFSCTPLAYAQKVASPPAPTPTPSPAPAPSPPYVNLTDLLSVAGPFHNFLNYLESTKVIDTFQN
QANNTDEGITIFVPKDDAFKNLKKASLSNLTQDQLKQLILFHALPHYYSLSDFKNLSQVSPVSTFAGAGGYALNFTD
TSGTVHLDSGWSKTKVSSSVHSTDPVAIYQVDKVLLPEAIFGTNIPPTPAPAPAPDTSPTADSPTSDDSAGAGSAPG
KSPPNSSYRINGVGIWSQLVLAIAGVLVLFL

>Potri.008G012400-PtFLA19
MAMAPSPPCIHIFFASILLLSNFHLGFSSSSSTLQENHSNGSYSGQINSNSVLVALLDSHYTELAELVEKALLLQTL
EDAVGKHNITIFAPRNEALERDLDPEFKRFLLEPGNLKSLQTLLLYHIVPNRINLSHNSSLHHHSTLCRDRIKLGSQ
SGEKLIDSAKIIQVNAVERPDGVIHGIERLLIPRSVQQDFNNRRSLQSISAVKPEGAPEVDPRTQRLKKPAPPAKPG
SAPVLPIYDAMAPGPSLAPAPAPGPGGPHHHFNGERQVKDFIETLLLYGGYNEMADILVNLTSLATEMGRLVSEGYV
LTVLAPNDEAMAKLTTDQLSEPGAPEQIIYYHVIPEYQTEESMYNAVRRFGKISYDTLRLPHKVLAQEADGSVKFGH
AENSAYLFDPDIYTDGRISVQGIDGVLFPLEEKEKSDTKTEMKSVKVAAKPQRRGKLLEVACRMLGTFGQDSHFTTC
Q

>Potri.001G320800-PtFLA20
MERLQHLLISLYLLILSINLTTTTAQSPAPAPAPPGPTNVIKILKKAGHFKTFIRLLKSTQLDSNLNSQLGNTNNGL
TIFAPSDSAFSALKTGTLRTLTDQEKVELMQFHIVPMFISSSQFDTVSSPLKTHAGSGARFQLNVTASGNSLNISTG
LTNTTISDTVYTDTHLAIYQVDKVLLPLDIFTPKPPPAPAPAPKLKAESESPDDAVSKKDISSAVSFVMHHDTVFF
TVGTVVAISFSL

>Potri.001G037800-PtFLA21
MSLKSSSLFALCFSSFLLFNTVRASNITQILSQYPDFSTFSSYLTQTQLAGEINSRQTITVLVVENGNMSPLSGKPN
GEIKNVLSGHVILDYYDVAKLQKLQNKTAMLTTLFQSSGQAKGQQGFLNVTVLGSNSVAFGSAVPGSSLSSNLVKSV
SSQPYNISVLQVSNIIVSAGTGNANSTTSPVPVGPPKTSPTPASSPNKPPSTSNPPPRPSPTTADGPAADSPAPSPP
AMDGPIAATPAADGPLADAPLDPKSDASVLTTGNNLALLALILLSAFFLA

>Potri.001G367900-PtFLA22
MQLTVLLSLLFLLSTATTITYGHNITSILGQHPSLSTFNHYLTLTHLAGEINRRTTITVCAVDNAAMSEILSKKPSI
STIKNILSLHVLLDYFGTKKLHQIRDGTALAATMFQATGSAPGSTGFVNITDVKGGKVAFGPKDNGGNLDVFYVKSV
EEIPYNISVIQISKLLPSDVAAAPTPEPSAMNITDIMSAHGCKVFADTLIANPDASKTYQDTIDGGLTVFCPLDDPF
KAFLPKFKNLTASGKESLLQFFGVPVYQSLAMLKSNNGIMNTLATNGDKKFDFTVQNDGEDVTLKTRGTTAKIVGTL
IDEQPLAIYSIDKVLLPKELFKAALTPAPAPAPEEAADAPKSSKHKKPSADDAPSDSPADSPDGDAADQTADNNASV
RLDGGRLVAMVLSLCLGLLML

>Potri.012G127900-PtFLA23
MVPQFLLSFSLILSFLLHCPPTLAQSPAAAPGPPGPTNVTKILEKGGQFSVFIRLLKATQEDVTLNGQLNNTNNAIT
IFAPSDNAFSSLKSGTLNSLNDQEKAELVQFHIIPQYLSSSQFQTVSNPLTTQAGSGGRLELNVTTTGNSVNITTGL
TNTSVSGTIYTDNQLAVYQVDKVLLPVDIFTPKPPTPAPAPEKPKKRSKAAESPDAPEDNSGAVSLTVLNDVVFFGV
GIVAAIFSL

>Potri.001G440800-PtFLA24

MAKYLFSFLLLLTTTTATSTFNPICTFSPTPSPTSATTPAPITNSSTNIALSPTFAPSPIITIPTPAPSSAPTPINN
TTTTTTTTSTTLTPTTTPSTARTPIPTNKITLTPTFAPSRAPTPTIITPILGPSPAPTPISSSAFTTTPTFGPSPAP
TPTNSSIFTATFSLPPTPPSTFTRQQDDLKFVFQEQIYNIIDAILGTGDFKNWANALGMADSTTFPISATFFIPSDN
SLSPTTTSADPDIFPYHIVPQRLSFADLQQFKTFSRLPTLLFDKSILITNNSASNFTLDGSRLTHPDIYTNAAITVH
CIDNLLDHSVYGTESGKNSSKPDAVGPPPTPASPPRPTPRTFVPSTADDEEFTVHQHGESDAACLCTEVWTVFLVLC
VALASKFQRMILVH

>*Potri.018G005100*-PtFLA25
MDFKVSSLLFIAILCLISSTSTAFNITKILAQYPEFVSFNDLLTQSGLAKEMSSRETITVLALDNSSIGGLSGRPLD
IAKRILSAHVILDYYDQIKLSKLKKASTIVTTLYQASGAADDRQGFLNISRTAEGIKFGSAVKGAPLVASLVKPVYA
QPYNISVLQVSEPIEAPGIENMAPPPPPAAVPKKAPAPTAKSPSKAPAPSKVEPSTPTESPTEGPVAADGPVADVPT
ASPLADAPMADETTAEAASSQMHAGGAVVVIGLLACMMGF

>*Potri.006G276200*-PtFLA26
MIHQYCHCYCWLKTLSNYRKLSTLLFIPYITRPLILICRLRNSFSTMNSPATHFWSTSLHAYKGLCIISFEFKHKGS
DNNKEGFGFIFLAKMDSKASSLLFIAFLCLISTSTAFNSTKILAQYPEFANFNDLLSQSGLAQEMNSRQTITVLVLD
NGSIDGLSGRPLDIAKRILSAHVILDYYDQIKLSKLQKASTIVTTLYQASGVADNRQGFLNISRTAEGIKFGSAMKG
APLVASLVKSIYSQPYNISVLQVSEPIETPGIENMAPPPPGTAAVPKKAPAPAPSTKTPPAAPPTAKTPAKSPAKS
PSKAPAPSKEGPSTPTKAPAEGPVAADGPVAAGGPVADVPAESPEADTEVAEEAPAVAPAKAASSRMHVAGATVVIG
LFACIMGF

>*Potri.012G015000*-PtFLA27
MKHHFSVFLFPAILLLLHCTQTLSQTPTAAPAKAPAAASAPPPAATSSAQASPPVMVPVQVSKGPVNVIKILQKAGG
FAVFIRLIKSTQEDIQVFSQLNDSRDGVTIFAPTDGAFSAIIKSGVLNSLSDHQKIELVQFHIIPKILTTANFQTVS
NPITTLAGSGSRFALNVITTENMVNVTSGLTNTSVSAIVYTDSQLAVYQVDKVLLPLDIFAPKPLAPAPAPPKPKKD
DGAESPLVPEDTSSAVSCIPLNSLIIFGAGMVAAVFTL

>*Potri.013G014200*-PtFLA28
MKQQYYSLFSFSFFLLFLHCTTTFAQTSPAATPAQAPAVVVAQPPAATPTQAAQPHGITNVTKILEKAGHFTIFIRL
LRSTQEENHLFSALNDSSSGVTIFAPTDSAFSELKSGTLNTLSDGDKSELVKFHVVPTFLSTSQFQTVSNPLGTWAG
TGNRLPLNVTSYPNSVNITTGLTNTSLSGTVYTDNQLAIYKIEKVLLPKDIFASKAPAPAPVAPAPEKPTKAVPAAT
VESPVAPVDTSSALMFTQNHVVGSVAIFAAAMFAL

>*Potri.019G121200*-PtFLA29
MKPQYLLSSFSILLLFLHCTNTFAQSPAAAPAQAPAVVASPPAATPTQAAAPHGITNVTIILEKAGHFTIFIRLLRS
TQEENHLFSALNDSSTGLTIFAPTDSAFSELKSGTLNTLSDGDKSELVKFHVVPTFLSTSQFQTVSNPLGTWAGTGS
RLPLNVTSYPNSVNITTGLTNTSLSGTVYTDNQLAIYKIEKVLLPKDIFASNAPAPAPVAAAPEKPTKAVPAVTVES
PAASVDISSALIFTHNLLVGSVGLLASAMFSL

>*Potri.006G174900*-PtFLA30
MARINPAISQITPTTTLTYFLLLLLATTTTITPILAITNLTALLSSFPDFSSFTSLLASTPSITSDLSNRSALTLLV
IPNSYLSSSLDFTRRLSPSSLTDLLRYHILLQYLSSSDLHQLPPAGALITTLFQTTGRASSNFGSVNITRNPVTNAI
TISSPSPFSSSNATVLSLIKTLPYNVSIISVNSLLVPYGFDLMASETRPTLGLNITKALLDGRSFFVAASLLSASGV
VEEFEADEGGAGITLFVPTDSAFSDLSETDVSLQSLPADKKADVLKFHVLHSYYPLGSLESIVNPVQPTLATEDMGA
GSFTLNISRANGSVAIDSGIVLASVTQTVFDQNPVAIFGVSKVLLPKEIFGRNPVLTSKPGNSDMGNALPPAVALSP
ESSPKMLSSAPGVREEKSGVGGLQRFSTLPLLCALVVWYCI

>*Potri.008G127500*-PtFLA31
MAATKILLSLFFLFSFLSFSSSIPTETLREAAVILSNSGYLSMSLTLPLVSNSLIPHTPSLTIFSPSDTAFTQSGQP
PLSILRLHFSPLSFPLNSLESLSLGAKIPSLFPNYSLTITSTGDDVSLNGVKIKDSPVYDDGSLVILGVDRFFDTGF
GVLNSKGYSVMASFLDLQLMVGFTDKTALTIFAPVDEVIKAFLGDLREYSSMFLKHAVPCKIMWGDLVNFDDGVVLE
TYLEGFGITVSTSGDNLMLNDQASVNFPDMYHNDWLVIHGLQSILKEPESEYSFLDDGDEF

>*Potri.019G123200*-PtFLA32
MKQQLISSFSIFLLFLHCASTFAQIPAAAPAQAPAVVVAPPPAATPTQAAAPHGITNVTKILEKAGHFTIFIRLLRS
TQEESHLFSALNDSSTGLTIFAPTDSAFSELKSGTLNTLRDGDKSELVKFHVVPTFLSTSQFQTVSNPLGTWAGTGS

RLPLNVTSYPNSVNITTGLTNTSLSGTVYTDNQLAIYKIEKVLLPKDIFTSNAPAPAPVAPAPEKPSKAVPAVTVES
PAASVDISSALIFTNNILVGSFGLLASAMFSL

>*Potri.019G120900*-PtFLA33
MKPQYLLSSFSIFLLFLHCPNTFAQSPAAAPAQAPAVVASPPAATPTQAAAPHGITNVTKILEKAGHFTIFIRLLRS
TQDENRLFSALNDSSTGLTIFAPTDSAFSELKSGTLNTLSDGDKSELVKFHVVPNSYLLPSSRPLSGTVYTDNQLAI
YKIEKVLLPKDIFASNAPAPAPVASAPEKPTKAVPAVTVESPAASVDISSALIFTHNLVVGSVGLLASAMFSL

>*Potri.004G210600*-PtFLA34
MRQQSSSLLFSLILFFLHCTKTSGQSPAAAPVMPPPTTPVKAPPTAPSQAPSAQVATSPGPVDVIKILQKAGHFTVF
VRLMQATTEDTELNKELNKTNNGITIFAPSDSAFSNLKAGFLNALSDEDKTELVKFHVLPALISSSQFQTVSNPVRT
QAGTGPRVTLNVTTTGNFVNITTGLTNTSISGTVYTDSQFAIYQIDKVLFPLDIFTPKPPAPAPAPELGKPRKAAPG
VESPTAPKDISGALTPLILHNNALLLAVSCMVAAIFS

>*Potri.019G123000*-PtFLA35
HIAQRHKHEATVNLIFYFLLFLQCTYTFALSPAAAPAQAPAVVVAPPPAATPTQAAAPHGITNVTKILEKAGHFTIF
IRLLRSIQEENHLFSALNDSSTGLTIFAPTDSAFSELKSGTLNTLSDGDKSELVKFHVIPTFLSTSQFQTVSNPLGT
WAGTGSRLPLNVTSYPNSVNITTGLTNTSLSGTVYTDNQLAIYKIEKVLLPKEIFASNAPAPAPVAPAPEKPAKAVP
AANVESPVAPVDISSAVWFMHNNVVGSVGIVVAAVFAL

>*Potri.008G128200*-PtFLA36
MATQFLMSIILLSLLISFPIYSILDATQILSNSGYVAMALILEFGSQTDLIPPSQSLTIFSPSDTAFSLSGQPSLDL
LHFHFTPRSFSLNSLKSLPPGYQIPTLFSNHSLVISSNADSQTSVNGVKINGSALYDDGFLVIFGVDNFLDPDFTVS
GSINGSTGGIRGCYVTSGDDDCSFEEASGVLKSRGYSVMASFLDLQLAKFKDHTRLTILAPVDEVVKGFMGDFSDYR
SIFLRHVVPCKISWRDLVSLDDGVVLPTYLRGFKINVTVSSTFLMFNGVQVIVPEIYSNSWLTVHGLGGSLVMQEPT
ATASNAEKIVVDFKAIKVLIAFSFLLLCTSQLNSIL

>*Potri.019G002300*-PtFLA37
MGTQNLMINKSTAKILLHLLLLSLLHQITTATLTDQELDFALLSLRSYGYTLFPNAISTSDLRLQLLNQSSNATSTS
TFTLFCPPDSLLFSVDLASTAPHYTKSLFLHVSPSRLSTSDLRNLTAASGGTYIDSLVPNHRLLITNSLAQLNGTVD
GSILVNRVRVSVPDLFLGSDIAVHGLDGILVAGFDEKVEDTSFEAATWSPANAIGSAEQNSPLAGRFPARRRKGRNH
RHNGRNGGIRRNNHRGRRINGGHRRGVGRNVSGGTRGGGVTRGAFAMYNHRL

>*Potri.018G097000*-PtFLA38
MAPINPAISHITPTTTLTYFLLLLLATTTTTTPILAITNLTALLSSFPDFSSFTSLITSIPSLTSDLSDRSALTLLV
IPNSYLSSSLDLTRRLSPSALADLLRYHILLQYLSSSDLHQVPPSGALITTLFQTTGRASSNSGSVNITRNPVTNAI
TISSPSPFSSSNATVLSLIMTLPYNVSIISVNSLLVPYGFDLMASETRPTLGLNITKALLDGHSFLVAASLLSASGV
VQEFEADEGGAGITLFVPTDSAFSDLSATAISLQSLPADKKADVLKFHVLHSYYPLGSLESIVNPVQPTLATEDMGA
GSFTLNISRVNGSVAIDSGIVQASVTQTVFDQNPVVIFGVSKVLLPKEIFGRNPVLTSKPGNTDMGNAQPPVSALSP
ENSPKMLSSAPGVREEIKSGVGGLQWLSTLHLLCVVFVCNCI

>*Potri.013G151300*-PtFLA39
MKQQYSIFSFSMLLLSLCYINTFAQSPTAAPAQAPAVVVAQPPVATPTQAAAPHGITNVTKILEKAGHFTIFIRLLR
STQEENHLFSALNDSNTGITIFAPTDSAFSELKSGTLNTLSDGDKSELVKFHVVPTFLSTSQFQTVSNPLGTWAGTG
SRLPLNVTSYPNSVNITTGLTNTSLSGTVYTDNQLAIYKIEKVLLPKDIFGSNAPAPAPVQAPAEKPTKAVPSANVE
SPVAPVDISSAVTFMHNNVVGSLVIVAAAMFACNVEGF

>*Potri.013G151400*-PtFLA40
MKQQYSISSISVFLLFLHYTNTFAQSPAAAPAQAPAVVVAQPPAATPTQAAAPHGITNVTKILEKAGHFTIFIRLLR
STQEENHLFSALNDSSSGVTIFAPTDSAFSELKSGTLNTLSDGDKSELVKFHVVPTFLSTSQFQTVSNPLGTWAGTG
SRLPLNVTSYPNSVNITTGLTNTSLSGTVYTDNQLAIYKIEKVLLPKDIFASKAPAPAPAPAREKPTKAVPAANVES
PVAPVDISSAVTFMHNNVVVGSLVIVAAAMFACHVEGF

>*Potri.019G008400*-PtFLA41
MINGFSTKKIQYFFNAIIVLKLDPVIDPVDPPGHGSDGLTRVNSENHSNGTYSGQINSNSVLVALLDSHYTELAELV
EKALLLQTLEDAVGKHNITIFAPKNEALERDLDPEFQRFLLEPGNLKSLQTLLLYHIVPNRINPSHNSSLQHNSTLC
RDRVKLSSQESGEKLIDSAKIIQVNAVERPDGVIHGIKRLLIPRSVQQDFNNRRSLQSISAVKPEGAPEVDPRTHRL

KKPALPAKPGSAPVLPIYDAMAPGPSLAPAPAPGPGGPHHHFNGEKQVKDFIETLLLYGGYNEMADILVNLTSLATE
MGRLVSEGYVLTVLAPNDEAMAKLTTDQLSEPGAPEQIIYGLNIEASKKPKKN

>*Potri.017G111600*-PtFLA42
MSTMLLFLLILLLISSSVLAASNPFSNAMEILSTSGYLSMALTLEITSKRLHLESSAATIFAPLDIAFARLGQLSVL
DLQYHISPVRLSGYYLDSLPFGTRIPTLLPNHSLIVTTSLSYFDGKLSINGISIEESALVDFGSLIIFGMSEFFNSS
LEISPNLTPAPAPSPSPVTSLGNTSQNESTGLDVDFFGQASHLLMPRGYSIMGTFLDAQLFGIKNQTRLTIFAPVDQ
AMDAYAKNVSDYSSIFRKHVVPGLFPRQDLEGFNDGTSLPTFSGGFMINLTKSGDVLVLNGVPVIFPDMYQSDWLII
HGLNQLLTPPLKEEELVGESFSELDGAEDKPDVLDFDDYVYGAP

>*Potri.019G122800*-PtFLA43
MKPQYLLSSFSIFLLFLHCPNTFAQSQAAAPAQAPAVVASPPAATLTQAAAPHGITNVTKILEKAGHFTIFIRLLRS
TQEENHLFSALNDSSPGLTIFAPTDSAFSELKSGTLNTLSDGDKSQLVKFHVVPTFLSTSQFQTVVGYHLTSQSYTN
SVNITTGLTNTSLSGTVYTDNQLAIYKIEKVLLPKDIFASNAPAPAPVAPAPEKPTKAVPAVTVESPAASVDISSAL
IFTHNLVVGSVGLLASAMFSL

>*Potri.005G079500*-PtFLA44
MESSPKLSILLILSLYIIISSTSIDGVETTTFSSNLSPQSPQPQISTSDHFHDHSFSSHTNLLAPILSHLGFTQLAM
AVPSLPADSTTTAWSGPSTLFAPSDSSLRTCFSCSIPDLLHEHIVPGLFSIDYLRKLAFGTKIETLSPGRCITVTST
SLKNDSATPSTVKVFIGGVEITHPDLFNNGVLIIHGIQGYIAPLSPFSCDFERLSSLSFPFQEGVTPHVTSTTHQQG
IGTLVQPAIMRLMLRDAMLRLRSNGFTILSLAMRVKYPELTNLVNMTVFALDDVSIFSGSHGYISSVRFHIVPNHYL
STADLERLPVGATLPTLERGQALVVTSAGGLTGFNTAVPMRINYVRVKVPDVMRNLKIVVHAVYLPFPRIHPTSAAA
FDEMMGIGGEGQNIVAAEDGACSAVFEEDGSCGTVPPMPAQVKPSVVVRSDEDHHGL

>*Potri.019G121100*-PtFLA45
MKQQSISFFIFLLFLQCTYTFAQSPAAAPAQAPAVVVAQPPAATPTQAAAPHGITNVTKILEKAGHFTIFIRLLRST
QEENHLFSALNDSSTGLTIFAPTDSAFSELKSGTLNTLSDGDKSELVKFHVIPTFLSTSQFQTVSNPLGTWAGTGSR
LPLNVTSYPNSVNITTGLTNTSLSGTVYTDNQLAIYKIEKVLLPKDIFASNAPAPAPVAPAPEKPAKAVPAANVESP
VAPVDISSAVWFMHNNVAGSVGIVAAAVFAL

>*Potri.009G012100*-PtFLA46
MYFFYSVQYRPRLCPRMQPFILLFWLLFLHACSQTFCQSPAQSPAATQTKAPVPPPPPAGPTDTIQILLKAGRFLSF
VRLMKATHVDTQLFSQLNSSTDGITMFAPNDNAFSSLVAGAVGSLNDREKLEFVQFHILPRFLSISDFQTLSNPVKT
LAGSDRKFPLTITTSDNSVTVSSGLTKTSISNTIYTDKQVAIYEVDKVLVPKDLFPPAPPAPAPARPLAEPDPVAPR
DASSALVIAWQHRVNVVLFGAGLYIAALVMDP

>*Potri.013G151500*-PtFLA47
MKQQHSLSSFSFFLLLLHCANTFAQSPAATPAQAPAAVVAQPPAATPTQAAQPHGITNVTKILEKAGHFTIFIRLLR
STQEENHLFSALNDSSSGVTIFAPTDSAFSELKSGTLNTLSDGDKSELVKFHVVPTFLSTSQFQTVSNPLGTWAGTG
SRLPLNVTSYPNSVNITTGLTNTSLSGTVYTDNQLAIYKIEKVLLPKDIFASKAPAPAPVAPAPAKPTKAVPAATVE
SPVAPVDISSALMFAHNNVVGSVGIVAAAMFAL

>*Potri.015G013300*-PtFLA48
MKQLISFSFSLVLLFLHCTQTLSQPPNAAPAKAPAAATVPPPAATSAQASPPVMVPVQVSKGPVNVIKILQKAGHFA
FFTRLIKSTQEDIQLFSQLNDSRDGVTVFAPTDGAFSAIIKSGVLNSLTDHQKIELVQFHIIPRILTTANFQTVSNP
ITTLAGSGNRFALNVITTENMVNVTTGLTNTSVSAIVYTDSQLAIYQVDKVLLPLDIFAPKPLAPAPAPPKPKKDDG
AESPMVPEDTSGSVICMVHNTLLMFGVGLVAAAIPL

>*Potri.019G121300*-PtFLA49
MKQQLISSFSIFLLFLHCASTFAQIPAAAPAQAPAVVVAPPPAATPTQAAAPHGITNVTKILEKAGHFTIFIRLLRS
TQEESHLFSALNDSSTGLTIFAPTDSAFSELKSGTLNTLRDGDKSELVKFHVVPTFLSTSQFQTVSNPLGTWAGTGS
RLPLNVTSYPNSVNITTGLTNTSLSGTVYTDNQLAIYKIEKVLLPKDIFTSNAPAPAPVAPAPEKPSKAVPAVTVES
PAASVDISSALIFTNNILVGSFGLLASAMFSL

>*Potri.019G123100*-PtFLA50
MKPQYLLSSFSIFLLFLHCTNTFAQSPAAAPAQAPAVVASPPAATPTQAAAPHGITNVTKILEKAGHFTIFIRLLRS
TQEENHLFSALNDSSTGLTIFAPTDSAFSELKSGTLNTLSDGDKSELVKFHVVPTFLSTSQFQTVSNPLGTWAGTGS

RLPLNVTSYPNSVNITTGLTNTSLSGTVYTDNQLAIYKIEKVLLPKYIFASNAPAPAPVAAAPEKPTKAVPAATVES
PAASVDISSSLIFTHNLLVGSVGLLASAMFSL

>Potri.011G117800-PtPAG1
MESQGSLCLLWALLACYLFSFSVAYNSFYVGGNDGWVINPSESYNHWAERNRFQVNDSLVFKYNKGSDSVLRVTKDD
YNSCNTKKPLKTMDSGSSVFQFDKSGPFFFISGNEDNCRKGQKLIVAVLAVRTKQTPTPAYPPATSPKAPSPEGHNP
AQAPSRSSAPIAKPPTSSHVPSVSPVSPSPIANAPSSNAPTGAPGPSPVTKTPQISPVPSKSPSPSPYAKPPAPAHS
PESLTGSPGPSPVPLKSPSPLANTPSPSYHPVASPTPARSPSPSSPTPAKPPSSSTPSPTPESSSGPSLSPRSNEAD
LAPAPAPAASWAATPSTTMVIVASLLISSAISGWP

>Potri.006G067300-PtPAG2
MAWAFGKKNHKEWLPPISYATIQALYSKHSARSTTRKNRIHLNRKNRLSMARGLNMAFLAAIAIAALVQTSVAQTTH
TVGDTTGWAIPTGDPAFYSSWAANQTFNVGEILVFNFMANAHDVAKVTKADYDACTTSSPISLVETSPARINLDASG
EHYFICNFTGHCSAGQKMMINVSAASSSPSPAPQTSSPAPQPSTPTPQTSSPAPQPSTPTPQPSPSPQPSTPAPQPS
TPAPQPSTPAPQPSTPTPASGPSPPAPTPASGSPPSPPTATPPTPAPASGSPPSPPTATPPSTVAPPNSARSLGFAG
FTTFLSIFVVFLCY

>Potri.018G129200-PtPAG3
MARGLDMAFLAAIAVAALIHGSAAQSTHTVGDTTGWAIPPTGSAFYSTWAASQNFSVDDILVFNFAANTHDVAKVTK
ADYDACTTTSPISLFATPQVRITINASGEHYFLCNFTGHCSGGQKLMINVSAASSSPSPSPAPQTSSPTPQPSTPAP
QPSTPAPQPSTPTPQSSPAPQPSTPTPASSPTPASSPSPPTPASSPSPPPTTPPSSSPPSPPTTTPPTSPPPPNSAT
SLGLAGFTTFLSIFVALCY

>Potri.018G129400-PtPAG4
MGSTLVAFVVLGAASLLLHSSKAAVYEVGDSTGWQAPSDTSFYSNWASGKTFTVGDTLTFTFSTTVHDVATVSKSDY
DNCNTASQSNVLTVGPATITLNATGNQYYFCTLSNHCTRGQKLAITVAASSTPSPPGTPPTTPSSSPPPPSSSTPSP
PPPPSASSSLVATFALVFMSIAISFMYYF

>Potri.001G398800-PtPAG5
MESRRCLCLLWALLACYSFTSSAAYNNSFDVGGKDGWVTNPSESYNHWAEKNRFQVNDSLVFKYNNGSDSVLLVTKD
DYNSCKTKKPLKTMGSGSSVFQFDKSGPYFFISGNEDNCRKGQKMTVVVLSVKPKQAPTPVSQPPAMSPKAPSPVAY
NNPSPAPSKSPSPSAEPPASSQGPSLSPISPAPISKTPSGSPLEAPGPSLVPVKSSPPSADTPTLAPSPTSNAPTGP
VPAKSPSLSVSSPYLAPSPFSDAPTGAPGPSPVAMTPHISLVPSGSPASAPGSEISPSPLTNPPAPSQSPESPSPLA
SAPVVSPIPAKSPSSSTPTPKSSYTPAHSPNSNGADLAPAPAASCVATPSTVMVIVASFLIGSVIGVWP

>Potri.017G011200-PtPAG6
MGSKRFSGSLFVMLVLGFLLGVSRGYKFYVGGKDGWATNPSERYSHWAERNRFQVNDTLFFKYKKGSDSVLIVSKDD
YNSCNTKNPIKSLTDGDSTFIFDRSGPFFFISGNADDCNKGKKLIIVVMAVRPKPLPPTPYSPITPASSPQPTSSPP
AVSPDARSPSDSAGPAQAPSTNSKSGSSGLTAGSLSVGLVLGASIGVSFILGGFLRVV

>Potri.017G012300-PtPAG7
MGSKRFSGSLFVMLVLGFLLGVSRGYKFYVGGRDGWATNPSERYSHWAERNRFQVNDTLFFKYKKGSDSVLIVSKDD
YYSCNTKNPIKSLTDGDSSFIFDRSGPFFFISGNADDCNKGKKLIIVVMAVRPKPLPPTPYSPITPASSPQPTSSPP
AVSPDARSPSDSAGPAQAPSTNSKSGSSGLTAGSLSVGLVLGASIGVSFILGGFLRVV

>Potri.011G135400-PtPAG8
MAYTTCKDNVFHILGLLCFLLLIQKNNAYPFPVGGPKGWTVPDNTSSKSYFNDWAEHHRFQRGDSILFVYDASQDSV
VQVTKEGYENCTAEKPLATFNDGHTVFKFNQSGPHYFISGNRDHCQKNEKLAVVVLADRSTNATASPPSPGSSDMVP
APTPSSEESPPAGTVDINPTPPPTGAPPNSASSMFVSFFGSMGAFFASSLILAI

>Potri.018G018200-PtPAG9
MASFQRAAVFSLVLMSLLWGSSQAKDLLVGGKTDAWKIPSSESDSLNKWAGKARFLIGDSLVWKYDGQKDSVLQVTK
EAYAACNTTNPIEEYKDGNTKVKLDKSGPFYFISGAEGHCEKGQKIVVVVLSQKHKQVGYVGSPAPSPVEFVGPAVA
RTSSASNLKGGLLVALGVLVLGLF

>Potri.001G192100-PtPAG10

MFNIGVTFGFAMMVLFQRSVAQTVYVVGDNDGWTVPQAGAQAYITWASGKNFMVGDTLTFNFTTNNHDVLRVQKESF
DACTSSNSIGDVISTGPVNITLDSTGEHYYICTIGRHCQFGQKLAITVSSRTTGASPPSTTPRPPPPRSPTATPSPS
SNNTSDGCAPTPAPSPTSSMIPESLPTIPSPPGSSSSNVLASFMMTMLAAIVGLVF

>*Potri.006G067400*-PtPAG11
MRSLIVFVVLGAVSLLLRGSEAVDHEVGDTTGWKSPSSTSFYSDWASGKTFALGDTLKFTFTTGAHDVATVSKSDYD
NCNTGSQNNLLTTGPATITLNVTGDMYFLCTIAGHCSAGQKLAITVAAGNTTSPGTSPPPPSAASSLVATFALMFVS
IAISLMYCF

>*Potri.003G047300*-PtPAG12
MAKIAVALFMMMALCGVCFGAGYNVGESDGWTIGVDYNQWASTKKFQVGDTLVFNYNTMFHNVLQVTKQDYESCNVK
SPVATFASGRDFITLDKAGHSYFVCGFPGHCQAGLKVAISVRASSSQSPDVPSPPSTPREIPPPPPPQTLSAPGQPN
FHPPPLGSPNVPLPPGFPNFGTPSGPGFPYLPPFESGASLHSSNLKAAMLSVIMTNLFAVFAY

>*Potri.014G049600*-PtPAG13
MANIASALLILVLAAPAAYAATTYTVGDSSGWSTTFGDYTTWVSGKTFTVGDSLLFKYSSTHTVAEVSKGDYDSCST
SNLGKTYTDGSSTVPLSTAGPMYFICPTSGHCSGGMKLAITVVAASGTPSTPTTPPVDDGSTTPPTTSGSPPTTPST
TVAPPPPSKSNNGATSILYNMMLGVFLVFGTTVALMGQ

>*Potri.001G419200*-PtPAG14
MANISYQNKVFHVLGLLCFLLLIQKNNAFQYQVGGGSKGWTVPDNTSSSSKSYYNDWAERTRFRIGDSLLFAYDPSQ
DSVLQVSKGDYENCTTKNPIAAFSDPKTVFTFNHSGHHYFISGNKDNCLKNEKLVVVVLADRSSNHSANTNQTTAAP
SPSLGYSDMVPAPTPSGVETPPAPAGIADINPTPAPAGVSPNSASSLFVSFIGSMGAFFASSLILSF

>*Potri.006G184100*-PtPAG15
MASHKVALLSSILVVSLFVTFTEARDIMVGGKNYSWKIPSSESDSLNKWAEASRFRVGDTLVWTYDPKKDSVLQVIK
KDYETCNTSSPLVTYKDGNTKVKLDKSGPYYFISGADGHCEQGQKLITVVMSMRSHFMGISPAPSPVEFGGPAVAPT
STGGVNLRGSLGLSFGVLTGLILL

>*Potri.006G264600*-PtPAG16
MACFQRAVACALVLMSLFVGLSQAKDLLVGGKTDAWKIPSSESDSLNKWAEKARFLVGDSLAWKYDGQKDSVLQVTK
EAYASCNTTSPIEEYKDGNTKVKLDRSGPFYFISGAEGHCEKGQKFVVLVLSQKHRHTGISPAPSPAEFEGGPAVAP
TSSAYTLRGGFLVAFGVLVLGLILM

>*Potri.013G061300*-PtPAG17
MASRCVLAIFVLIAAIVPMTTLATEYIVGDESGWTLGFDYHAWAAGKNFLVGDELVFKYPVGAHNVFKVNGTEFQNC
IIPPADRALTSGDDTIVLASPGKKWYICGVGKHCEFGQKLAITVQSLAPTPSPAPSPLYAKPDEAVKGKRPFFTLRW
W

>*Potri.002G161300*-PtPAG18
MANFRKTILVVSFLTTALCGVSMATVYQVGDSAGWTSMGQVDYQDWAANKNFHVGDTLVFNYNNQFHNVKQVTHQGF
ESCNATSPIATYTNGSDTVTLEKLGHFYFICGYPGHCQAGQKIDILVAPATSNLGPAPLSQISPSSASTLSFSNLSW
ASGVLLASCLLGFGY

>*Potri.001G268700*-PtPAG19
MANFRKTILVVSFLTTALCGVSMATVYQVGDSAGWTSMGQVDYQDWAASKNFHGGDTLVFNYNNQFHNVKQVTHQGF
ESCNATSPLATYTNGSDTVTLGKQLGHFYFICGYPGHCQAGQKIDILVAPATSNLSPAASPSSASSPYFSNLSWTLG
VLGFCLLGFAY

>*Potri.002G052500*-PtPAG20
MGSMKKTLAISCFMMALHGVSMASTVYQVGDSVGWTSMGGVDYQDWAADKNFHAGDTLVFNYNIQFHNVKQVTSQDF
ETCNATFPIATYTSGSDAINLERLGHVYFICGFRGHCLAGQKIDILISPVTSGPSPAHWPLSSRSSASSDLYFNKLY
WTLSVLVLCLSQFAY

>*Potri.001G080700*-PtPAG21

MAKLVLVYSLVVLGLALTCNAATYMVGDNSGWDISTDLDTWAQSKTFVVGDLLSFQYSSSHSLEEVKKEDFDSCNTT
NVARTFTNGNTTVPLTEPGTRYFVCGNQLHCLGGMKLQVNVEDNQANPPIGAPQAQPAGGTLTQPSSKSNNPASVIP
TSAGSVYGGRDCIVMAFLGFVATMFWVVRV

>*Potri.003G150300*-PtPAG22
MGKLVLVFSLVVLGLAVTCKAATYMVGDNSGWDISTDIDTWAQDKTFAVGDVLMFQYSSSHSVDEVKKEDFDSCNTT
NVLRTFTTGNTTVSLTNPGTRYFVCGNKLHCLGGMKLQVNVASNQADSPTGAPQTHPGGNISQPSSKSNNPASVIPT
SAGSVYGGRDSIVMAFLGFMATLSWAVQV

>*Potri.002G101300*-PtPAG23
MAGLISRSVPCAILVVLCTVVPILAKDHTVGDSSGWAIGMDYSTWTSGKTFSVGDSLVFNYGGGHTVDEVSASDYST
CTTGNAITSDSSGATTIALKTAGTHYFICGVPGHCGSGMKVAVTVAAAGSSTSPSSSGTPSSDSTTTSPAGSNVTNY
KPSSNNVPDSSLGINISPFVAIAGTCVAVFVMVF

>*Potri.013G030000*-PtPAG24
MASCRIFMIIAIVAVFVPSILATEHMVGDKKGWTLGFNYQTWAQGKAFYVGDTLVFKYTPGAHNVLSVNGTGFEECK
AADDIVPLTTGNDVITLSTPGKKWYICSVPGHCESGNQKLFITVLPQLSSPATSPFPGPTDTSPSGAAGNIASTYYG
LIAAIVGIFGMIMF

>*Potri.013G030200*-PtPAG25
MASCRIFMIIAIVAVFVPSILATEHMVGDKKGWTLGFNYQTWAQGKAFYVGDTLVFKYTPGAHNVLSVNGTGFEECK
AADDIVPLTTGNDVITLSTPGKKWYICSVPGHCESGNQKLFITVLPQLSSPATSPFPGPTDTSPSGAAGNIASTYYG
LIAAIVGIFGMIMF

>*Potri.019G037800*-PtPAG26
MASYQLIALALVTIFLPTLTMAAEHIVGDEQGWTVNFNYTTWASGKVFHVGDTLVFNYKPPHNLFKVDGAGFKDCAA
SGEPMASGNDIITLSSPGKKWYICGYGKHCSELGQKLVINVEAETPAPTPEPNAAYGLAASCYQIFAAAVAVVAMIA
A

>*Potri.T070900*-PtPAG27
MASYQLIALALVTIFLPTLTMAAEHIVGDEQGWTVNFNYTTWASGKVFHVGDTLVFNYKPPHNLFKVDGAGFKDCAA
SGEPMASGNDIITLSSPGKKWYICGYGKHCSELGQKLVINVEAETPAPTPEPNAAYGLAASCYQIFAAAVAVVAMIA
A

>*Potri.007G120200*-PtPAG28
MVRTFTSLALMAMMLRLAMAANYTVGGPNGGWDATTNLQAWAASNQFLVGDNLIFQYGLVHDVNEVSKADYDSCQIT
SPLKSYSGGTTVIPLSSPGKRYFTCATPGHCAGGMKLEIDTLATSTPPPASPLTPPPASPLTPPPASPSLPSPPTTS
TLPPASTDIPPASSPPPEIFNLSPSQSPEMTPTMSPSAPRTSPLTSPTPSPATAPSIDGFMKTPLASSASKESLQRS
LTMGISLVIMMILLAI

>*Potri.002G101200*-PtPAG29
MEYGLSHLHEISPFHILRALWNQVDLIILTPYLLIPLYNTPNDDPKSNPNTFFSSLLFSRKMAGLISRSVPCAILVV
LCTVVPILAKDHTVGDSSGWAIGMDYSTWTSGKTFSVGDSLVFNYGGGHTVDEVRASDYSTCTTGNAITSDSSGATT
IALKTAGTHYFICGVPGHCGSGMKVAVTVAAAGSSTSPSSGTPSSDGTTTSPAGSNVTNYKPSSNNVPDSSLGINIS
PFVALAGTFVAVFVVVFS

>*Potri.003G117900*-PtPAG30
MASPNKMFMIIAIVAVSVPSILATEHLVGDATGWKPGFDYGAWANGKEFHVGDTLVFKYRAGAHNVLRVNGTGFQEC
KAADDTVPLSSGNDVISLSTPGKKWYICGFAEHCESGNQKLAITVLAQLGSPSTSPSPSPTGTSPSGATSGSTVSRY
YGLIVAIVGMVMF

>*Potri.001G332200*-PtPAG31
MEYRVCLVLVFVALITKEAMAAQHVVGGSQGWEESTDFSSWASGQKFKVGDQLVFKYTSGLHSVVELGGESAYKSCG
LGTALNSMNTGNDVVKLNKPGTRYFACGTLGHCGQGMKVKITVESGTAPSTPESPSSSSSPAASSASAMHSYFATFV
LLTALVATSLLYMF

>*Potri.008G151000*-PtPAG32
MALVKRALALLMSITLAMELIHAAVYKVGDSAGWTTIGNFDYKKWSATKTFQVHDIILFKYNAQFHNVMRVTHAMYK
ACNTSAPLATYTTGNDSITIKTRGHHFFFCGVPGHCQAGQKVDINVLQSNEMAPTSSVSSSESSPPVPSAKVPGPAP
SNAMPLKALKSPSGNIGLAMAVLATFWINFA

>*Potri.017G088500*-PtPAG33
MENSGRTTIGKTVLSMAITAVTVMMIVECAAAEQLYKVGSRGWIPNYNYTDWLNQSHEHFYVGDWLLFVFDKHSYNV
LEVNETSYENCNDQGFIKNITRGGRDVVQLTEARRYYFLSSGGYCWNGMKVAINVEDFAPTPAPASSTENGSPSNIV
SRQMIILIAFCVALEWMVLFL

>*Potri.015G114300*-PtPAG34
LFLAVIFTSLRYLSVYSFEYQIGGNENWVVPPAIDTRIYVDWALGNRFQVGDTAREKDSVMKVRVEDYMKCHSRHPN
FFSSTVYHLNYPASSYFISGVSGHCEKGQRMIIIKAVEDRFLAIDLAGNGHGVV

>*Potri.010G243600*-PtPAG35
MALQRHPCIAILFAQAIIATFDATSGLRYTVGGSIWSIPPHPDFYCNWSSSHTFYIGDVLVFDFEYEFFNVIQVPKL
DYESCTALNPIRILTRSPALAILIHEGVNYYICNISNYCDLGLRFSVVVHKFYYSTGHSPAPSPLPSLPPSSPPTLS
PYPAPGPSQAGWTDVSQPSVPNNSPIAPNAGRRKGLRANSGVTVVGLACALCLGTLFVLL

>*Potri.001G187700*-PtPAG36
MKITKMASLAVLFCTCFVIATGLGNAEKVFKVGDEFGWQEPGQNSSAVYTQWATRNRFQVGDSLSFEYNNDSVIEVD
KWGYYHCDGSKPIVAFNNGHGVFKLDRPGPFYFISGTPNHCMGGQRLLIEVMGLHHHSPLTATPPAGQLAPSPQPSS
GVFVSVTLGSLSTLLMGTLIALLWCLP

>*Potri.003G050500*-PtPAG37
MTSLVVFFCICFIITVASMNGLVIAERVFKVGDVFGWQEPGQNSSSLYAQWATRNRFQVGDSLSFDYKNDSVIEVNK
WGYYHCDASKHIVAFNNGNRVFKLDKSGLFYYISGTPSHCKNGQRLLVEVMGLHHHSPPFIAAPPGYLAPSPQLSSG
VSVSGTLGSLSMALMATLIALLWSLA

>*Potri.010G089900*-PtPAG38
MALVMRAVALLTVMTLMLELIHAAVYKVGDSAGWTASGNIDYKQWSATKTFQVGDVILFEYNAQFHNVMRVTHAMYK
ACNTSAPMATYTTGNDSITIKTRRHHFFFCGVPGHCQAGQKVDINVLRSDERAQTPASSSMSSPPVPSAKVAGPASS
NALSLKALRSPFGSFGLAMAVLATFFYINLA

>*Potri.013G054500*-PtPAG39
MASSQFIAFALVTIILPTLTMAAEHIVGDDKGWTVNFNYTTWASGKVFHVGDTLVFKYQPPHNLYKVDGNGFKNCVA
SGEALTSGNDIITLGSTGKKWYICGFGKHCSELGQKLVINVEAEAPAPTPIPNAAYGLAASGYQIIVAAVAVVAGMI
VA

>*Potri.002G092800*-PtAGP57I
MKTALFITCILATLAVLANSAQNGSPPKSPAPAPSVDCSDVAVDMLDCVTYLSDGNAEKPTDSCCAGFEAVLSLDDE
CLCFALKHSADFGVAVNLTRAAALSSECGVSAPPLSRCGKPAAPSPVIEPPTNDQPSAPAPAPSNSDDNGRSAAAPV
TSDVPAQAPAKGKACAVSAPSLVLISSAVASALSLFLWI

>*Potri.003G020200*-PtAGP58I
MASKKVLSLILLCTISVSCCIWAEGASHRHASAPAPSVDCTTLVLSMADCLSFVSNDSTSKKPEGTCCSGLKTVLGT
DAECLCEAFKSSAQFGVVLNVTKALALPSACKIKAPPASNCGLTTPSPAGAPAGSAAAGPSVNGVSNELAPAPSPGS
SGSNGLFVSAGSLIVGLVVASFSSF

>*Potri.006G261800*-PtAGP59I
MASPETLAPLILAIICSIVSSCESNFPSVLFPSYSHPTPPSSYPAYPPPDNNEPVSPSPSPAYFLPEPVTPSYPAVP
ATPSYPPQTAHPMKPAPSPSAVPAYPGVPASSPPLPTPAASPVSYPPVPAPPEPYPSPPDHKGIKGAYWPSFDGFEA
SSIDTSYFTHIFYAFLLPDPVTFKLNVTPFDQQKIPGFIQNLRTRNPPVKTLLSMGGGGSDAIALIFANLSGAQETR
KVFIDSTIEVARTYGFDGLDLDWEYPANDQEMINLALLVKEWHEALVHEASASGKPRLLLTAAVYYSSQFTTFGLPR
SYPADSINKYVDWINPMCYDYHGTWENFTGPNAALYDPKSNVSTSFGIGSWIQAGVSPKKLVMGLPLYGRTWKLLDP

NVNGIGARAVGKGPEDGILDYYQVLEFNKENNAIVNFDGQTVSYYSYAGGFWVGYDDSITIDWKVQFARSRGLGGYF
FWALGQDKDWIISKQASNSWDR

>*Potri.005G167500*-PtAGP60I
MKRSLFIGCILATLALLANSAHHESSPRKSPAPSPSADCTDVAFDMLDCITYLSDGSEAAKPTASCCAGFEAVLSLD
AECLCFALKHSADFGVALNLTRAAALSSKCGVSAPPLSKCGISVPATGAPANPPSSVPEPAPPTESPPYPVIEPATN
NQPSAPAPAPSNSDDNGVSAAAPVIIEVPAQAPAKGMAYSISAPFSVLISCAVASTPLFLWV

>*Potri.001G210100*-PtAGP61I
MASRKVLSLILLCTFSISCCSQSPASAPAPSSVDCANLIFSMADCLSFVSNDSTAAKPEGKCCAGLKTVLSTKAECL
CEAFKSSARFDIVLNVTKALSLPSVCKIHAPPASNCGCQLAISPSGARAPAPGGSAPGLAVNGGGNEQAPAPSPGHS
GSIGFSISVGSLIIGFVFASFSSF

>*Potri.010G085200*-PtAGP62I
MERFVPISRMIPFLAVALAVMILPVYGQINTACTASVLATFTPCMNFLTNSTAANGTSPTAGCCGALKNLTSNGMDC
FCLIVTGSVPFSIPINRTLAISLPRACNMPGVPVQCKATGSPIPAPGPVTLGPTLSPGVSPSASPEAPVVPEPTPST
LPPVSDTTPLLTPPSSTGDTGAPTSTTGSRPVLTPPSASAPSHSLSPSLLLFAVGFVLFKCY

>*Potri.005G003500*-PtAGP63I
MMAKVASKGLFLFFLSLLTLSSSGTFVGFSYNARGITSAASLGRIVSFLELNKVSASHIRVFAADHRVLSTLSNFNV
SADLYLDDSLVEKLTKSKPSAISWLKAQIVTFLPHVYSSIIVSGNNGLSKLLSALKSIHSVLSSFHVDNEVKVSVAF
SLSFLENLNRTQEKDLRRILGFIKRTTSFVIVETSLDMDVELGMKDLFIQSMIQKVAVATSLLSPNDAPIVMIIKSL
VIPGAKEVAEFGDRVSKSLENTKIRGQVAGLYAEVSSVEDFAEKELEREHEQIFPSSRREILRNFKTTLHDDIINPP
TVFPTNPGSTPPVVTLLPDTPTPTIVTVPATNPVTVTPTNPVSTPLPFPNTTPVNVPPTNPSVNPPPPITNPVTTPA
PITVPGAQPVSPVTTYPAPAGNVPVTAPVTNPVAPPATTNAPAIPGQSWCVARSGVMDTALQSALDYACGMGGADCS
QIQQGGNCYNPNSLQNHASYAFNSYYQKNPVATSCDFGGTATTVNVNPSTGSCIYPSSSSSSSSTPSLPATTTSPA
NPATTSPANPATTSPANPTTSPPAIGVPGAPPSVLNSSTNPASSFGFNTPPALNSSASKSATLQPFIGCAILVTSFV
ARTIILDN

>*Potri.002G059600*-PtAGP70I
MGGRVIYSLMFFLLGLFLSSGSSVAEMLADENSRQADHGTKQVVVDASSISSTRKDITTPITTVPTIIPTTPTASTP
VINPNSEPDSTSPATMTPMVTPTSTASPVSPGASWCIASQSASPTALQVALDYACGYGGADCSAIQPSGSCYNPNTL
RDHASYAFNSYYQKNPVPSSCNFGGTAVTTSTNPSTGTCQFPSTSTSSSILNTTNSNGATVYGAVPSNPTPSVAARI
NETPHFMSVTFLIVFLAQLTSSLC

>*Potri.001G353400*-PtAGP71I
MASGGAVQCFTIFLLYLFLYPGHPSVAEMPILEPIQKYEVVGDENRMLISYSETAIQLDAVTGGVPIINPTNPGSGT
TPVVNPVDSPPTPIGTNPIPTTPPAGMVPPAGMVPPATMNPPPATMNPPATTNPPATTNPTSSGGAWCIASPTASET
ALQVAIDYACGYGGTDCSALQPGGSCYNPNTIRDHASYAFNSYYQKNPVPTSCVFGGTAQLTTTDPSSGNCHYASSP
TTPSISPPVNPAPTPPTPTTPPTPTMMTPTITSPGGPPTVYGVPEPVGQPSSATS

>*Potri.011G078500*-PtAGP72I
MGSGGGLQYFSIFLLYLSLYSGHPSVAGTPTLESIQKHKIVGDQENRMLISHAVSTTQLDTITGGVPVINPTTPGTT
PIVNPVDSPPASTGISPIPTTPPAGINPVDSPPAPIGISPIPTTPPAGIMTPVTPNPPASTNPTSSGGQWCIASTIA
SQTALQVAIDYACGFGGADCSAIQPGSGCYNPNTLRDHASYAFNSYYQKNPGSTSCVFGGTAQLTNTDPSNGNCHYE
SSSTTTPSTSSPVNPTPTMTTPATTSFPDGPPAVYGVAEPTGEPSSATSISCSLLLLYSTTAIVGFLVATKHL

>*Potri.005G202400*-PtAGP73I
MGGRVIYSLMFCLLGLFLSSSGSSVTKRSANENSERAGHGSKQVVVLVNSISGSQKDITTPITTVPTIIPTTSTPLI
NPNSDPDSTSPATITPMVTPTSTTTPVSPGASWCIASPSASPTALQVALDYACGYGGADCSAILPSGSCYNPNTVHD
HASYAFNSYYQKNPVPSSCNFGGTAATTSTNPSTGTCQFPSTSTSSSVLNTTNSNGATVYGAVPSNPAPSGINSLDL
LHLSCCWLKMSISDICRWCYTDLLLALLAS
