# Supplementary material for: Bioinformatic Identification and Analysis of Hydroxyproline-Rich Glycoproteins in Populus trichocarpa
Source: BMC Plant Biol. 2016 Oct 21;16:229. doi: 10.1186/s12870-016-0912-3 (PMC5073881; doi:10.1186/s12870-016-0912-3)
Supplement: Additional file 2: Figure S2. — Protein sequences encoded by the predicted EXT genes in Populus trichocarpa. The colored sequences at the N and C terminus indicate predicted signal peptides (green) and GPI anchor addition sequences (light blue) if present in the sequences. The SP3 (blue), SP4 (red), SP5 (purple), and YXY (dark red) repeats are also indicated in the sequences. The sequences typical of AGPs, specifically AP, PA, SP, TP, VP, and GP repeats, are also indicated (yellow) in the sequences. Note that green font indicates a predicted signal peptide using the sensitive mode from the SignalP website. Internal green highlights indicate the presence of a predicted signal peptide only if amino acids at the N terminus are discarded. (PDF 72 kb) [file 12870_2016_912_MOESM2_ESM.pdf]

Supplemental Figure 2

>Potri.018G050100-PtEXT1
MDLLHSVMLYFSLALLLSSSSEATDTTFSRNSALWLTYKSPPPPFHNKHKSPPPPPHKYKSPPPPHHKCKYSPPPPV
YTYRSPPPPTPMHHKSPPPSPHMFKSPPPPPYRYISPPPPPPHPPCHAYKYLSPPPPPSYKYASPPPPKHHHHHKHK
WSPYPFITYMSPPPPHHNYPDYHYSSPPPPPPIVAY

>Potri.001G019700-PtEXT2
MIYALAFCVVATSVVAKEPYYYKSPPPPLKSSPPPSPSPPPPYHYSSPPPPKKSPPPPYIYKSPPPPLKSPPPPYHY
SSPPPPKKSPPPPYHYSSPPPPKKSPPPPYVYKSPPPPSPSPPPPYHYSSPPPPKKSPPPPYVYKSPPPPSPSPPPP
YHYSSPPPPKKSPPPPYIYKSPPPPSPSPPPPYHYSSPPPPKKSPHPPYVYKSPPPPHY

>Potri.001G122100-PtEXT3
MASGALASNALLLSLYILFLALLSSAEYCPPSPPKVKSPPPPPPKAKSPPPPPKVKSPPPPKVKSPPPPKVKSPPPP
PKVKSPPPPPPMVMSPPPPIVKSPPPPMVMSPPPPIVKSPPPPMAKSPPPIDNSPPPPPMFKSPPPPPPSLPKGTCP
RDTLKLQACANVLNLLKIFVGEKEKAKCCSLIDGLVDLDAAVCLCTRIKVDLLGLIKLDVPVAVELLLNECDRKVAE
DFKCPPS

>Potri.001G259600-PtEXT4
(MASPSRRHLVHANVFGLLA)INVAANIYDNEEPPRPPHDHEDPPLPYNHKNSLFLPSGLLHRGTPPPPPRSPPPPN
KKKTPPPPPKKKKTQSPPPPAKSPPPPPPAKSPPLPSASPPTSSSAPILPPLPSKISPVTPPSKVPPSPSPASNLSP
SPPYTSPASPPRIPPIPSPSPTPNLSPSHPYTPPTSSPKVSPSPSPTPNLSPSHPYTPPTSPPRISPIPSLSLTPNL
SPLHPYTPPTSSPKVSRFPSPSPTPNSSPSHPYTPPTSPPRISPIPSPSPSPSPTPNLSPSHPYTPPTSSPKVSPPP
SPTPNLSPSHPYTPPTSPPRISPIPSPSPSPSPTPNLSPSHPYTPPTSSPKVSPSPSPTPNLSPSHPYTPPTSPPRI
SPIPSPSPSPSPTPNLSPSHPYTPPTFPPKISPIPSPPYTSPPPPPPILKSPPPPRFTLPPFFHFKSPPPPSPSPPP
PFLKSPPPPRFTLPPLFHFKSPPPPSPSPPPPILKSPPPPRLYMVNDGSGFLRRDLHII

>Potri.001G020100-PtEXT5
MENRGRMGHLSPMIHAIAICLVATSVVAYEPYYYKSPPPPSQSPPPPYHYSSPPPPKKSPPPPYHYTSPPPPKKSPP
PPYHYSSPPQPKKSPPPPYHYSSPPPPKKSPPPPYHYSSPPPPKKSPLPPYHYSSPPPPKKSPPPPYHYSSPPPPKK
SPPPQYHYTSPPPPKKSPPPPYHYTSPPPPKKSPPPPYHYTSPPPPKKSPPPPYHYTSPPPPKKSPPPPYHYSSPPP
PKKSPPPPYHYTSPPPPKKIEIVDPW

>Potri.001G019900-PtEXT6
MQPQKARVLKMENRGRMGHLSPMIHAIVICLVATSVVAYEPYYYKSPPPPSQSPPPPYHYSSPPPPKKSPPPPYHYT
SPPPPKKSPPPPYHYSSPPPPKKSPPPPYHYTSPPPPKKSPPPPYHYSSPPPPKKSPPPPYHYTSPPPPKKSPPPPY
HYSSPPPPKKSPPPPYHYSSPPPPKKSPPPPYHYSSPPPPKKSPPPPYHYTSPPPPKKSPPPPYHYSSPPPPKKSPP
PPYHYTSPPPPKKSPPPPYHYSSPPPPK

>Potri.001G260200-PtEXT7
MASPSRRHLVHAMVFGLLAINVAANIYDNEEPPRPPHDHEDPPLPYNHKNSLFLPSGLLHRGTPPPPPRSPPPPNKK
KTPPPPPKKKKTQSPPPPAKSPPPPPPAKFTLPPFFPFKSPPPPSPSPPPHILNSPPPPRFTLPPFFPIKSPPPPSP
SPPPPILKSPPPPRFTFPPFFPFKSLPPPSPSPPPHILKSPLPPWFFLPPFFFKSPPPLSPSPPSSNN

>Potri.001G020000-PtEXT8
MQTQKARVLKMESRGKMGHLSPMIHAIAICLVATSVVAYEPYYYKSPPPPSQSPPPPYHYSSPPPPKKSPPPPYHYT
SPPPPKKSPPPPYHYSSPPPPKKSPPPPYHYSSPPPPKKSPPPPYHYSSPPPPKKSPPPPYHYSYPPPPKKSPPPPY
HYTSPPPPKKSPPPPYHYSSPPPPKKSPPPPYHYSSPPPPKKSPPPPYHYSYPPPPKKSPPPPYHYTSPPPPKKSPP
PPYHYSSPPPPKKSPPPPYHYSSPPPPKKIEIVDPW

>Potri.010G001200-PtEXT9
METRHKLKLSLLALFMLLPSTKSSTMPKSRMLYQIACTMCSTCCGSTPVPSPPPPSPPPPAASPPPPATTAICPPPP
SPPPSGGGSYYYSPPPPSTYTYSSPPPPQGGVVGGTYYPPPPNYKNYPTPPPPNPIVPYFPFYYYSPPPPSMSASFKL
MASYSTSVLVGVVALVLCLF

>Potri.010G113300-PtEXT10

MLLRMSFFNSLMLLSMFLFVAAPVHGLDSRKLDETTVPAPTEEKCTPCNPSPPPPSPPPPVLCPPPPPPPELPPPTP
KKPPSGYCPPPPPAPSFIYVTGPPGNLYPIDNDFSAAGRPTVRKQDKEFSCPRI

>Potri.T091000-PtEXT11
MGGITYLLSFILIHELLLSSHISNTGKASAWEAPPSWSSPDMISSNEISYYKLKGVSRRTKPSPPPPRVNVPTHYKS
PPPRPPPPPPPPPPPPPPPRRRRPPPPPC

>Potri.013G045700-PtEXT12
MGGIAYLLSFILIHELLLSSHISNTGKASAWEAPPSWSSPDVISSNEISYYKLKGVSRRTKPSPPPPRVNVPTHYKS
PPPRPPPPPPPPPPPPPPPPPPPRRRRPPPPPPC

>Potri.003G064900-PtEXT13
MDTKSSITSISLILGLIFTFCALLVESQSQNGPSPPSPPPSPPPPFPPPPSPPPPPPPLPPPPSPPPPPPPPPPPPS
KSPPPPPRKKLQPPPPPPRDRSTGNVMRRRSHPPPPPPPPSKNHQMNSGKKIGLLFVGIAAILQIGVVGFLAYKRRQ
LLKINDRYEACSS

>Potri.006G225400-PtEXT14
MQLQEMGYLNGPLTSPRGRWFTLFFKIVIVLEMVMFARANSDADPMGEPAAGLLCISDCVTCPVICSPPPPPLLDYP
SPPSRHSPSPQHSYHSPPPQPPPPTSPPPSPPAYHSPPRSPQAPSWYPIWGTPPPPVYHFNTPNGQAPPAKGLYPYP
YYYYYSSKASSFSLHASHFFLLGVIHVVYFLF

>Potri.002G070100-PtEXT15
MGSPMASLLATLLVATISLSLPSESTATYKYSSPPPPKKSPPPPPPPYHYKSPPPPPPVHSPSPPGHPXXXXXXXXX
XXXXXXXXXXXXXXXXXXXXXXXXY

>Potri.019G015900-PtEXT16
MKVSCFMLLLMMFFAMTLQDHQPSIGYKAMALVLDRSPEMGKVKIASTDHFSTEVKVGQLGRRSRRPIPSPPPPKPN
RLVHWRWVVTPPPPMSSSPPPPSPLSLSKGA

>Potri.019G015800-PtEXT17
MKVSCFLLLLMICLAMMLQEHQPSIGYKATALVLDRSPEMGKVKIASTNHFSTEVKVGQLGRTSRRPIPSPPPPKPN
RSVHWWVVTPPPPIPSSPPPPSPLSSSKGA

>Potri.019G016000-PtEXT18
MKVSCFLLLLMMFFAMMLQDHQPSIGSKAMALVLDRSPEMGKVKIASTNHFSTEVKVGQRGRRSRRAIPSPPPPKPN
RLGRWKWVVPPVFGAPPAPTPPMPSSPPPPSPLSSSKGA

>Potri.019G017300-PtEXT19
MESLHKPVTNINGHILLLFLQQPKMGAISSLLCFFLIHFLLLSSHSSYICRAFEWEAPSRSSSDAISSNDFPNYKFR
VWRRRIPSPPPPRVNPPLHFSPPPPQAPPPPPY

>Potri.005G190100-PtEXT20
MRSSMASLTATLLLVAISLSLPSQTTANYEYSSPPPPKKSPPSPPPPYHYKSPTAPPPVHSPPPXXXXXXXXXXXXX
XXXXXXXXXXXXXXXXXXXXHSLRFIIARIGLSYIVAR

>Potri.014G124700-PtEXT21
MANRMRMLLLTLSTVITINFSLSSTISEVSKEQIPCTMCYSCDNPCQPLPSPPPPAIPECPPPPPPPSPPPPAIPEC
PPPPAPINECPACPTPPTPFPLLLMSALRSYWPGAGYSFNGPPAGYGNNPVPYFPFDNNNPPPSFSFKSVHLKLQSI
VLCIFLSLTFLCFF

>Potri.T082000-PtEXT22
MPKNMKSPMTSAVFLATTLAFVGLIAINGSQARILPDSLGLKVATLANYGRRYSPPPPSPIPSPRSKELTSNYEKYS
SPPPQSPKADPSIGQVTPSYGRTTASPPPPPKPATPKAQLEIIRSQCTDGCISMITNLERPIPSSPPSPKPAPPSSQ
ITFDLEPKVHARISPPGQNVFST

>Potri.008G129100-PtEXT23

MLLEMSFFNSFMLLSLLLFVVAPVHGLNLRKLDETTVPGPTEEKCSPCNPSPPPPSLPPVVYPSPPPPSPPPPSPVL
YPPPPPAPVLPPPTPKKPPSGYNCPPPPAPSKYDLYITGPPGELYPVDKDVNAASRHAVSLQVLIGCGLIGLLVRIG
F

>Potri.008G213600-PtEXT24
METRHKFKLSLLALLMLLSSTKSSTVLPNSRMLYQIACTMCSTCCGSSPVTSPPPPPPPPPSLATTSNCPPPPSPPA
SPGVGFYYSPPPPPPPSTYTYSSPPPPQDGVIGGTYYPPPNYKNYPTPPPPNPIVPYFPFYYYIPPPPSTSASFKLI
ASYTVLAGVALNTLLVLM

>Potri.008G125400-PtEXT25
MASCSLGRTSIQLRLSSVLAIFVLQLVFVNAGQNTRMLGGRLNDLSPPPSPKPGRLRHMIPPEIPPSTRTSPPSPPP
SHS

>Potri.001G169200-PtEXT26
METKSSITPTSLILGLILTFYSLLVESQPLNGRSSAPLMSPPPPPPPPPPPPPPPPPPPPPPPSPSLPPPSLSRSSPP
PPPPRKKLQLPPPPRHRLTVNENRRRRKPPPPMKNNNMNAGKAIGLLFVGIAAILQIGVVWFLVYKRRQL

>Potri.001G042200-PtEXT27
MKTPMTSAILLAAILAFVGLIAINGAQARILPDSFGLKVAIIAAYGRSYSSPPPSPIPSPSSKELTSNYEKRSSPPP
QSPKIDPPIGQVTTSNGRTTPSPPPPKPASPEGELKIGSPCTDGTPCNDGYISMITNFERPLPSSPPPPDPATPISH
ITFDLEPKVHARSPPGEYAYSSA

>Potri.T179500-PtEXT28
MPKNMKSPMTSAVFLATALAFVGLIAINGAQARILPDRIGLKVATLANYGGTYSPSSPSPIPSQRSKELTSNYEKYS
SPPPQSPKADPSSGQVTPSYGRTMASPPPPPKPASPKAQLEIGSQCTDGCISMITNLERPIPSSPPSPKPAPPSSQI
TFDLEPKVHARISPPGQNVFST

>Potri.T101300-PtEXT29
MCYVGKATKIFIFIVTVLVILGLVLGYELLRHRLHKSHKCSGDDDDCHPPQLTFPNPTTSGPSGLTPPSAAGIYQFS
PPPPTPPDSGTNLHPPPPPPTPPDIGTNLPPPPPPPPLLLSPPPPAVPAVTGAPPPSNNPPSSTVLVTPGPVHA

>*Potri.T139000*-PtEXT33
MKVSCFLLLLVICLAMMMLDHQPSTGYKAMALVLDCSPEMGKVKIASTDHFSTEVKVGQLGRRSRRAIPSPPPPKPN
RSVHWWVVTPPPPMPSLPPPPSPLSSSKGA

>Potri.009G108100-PtLRX1
MAEPLRVLGCFFFFSFLLSSFSNFSLALTDAEASSIARRQLLTLHENGELPDDFEYEVDVKETFANQRLRRAYIGLQ
AWKKAMYSDPFNTTGNWVGADVCAYNGVFCAPALDDSGLSVMAGVDLNGADIAGYLPAELGLLTDVALFHINSNRFC
GIIPKSFSKLTLMYEFDVSNNRFVGDFPSVVLTLPSLKYLDIRFNDFEGSLPPELFNKDLDALFLNDNRFTSTIPET
IGNSPVSVVTFANNKFTGCIPHSIGKMTNLNEVIFMGNDLGGCFPAEIGLLGNVTVFDASHNGFTGILPSSFAGLKK
VELLDLADNKLTGFVPENICRLSSLTNFTFSYNYFKGEAQACVPPSRKDTVPKQKSARTCYPVVSRPVDCSKDKCSG
GGGSSNPHPKPQPTPPTPEHKQTPSPPKSTSTPTPSSPIPAPRTPELPKPEPKLPLAPVEPISPSTPEVSLPPSLSI
SPSTPEISSPPSSSSPSTPSSDPYNPGPGGHDETPPSPKSAPSPNPFNNSPVGHNETPPSPESAPSPDPFNNSPDGH
NETPLSPESAPSPNPFNNSPDGHDETPLSPESAPSQSPESAPSPDPFNNSPDGHDETPQSSESALSPDPFNNSPDGH
DEIPPSPEPSPDPFNNSPNGHDETPTSPESAQSPDPFNNSPIGHDKTPPPSSEISIPPSPLISPPTSEKHIPPSSEF
APSPDSYNLRPVHSPPPSSQSLPPLVYSLPPPAHSPPPSIHFPPPPVHSPPPPPVYSPPLPVHSPPPSVHSPPPPMH
SPPPPPVYSPPPPVQSFPPPVHSPPPPVHSPPPPPVYSPPPPVHSPPPPVYSPPPLVQSPPPPVHSPPPPLHSPPPP
PVYSPPPPVHSPPPPVHSPPPPIQSPPPPVHSPPPPPIHSPPPPVQSLPPPPVNSPLPPVHSPPPPVHSPTSPIHSH
PPPVNSPPPPVQSPPPPVFSPPPVIVSPPPPPPEEDFILPPNLGFQYASPPPPTFPGY

>Potri.004G146400-PtLRX2
MANPLRAFGCFFFFSLLFSSFSTFSLALTDAEASYIARRQLLTLNENSELPHEFEYEVDVKITFANQRLRRAYIGLQ
AWKKAIYSDPFNTTGNWVGANVCAYNGVFCAPALDDPSLSVVAGVDLNGADIAGHLPAELGLMTDVALFHINSNRFC
GIIPESFSKLTLMYEFDVSNNRFVGDFPSVVLSWPSLKYLDVRFNDFEGSLPPELFNKELDALFLNDNRFTSTIPET
IGNSAVSVVTFANNKFTGCIPHSVGKMANLNEVIFMGNDLGGCFPAEIGLLRNVTVFDASHNGFTGILPPSFAGLKK
VELLDLADNKLTGFVPENICKLPSLTNFTFSYNYFKGEAQACVPPSRKDIVLDDTSNCLSDRPKQKSARTCYPVVSR

PVDCSKDKFHSPPPPVQSPPPPVHSPPPPVQSPPPPVHSPPPPVHSPPPPVQSPPPPVHSPPPPVHSPPPPVHSPPP
PVHSPPPPVQSPPPPVHSPPPPVHSPPPVQSPPPPVHSPPPPVHSPPPPVHSPSPPVQSPPPPPVHLPPPPVHSPPP
HVKSPPPPRPVKSSPLPIFSPPPPTVFPHPPRAFPPPPPNEDIVLPPNLGFQYASPPPPVFPGY

>Potri.006G081200-PtLRX3
MNKQLNIKRGKKGDGKGHTVQRISYDSNSLLHVTCFSSYRQASMSSPPRLFLILFLLVSTSSFLFQISSAAHEDEDH
DIDVDPNLKFENPRIRRAYIALQSWKQAIYSDPLNFTANWKGSDVCSYMGIFCAPSPQNKKVRVVAGIDLNHADIAG
YLPTELGLLTDLALFHINSNRFCGVVPNSFSKMKLLHELDLSNNRFVGKFPKVVLSLPSLKYLDLRFNEFEGSVPSQ
LFDKPLDAIFLNDNRFQFGIPENLGNSPVSVLVFANNNLGGCIPGSIGKMGKTLNEIILMNDNLTGCLPPQIGMLKE
VTVFDVSFNHLQGSLPSSIGNMKSVEQLDIAHNSFTGAIPASVCQLPNLQNFTFSFNYFTGEAPSCAAIGGVSNGTQ
NCIPGKTNQRSAKQCSSEAARPVDCSKFKCVSPRTHLPPPPPPPPSPPPPVEQQPPTYHHIPPPPSPPPPPSHYHSN
HPAPPPIEKVSPGTHYHVPPPPPSHQYQAPPPPKQSAEVPAPSYHPNSPPPPPPSCQYQAPPPPKQSAEVPAPSYHT
NSPPPPPPPTNGYTHSPPPTQPTAPVPSPVEYYPPKAASSPPPKP

>Potri.006G245600-PtLRX4
MKAKTHILPFFATLTLICLTTAQHHSFSSNAGPRDLQTIHIKQSQRQLLHYREELDIEDEYLMLPACLKFDNPRLRS
AYIALQAWKLAIISDPLNLTSNWVGSDVCNYTGVFCATSLDNSSIQTVAGIDLNHGDMAGHLVAELGLLTDIALFHV
NSNRFCGKVPKSFKKLKLLYELDLSNNRFAGRFPYVVLDLPKLKYLDLRFNEFEGDLPKELFDKDLDAIFINHNRFA
LELPDNFGNSPVSVIVLANNKFHGCFPTSLVNMSKNLNEVILMNNGLRSCLPREIGLLKKVTVFDASNNKLFGSLPD
NIGEMESLELLNVAHNMLSGNIPGSVCLLPHLKNFSYAYNFFTGEPPACLDLENFDDGRNCLRNRPKQRSTLQCKVF
LSRPVKCEAFKCHKFDPSPPPPPPPPQSPPPPPPPAPPSALPSPPPPSSMPPPPPIHPPPPFYSPPPPPNPPPPPPP
RPIYQSPPPPPPPNSPPPPPSCAEPPPPPSPPPCHEQPPPPSPPVQYLPPPPPPVYNSPPPPAPVYNGPLPPISGIP
YASPPPPPLY

>Potri.006G162300-PtLRX5
MKNKTPFPIHLSLVIAFFLGTVVCLSVAQQTPTISSYGGGLSDAEAMYINKRQLLYYKDEFGDRGERVTVDPSLVFE
NPRLKNAYMALQAWKQAIFSDPLNLTANWVGSQVCNYEGVFCSRAPDNKTIRTVAGIDLNHGDIAGYLPEELGFLVD
LALFHINSNRFCGTIPHKFKKLRLLFELDLSNNRFAGKFPQVVLKLPSLKFLDLRFNEFEGTVPKELFDKDLDAIFI
NHNRFVFDLPVNFGNSPVSVIVLANNKFHGCVPSSLGNMSNLNEIILMNNGFRSCLPAEIGLLKNLTVFDVSFNQLI
GPLPDTVGGMVSLEQLNVAHNMLSGKIPASICQLPNLENFTFSYNFFTGEPPVCLSLPDFSDRRNCLPGRPEQRSAA
QCKAFLSMPVDCSSFRCAPFVPSLPAPPPPSPPVPVLSPPPPVVIPKSPPAPPPPVYSPPPPPVYSPPPLPPVYSPP
PPPPPPPCIEPPPPSPPPPIHYSPPQPRPVHYRSPPRPSLLPPVNYHSPPPPSLPPPIPCENPPPPPPPPPPPPIIYG
SPPPPTPVYEGPLPPVTGVSYASPPPPPFY

>Potri.018G075900-PtLRX6
MKKTTHSHLHLSLLIALFLGTVVCLSAAKQAPTISSYGGLSDADAMYIKKRQLLYYKDEFGDRGERVTVDPSLVFPN
PRLRNAYIALQAWKQAIFSDPLNLTANWVGSQVCNYEGVFCAPAPDNKTIRTVAGIDLNHGDIAGYLPEELGLLVDL
ALFHINSNRFCGTVPRKFKDMRLLFELDLSNNRFAGKFPQVVLKLPSLKFLDLRFNEFEGTVPKELFDKDLDAIFIN
HNRFVFDLPENLGNSPVSVIVLANNKFHGCVPSSLGNMSNLNEIILMNNGFRSCMPAEIGLLKDLTVLDVSFNQLMG
PLPDAFGGMASLEQLNVAHNMLSGKIPASICKLPNLDNFTFSYNFFTGEPPVCLSLPDFSDRRNCLPARPLQRSAAQ
CNAFLSRPVDCSSFRCAPFVPSLPPPPPPSPPMPVPSPPPPPPVYSPPPPPPSSSPPPPIHYKPPPSPSPPPPPAVY
YHSPPPLSPPPPPVIYGSPPPPTPVYEGPLPPITGVSYASPPPPPFY

>Potri.018G151000-PtLRX7
MPPPRSFLILFLLVSTPSFLFQISSAAHEDGDHDINVDSTLKFENPRIRRAYIALQSWKQAIFSDPFNFTANWNGSD
VCSYMGVFCAPSPKNKKIRVVAGIDLNHADIAGYLPTELGLIIDLALLHINSNRFCGVVPSSFRKMKLLHELDLSNN
RLMGKFPKVVVSLPSLKYLDLRFNEFEGSVPSQLFDKPLDAIFLNDNRFRFGIPENLGNSPASVLVLANNNLGGCIP
GSIGKMSKTLNEIILMNDNLTGCLPPQIGMLKEVTVFDVSFNHLQGALPSSIGNMKSVEQLDIAHNSFTGVVPASVY
QLPNLQNFTYSFNYFTGEAPSCAAIGAVSNGTQNCIPGKMNQRSAKQCSSEAARPIDCSKFKCGGSGGASSPPPPSI
GCIIPESPPPPSPAPSYQHSQSPPPPSPTPSYHHPQSPPPPSPTPSYQHPQSPPPPSPSPTASPPPPPPPVIENIPF
PPIIGVSYASPPPPVIPYY

>Potri.018G035100-PtLRX8
MKEKTHILPFFTTLTLIGFTAAQHHSFSSNADPRDLRTTHIEQNQRQLLYYREELDVEDEYLMLPPCLKFDNPRLRS
AYIALQAWKQAIISDPLNLTSNWAGPDVCNYTGVFCATALDDSSIQTVAGIDLNHGDMAGHLVEELGLLTDIALFHI
NSNRFCGRVPKTLKKLKLLYELDLSNNRFAGGFPCVVLDLPKLKYLDLRFNEFEGDLPKELFNKDLDAIFVNHNRFA
LELPNNFGNSPVSVMVLANNKFHGCFPPSLANMSKTLNEVILMNNGLRSCLPKEIGLLKKVTVFDASNNKLVGSLPD

TIGDMESLEQLNVAHNMLSGEIHDSVCLLPHLKSFSYAYNFITGEPPACLDLEDFDDSRNCFRVRPKQRSTLQCKVF
LSRPVHCEAFNCHEFDPSPPSQPPPPPPVTSPPPPVYSPPPPPPPPLSSPPPPPSLVPPSALPSPPPPSSMPPPPPI
HFYNPPPLPAPVYNGPLPPITGISYASPPPPSIS

>Potri.T016600-PtLRX9
MKNKTPFPIHLSLVIAFFLGTVVCLSVAQQTPTISSYGGGLSDAEAMYIDKRQLLYYKDEFGDRGERVTVDPSLVFE
NPRLKNAYMALQAWKQAIFSDPLNLTANWVGSQVCNYEGVFCSRAPDNKTIRTVAGIDLNHGDIAGYLPEELGFLVD
LALFHINSNRFCGTIPHKFKKLRLLFELDLSNNRFAGKFPQVVLKLPSLKFLDLRFNEFEGTVPKELFDKDLDAIFI
NHNRFVFDLPVNFGNSPVSVIVLANNKFHGCVPSSLGNMSNLNEIILMNNGFRSCLPAEIGLLKNLTVFDVSFNQLI
GPLPDTVGGMVSLEQLNVAHNMLSGKIPASICQLPNLENFTFSYNFFTGEPPVCLSLPDFSDRRNCLPGRPEQRSAA
QCKAFLSMPVDCSSFRCAPFVPSLPAPPPPSPPVPVLSPPPPVVIPKSPPAPPPPVYSPPPPPVYSPPPLPPVYSPP
PPPPPPPCIEPPPPSPPPPIHYSPPQPRPVHYRSPPRPSLLPPVNYHSPPPPSLPPPIPCENPPPPSPPPPPPPPPP
IIYGSPPPPTPVYEGPLPPVTGVSYASPPPPPFY

>Potri.014G036700-PtLRX10
MQASTCFLVICLLLLPSISSFSVALSHSEASFIARRQPLSLEKNVNLPDYFELKIELNITFPNPRLRKAYIALQAWK
KAIRSDPFNITGNWEGLRVCDYNGVFCAPALDDPKQNVVAGIDLNHFDIVGYLPVELGLLTDIALFHINSNRFSGII
PKSFSRLTLLHELDVSNNRLVGPFPEVVISLPSLKYLDIRYNDFEGGLPPEVFEKDLDALFLNNNRFTSTIPETLGS
SPASVVVIANNKLTGCIPSSIGKMGSTLNEFVFLNNSLSGCLPSEIGKLGNATVLDVGSNSFSGVLPRCFKGLSQVE
RLDVSHNLLTGFVPEGICKLPNLVNFTFSYNYFNGEAQACSPPKRKDITMDDTSNCLPDRPKQKSPKICHPVVSKPV
DCNKAMCGGSPSIDHFLSSPPPLIPSPPPPVHSPPPPIHSPPPPVHSPPPPVHSPPPPMVSPPPPPKVVVPPNLGFS
YSSPPPPTLPGY

>Potri.010G041400-PtPERK1
MSNSVGESPPGSSSNELLPLPVSTNSTSSSGSLPPVLVGEESLVVNDDSSASLSPSTNGDSPPTPIPSSPPQATSNS
PPSPLPSSPPPLSSNSSPPLSPPLVSNSTPSNSSPPLITSPPPNSPPPSPPPPPTPAKNESPPPSVQSPPPPPAIPP
PSTGEFVSPPPSGGSKQSPPPPVESKPSLSPPNSNLSPKPSESNSTPFHSPPESGVSSSGRSSSNANAKNHNVVSNP
PRNRSNGTGSKTVMTAAAAAAAGVVFMALIVVIFLFVKRSRKRRIQPSSHYIPPKNFTVQTDKYYYGQQQPSVGFAG
PGSLSYHSPTAQSDSYGSQRGNMYHGGGPDSGVIGSSKTSFSYHELMEITNGFARQNIIGEGGFGCVFKGCMTDGKA
VAVKQLKAGSGQGEREFKAEVEIISRVHHRHLVSLVGYCISDNQRLLIYEFVPNKTLEHHLHAKDMPVLDWPKRLKI
AIGSAKGLAYLHEDCHPKIIHRDIKSSNILLDDAFETQVADFGLARLNDTAQTHVSTRVMGTFGYLAPEYASSGKLT
DRSDVFSFGVVLLELITGRKPVDASQPLGDESLVEWARPLLIHALETGEFGELVDTRLEKHYVESELFRMVETAAAC
VRHSAPKRPRMVQVVRALDSEGELPDLSNGVRFGQSAVYDSVQYSQDIRKFRGMAIGSNGSSEFDTSSGDYSGRHIS
REPPASS

>Potri.010G132900-PtPERK2
MATVSPSPNSPPSATPPPLNGTPPPSSTSSPPQASSPPPTSPPTQPNANPPDPRTPPAPSPPPQTPPPTVSNPPPAT
PSTPPPQPPPPMTSRSPPLSSSDPPSNSNSPPPLSATPPVSSPPPPPPSNPPSTAPPPPLLNPPTSSSPPSPSTTPP
QNSPPPSSTPPPQSSSPSPPQISSPPPPSSPPPQSSPPPPSLPPQSSPPPPSTPPPQSSPPSQPSSRPPQNSPPPPP
PTPPPASLPPPRRSPPPPASTPPENSPRPPISIAPRPSNVPPPPRLTPPTSPQATPVPSDSNPPALSPPKIRLAPPP
RALVSPPSPTNNTAPNSPGSSNSTGNGGIGTGGMVAIGAAIGIIMLSLVGLALWCMRRRRKEISGLNGVYVMPSSLG
SSPRSGSTFTKTHSTAPLIASGSSSDCVSLPTESSGLGNSRPLFAFEELVKATNGFSSQNLLGEGGFGSVYKGYLPD
GRDVAVKQLKIGGGQGEREFKAEVEIISRVHHRHLVSLVGYCICETRRLLVYDYVPNNTLYFHLHGVGGLALDWATR
VKIAAGAARGIAYLHEDCHPRIIHRDIKSSNILLDNNYEAKVSDFGLAKLALDSNTHVTTRVMGTFGYMAPEYASSG
KLTEKSDVFSYGVVLLELITGRKPVDASQPMGEESLVEWARPLLNHALENEELESLADPRLEKNYIESEMFRMIEAA
AACVRHSASKRPRMGQVVRAFDTLAIADLTNGMRVGESELFNSAQQSEEIRLFRRMAFGSQNYSIDCYSLDN

>Potri.017G110400-PtPERK3
MASLTPSPDSSPSLIPISISPPSPPDSTTNSPPPSTSQPDQTTDPPVPSTPSNPATPPPTPPPASPPAQPLSPPPSP
IIPSTPPPSAPPPSAPPPSPPASPPQAPPALTPPSPPAAPPPASTTSPPPPNESPPPPVSTSPPPQASPTPPPPLQA
VSPSPPPPANVPIPPSTNSPPPPTAKSPETPPAPPTVTTPAPSSQSDSPPPKTNSPPPPIPTLPSPPPSVPSTSSTP
PSISPPAPPVNSSATGSPTSPIPSIPTEKPTARATNDTNVSANTSSSGPGGSNTGGAVAIGIVVGFVALSLLVMAVW
FAQKRKRRKGENIGYTMPSPFASSQNSDSLFLKPYPPAPLVGSPSGSDFIYSPSESGVINNSRSWFTYEELVQATNG
FSAQNRLGEGGFGCVYKGVLVDGREVAVKQLKIGGSQGEREFRAEVEIISRVHHRHLVSLVGYCISEHQRLLVYDYL
PNDTLYYHLHGEGRPHMDWATRVKVAVGAARGIAYLHEDCHPRIIHRDIKSSNILLDDNFEAQVSDFGLAKIALELD
SNTHVSTRVMGTFGYMAPEYATSGKLTEKSDVYSFGVVLLELITSRKPVDASQPIGDESLVEWARPLLTTALENEDF

EALVDPELEKNYVPSEMFRMIEAAAACVRHSAAKRPRMSQVVVRALDLLDESSDLSNGMKPGQSEIFDSRQHSAQIR
MFQRLAFGNQDYSSGFFDRTQSSWRSRDPGD

>Potri.009G115200-PtPERK4
MSSPEESPVSPPPPLESSPPPAASQPPPPAVISPPPPTTPPPPSEEISPPPPAEVSPPPPTTPPPPSDEISPPPPPP
EDSGSSPQPPSSSDGNKSPPPPPKKNDNGGSRSPPPPSSSSKFHNSPPPPRPLGPSSDSSSNNSTESSSGNGDGTNL
VPIIAGVVVGVGVLLLLALLVFLCACRKKKKRHYDYYNDHSLAPKATGGPYFNAAPPQHHASNWHNESKFGEQVVNL
PPPPGGGHGTWSTPPSHGAIMSSEMSSSYSGPHGPALPPPYPSPSLALGFTKSSFSYEELAATEGFSQAKLLGQGG
FGYVHKGVLPNGKEIAVKSLKAGSGQGDREFQAEVEIISRVHHRHLVSLVGYCIAGDKKLLVYEFVPNSTLEFHLHG
KGRPTMDWPTRLKIALGSAKGLAYLHEDCHPRIIHRDIKAANILLDYSFEAMVADFGLAKLSSDNYTHVSTRVMGTF
GYLAPEYASSGKLTDKSDVFSFGVMLLELITGRLPVDLSGEMDDSLVEWATPLCAKALEDGNYDELIDPALEGNYNP
HEVACMIACAGASVSYSAKRRPKMSQIVRALEGEVSLDEGIKPGRGFIFTSASSSDFEQSPYSTDIRKFRRTALDGI
DYASSEFDHTSEYGLNPSSSSSDEMTKSQRRGP

>Potri.004G153600-PtPERK5
MSSPAPEESPLSPPPSSAPPPSLKEPPPPPLESSPPPSTSPPPVEVSPSPPPPTDSTPPSDQSSPSPPPPENSDSS
PPPPSSSKENNSPPPPTTKDNGSNIWSPPPPSSSKSRNSPPPPRSLGQSGNSSPNNTPATSSGNGDEANLVPIIA
GTVVGVGLLLLLALLVFLCTCRSKKKRSPQYNYYKDHSPAPKAAGGPYYNATPLQQPVSNGHNESKLTEQVVNLPPP
PGGGHGAWSPALPPPPPPQVAIMSSSEMSSNYSGSHSSSGLPPSHPSLTLGFTKSSFSYGELAATAGFSQANLLGQ
GGFGYVHKGVLPNGKEIAVKSLKTGSGQGDREFQAEVEIISRVHHRHLVSLVGYCIEGGQRLLVYEFVPNSTLEFHL
HGKGHPPMGWPTRLKIALGSARGLAYLHEDCHPRIIHRDIKAANILLDYSFEAMVADFGLAKLSSDNYTHVSTRIMG
TFGYLAPEYASSGKLTDKSDVFSFGVMLLELITGRRPVDLSGDMDDSLVDWARPFCAKALEDGNYDELVDPALEGNY
DLQEMACMVACAGASVSHSAKRRPKMSRIVRALEGEVSLEEGRKAGLIFSSASSSDHDQSSYSTDMRRFRRTALDSN
DYVSSEFGHTSEYGLNPSSSSSEEMSQMTKSRTGSQRRSP

>Potri.004G105200-PtPERK6
MASLTPSPDSPPSPIPISTSPPSPPSTPDSTTNSPPPSTSQPDQTIDPPLPSTPSNPATPPPQSPPPAPPAASPPAP
PPPSPIIPSTPPPFPPPPSPPASPPPAPPALTPPSPPTAPPPASTTAPPPPPPISTSPPPRASPTTPVTSPPPPQAV
SPSPPPPANDPIPPATNSPPPPTEKPPESPPALPTVPPPPPSSQSDSPPPTTNSPPPPISTLQSPPPSIPSTSSTPP
AISPPAPPVNSSVTGSPTPPFPAIPTEKPTARATNGTDVSTNTSSTGPGGLNNGGAVTIGIVAGFVALSLLVVAVWF
AQKRKRRRGENVGYTIPSPFASSQNSDSVFLKPYPPAPLVGSPSGSDFMYSPSEAGVVNNSRQWFTYEELVQATNGF
SAQNRLGEGGFGCVYKGVLVDGRDVAVKQLKIGGSQGEREFRAEVEIISRVHHRHLVSLVGYCISEHQRLLVYDYLP
NDTLYHHLHGEGRPFMDWATRVRVAAGAARGIAYLHEDCHPRIIHRDIKSSNILLDENFEAQVSDFGLAKIALELDS
NTHVSTRVMGTFGYMAPEYATSGKLTEKSDVYSYGVVLLELITGRKPVDASQPLGDESLVEWARPLLTDAIENEDFE
ALADSGLEKNYVPSEMFRMIEAAAACVRHSAAKRPRMSQVVRALDLLDESSDLSNGMKPGQSEIFDSRQHSAQIRMF
QRLAFGSQEYSSEFFDRTQSSWRSRDHGDSV

>Potri.006G242800-PtPERK7
MDNTSPPTIPQIAPFASPPSPVVVVLPPAIPPVSNPTPPPLTPVLSPPPMTIPVLSPPVASTPPPVELAPPPEASTN
PIIPVPPPTLLPEPQIPSVTSPPPLPTRPSLPQPPLPSPPLPTSPPSQQPSTSWPPPSPPKPAPPAPATPTPIMPPL
TRPSYPPAPPSLALTPPPPLPTSMVSSPPSLALTPPPPPPPPKSMVSSPPLLPLAKGSLPMSSKQFHISTGLVVACA
FGGVFLLLVLGLLFICCKDKRRRNHSTQEHYNTSKILAPTDNNKNAHVHHSEMKCFQSGDCVITVQTKTFLPSPSTS
NTRSRSINSPWTANALPHQGPPDVAISFSNGTCTYDELVVATNGFSDANLLGQGGFGYVHKGFFPCGKEIAVKQLKE
GSNQGEREFQAEVEIISRVHHKHLVSLVGYCINGSARLLVYEFVSNNTLEFHLHGTGQPVLEWETRLKIAIGSAKGL
AYLHEDCHPKIIHRDIKASNILLDHNFEAKVSDFGLAKSFSDASASSTHISTRVVGTFGYMAPEYALSGKLTDKSDV
YSYGVVLLELITGHPPISPAESVMNESLVAWARPLLTQALEDGNFEALLDPRLGTRYNNSEMASMVACAAACVHPSS
WIRPRMSQIVHALEGGMSAQDLNAGIFRPRNNTLYGSSISSSSTYQYKENMKSFNMARGSTQDGISGNTGTTSEYG
LNPSSSSSEASSR

>Potri.018G081300-PtPERK8
MSAPTPTSPPSNTTAPPSTTTPPPSTPPPTTPATPAAPPPTSPPPPPTPSAQPPASTPPPPPAASPPSSSPPSPPS
SSPPPPPSSSPSPPSSPPPPSSTTPSTPSPPSSTTPSTPSPPSSTTPSTPTSKSSPPPPTSTSTPTSGGSSGISTGV
VVGIAIGGVAILLVVSLFFICCNKRKRRRDDEAAYYVPPPGPKDDPYGGRQQYWQQNAPPPPDRVVAAMQNPPPP
PPVASRPSPPPERVAMPPPPPPPLFMSSSGGSGSNYSGTENPYPPPSPGIALGFSKSTFSYEELARATDGFTDANLL
GQGGFGYVHRGVLPNGKEVAVKQLKAGSGQGEREFQAEVEIISRVHHKHLVSLVGYCITGAHRLLVYEFVPNNTLEF
HLHGKGRPTMDWPTRLKIALGSAKGLAYLHEDCHPKIIHRDIKASNILLDFKFEAKVADFGLAKISSDVNTHVSTRV
MGTFGYLAPEYASSGKLTDKSDVFSYGVMLLELITGRRPVDSTQSFMEDSLVDWARPLLTRALEDGNFDTLVDQKLQ

NNYDQNEMARMVACAAACVRHSARRRPRTSQVVRALEGDVSLSDLNEGIRPGNSRVYGSYGSSDYDTSQYNEDMKKF
RKMALGSQEYGASSEYSGPTSEYGLYPSGSSSEGQNTREMEMGKMKKASKGFSGSS

>Potri.007G027000-PtPERK9
MSSTPDAPSPDSGSPPPPPASPPPENSTSAPPPQSDTPPPDTSSPPPPSPPPPPASPPPENSTSAPPPQSDTPPPDT
SSPPPPSPPPPPPTSDSRSPPSPPPPKHREDASPPPPPPPKHREDASPPPPPSSENSQPAPPDSSNNGGGLTSDQIK
IVVGVAVGFGIFLIALIFICAYCSRKKKRKNRHYYGENPQGGSEQFSYNYSAQQSNWQNGPPKEHVVKLSQSPGAMG
SPWLAQPPTPPMGNSDDMSSINSGPYRPPLPPPPPNIALGFNKSTFTYDELAAATGGFDQANLLGQGGFGYVHKGVL
PNGKEIAVKSLKAGTGQGEREFQAEVDIISRVHHRHLVSLVGYCIAGGQRMLVYEFVPNKTLEHHLHGKGLPVMEWP
TRLRIALGSAKGLAYLHEDCHPRIIHRDIKAANILIDNNFEAMVADFGLAKLSSDNYTHVSTRVMGTFGYLAPEYAA
SGKLTEKSDVFSYGVMLLELITGKKPVDPSSAMEDSLVDWARPLTISALETGNFIELVDPMLESKYNHQEMQRMIAC
AAASIRHSARKRPKMSQIVRALEGDVLLDVLNEGTRSTGQSPMFSSSNGSSDYDTNSYNADMKRFRQVALGSQGFGS
SEHGTSSNDSREMDPSGIHRNYQ

>Potri.005G124400-PtPERK10
MSSTPDAPSPDSGSPPPPPASSPPPENSPPPPPPQSDSPPPDASSPPPPPPPTSESPPPPPPKHSNASPPPPPNSRS
LSPPPPPPPPPPPPPPPPPPNSSNSGGSSDQMKIVVGVAVGVGIFLIAMIFICAYCSRRKKRKNMHYYGENPQGGSEQF
SYNSPQQSNWHNGLPTEHGMKLSQSPGPMGSGWPAPPPPMMNSSDMSSNYSGPYRPPLPPPSPNIALGFNKSTFTYD
ELAAATNGFDQANLLGQGGFGYVHKGVLPNGKDIAVKSLKLGSGQGEREFQAEVDIISRVHHRHLVSLVGYCIAGGQ
RMLVYEFVPNKTLEHHLHGKGLPVMDWPTRLRIALGSAKGLAYLHEDCHPRIIHRDIKAANILIDNNFEAMVADFGL
AKLSSDNYTHVSTRVMGTFGYLAPEYASSGKLTDKSDVFSYGVMLLELITGKKPVDPSSAMEDSLVDWARPLMITSL
DTGNYNELVDPMLENNYNHQEMQRMIACAAASIRHSARKRPKMSQVARALEGDVLLDDLNEGTKPGQSSVFSGSNGS
ADYDASSYNADMKKFRQVALNSQEFGSNELGTSSNESPVTGPSGIHRNSESNY

>Potri.008G189700-PtPERK11
MSNSVGNPPPGPSPSATDGEESAALVDDTATPPPNSTNVDSPQTPEPSSPPPTSKSPPPPPPPSPPPPPPPKSNHSP
PPSPPLVSNSTKSNSSPPLKISPPPNSPPPSPNPPPTPAKKESSSSSVPSPPPPAASPPPAGKFVPPPLSRDVQQSP
PPPAEFKPSLSPPISNVSPKTLDSNSNPSNSGRVPTDSRFHSPPVPGASPSDHPSSTSTDATNHNVPRTPPAPGNES
NEAGGKTIIAAAVGAAVTGLFLLTLIAAIFLVVKSRKKRVANASGHYMPPKSFTLKTDGYHYGQQQQSVRLTGPGSP
SYHLQSAPSESHGSQRGNMYNGGGPDSDVIGTGKTFFSYHELMEITSGFARQNIIGEGGFGCVYKGCMADGKVVAVK
QLKAGSGQGDREFKAEVEIISRVHHRHLVSLVGYCISDNQRLLIYEFVPNKTLENHLHGKELPVLDWPKRLKIAIGS
AKGLAYLHEDCHPKIIHRDIKSANILLDDAFEAQASLRLSMLDSLQNVADFGLARLNDTTQTHVSTRVMGTFGYLAP
EYASSGKLTDRSDVFSFGVVLLELITGRKPVDASQPLGDESLVEWARPLLIHALETGELGELVDTRLEKHYVESELF
RMVETAAACVRHLAPKRPRMMQVVRALDSGGELSDLSNGVKFGQSTAYDSGQYNQEISNFRRMALVSNGSSEFDTFS
GDYSARDTSREQPTSGDYTSSESETRAMNRTGSYAGRRFR

>Potri.008G111600-PtPERK12
MATVSPSPNSPPLAVSSPSNSTPPPPSTTSSSPPASSPPPPTSPSSQPNANPPNPRNPSTPPPPPPQPAPPALSNPPP
ATPLTPPPTTSQSPPLSGSAPNSNSPPPPSATPPVSSPPPPPPPSSNPPSISPPPPLVNPPTSSPPPPSSIPPPQSL
PPPSTPPLQSSPPPPPSSRPPQNSPPPPPPTPSQLPTNPPPPPASVSPPRRSHPPPASTPPENSPPPPASIAPLPSN
VPPPPMLTPPTATAPLPPPVPSYSTPPALSPPTIRLSPPPPSLVSPPSPTNNTAPNSPESSNSTGNGGIGIGGIVAI
GAAIGIIMLILVGLALWCMRKQRKEISGLNGVYVMPSSLGSSPRSGSTFTKTQSTAPLIASGSSSDCFSLPPESSGL
GNSGPLFAFEELVKATNGFSSQNLLGEGGFGTVYKGYLPDGRDVAVKQLKIGGGQGEREFKAEVEIISRIHHRHLVS
LVGYCISETRRLLVYDYVPNNTLHFHLHEVGRPALDWATRVKIAAGAARGLAYLHEDCHPRIIHRDIKSSNILLDIN
FEAKVSDFGLAKLALDTNTHVTTRVMGTFGYMAPEYASSGKLTDKSDVFSYGVVLLELITGRKPVDASQPVGDESLV
EWARPLLNHALENEEFESLADPRLEKNYIESEMFQMIEAAAVCVRHSATKRPRMGQVVRAFHTLANADLTNGMRVGE
SELFNSAQQSEEIRLFRRMAFGSQNYSTDFFSPDT

>Potri.003G103800-PtFH1
MSLLSRFFYRRPPDGLLEFVDRVYVFDSCFSTDVLPDGMYQIYLHEIITELYEEFPDSSFLAFNFREGEKRSQLAEI
LCQYDVTVMDYPRQYEGCPLLPLSLIQHFLRVCESWLSKGNHQNVILFHCERGSWPLLAFLLASFLIFRKLHSGEKR
TLEIVHKEAPKGFLQLLSPLNPFPSQLRYLQYVARRNIAPEWPPPERALSLDCVIFRAIPSFDAGNGCRPIIRIFGR
NLHTKGGLSTQMLFSMSKKKKSALRHYCQADCDVIKIDIQCLVQGDVVLECLHLDLDSEREVMMFRVMFNTAFIRSN
ILMLNSDNLDILWDSKERYPKGFRAEVLFGEVESISSPKAPTTILNGEEKGGLPIEAFSRVQELFSGVEWVDSSDDA
ALWLLKQLSVISDAREFSRMQNQVSSYASPVDSEDENNASSTADSSDEAFDYVSKSTAEGMKPLMSNTVESVPFSAE
SNDPQDLDLPTDPLPQVSVKGVPFSLHQQLSVAGVGTLTSPCPPQPPPPPPFARTPPPPPPPPHPPSISNQSPLSVA
APLDPYPPPHPLPPSPPPRPSTTTSNSKSPPPPPPPPPLSNVSSGDLSTASVPTSKRDIPPPPCPPPPPNFFNKDAS

LPPASHNTCLLPPPPPPPPPNVKTSPQQLPPPPPPPPPPMTLSSSSKSTASCIGGAPPPPPPLPPFGRNPANPPRP
PPPPPPPGRNPANAPSPPPPPPGPTRPTSTAPPPPPPKPPAAPPPPPGRASVPGPPPPPGAKGSNVPPPPPPSAGR
GKASLGANSHVRGRSAGGSGNAPKRASLKPLHWVKVTRAMQGSLWADSQKQENQSRAPEIDISELESLFSAASVSDG
KGSIKAGGRRGSHINKPEKVQLVDLRRAYNCEIMLTKIKIPLPDMIKAVLALDSSALDIDQVENLIKFCPTKEEMEM
LRNYTGDKEMLGKCEQFFLELMKVPRVEAKLRVFAFRITFSSQVDDLRRNLNSINDATREVKESAKLRQIMQTILTL
GNALNQGTARGSAVGFKLDSLLKLSDTRARNNKMTLMHYLCKLLAEKMPELLDFDKDLVHLEAASKIQLKTLAEEMQ
AVSKGLEKVEQELTTSVNDGAISTGFQKVLKNFLDTAESEVRSLISLYSEVGRNADSLSQYFGEDPARCPFEQVTQI
LVVFVKLFKKSREENERQADVEKKKLEKEAMKEKATTNLSARKDGVDSDKSTLGFQIQKDLLNHGSNHTTT

>Potri.011G131700-PtFH2
MLTRMNQQQVVHMKSSCFSFLVILLCATVVVSLDYRGRTEEVFLRQLVNPATGDVDKDTAELLCIICKVDLIRLKEA
GGNPNFFLPEETFSGADELSSKGWSVAKENSQRLIKVLHPQLKETLLDCIRKNSFLFHVSGDEGGADIYHSTSLNSL
FHRHAGARRNLLQSIAEAPAPAPAVGSLIPSPAPAPDLALSPVSTPNPAPSPQELFFPRLSPPPLSLSENSSGGLTS
GRSIEPDNGKNNNKTVLIAVLVTAAVTFVLAALFFLFCTAVCRRGSGAGRNDERPLLSISLSDYSVGSTHKTFGLGN
SIKEEKLGHQSLGNISNHEKRGSSFESNFYNSDAQNVSLDESLSLGVVSGAAKFSTDNKMNILVPPPPGRTGSNPFL
KPPPGRTGSNPFLKPPPGRAEPLPHEPPANLRPPPSRAGPTPSPPPPPPPPAPPAPAKSSSSVPLPPRGSPPPPPIA
PGVKPGPRPPPPPTGGSAPRPPPPMPPGPKAPRPPLGSKRPSNSASSEGAGLGDDADADAPKTKLKPFFWDKVLANP
DHSMVWHQIKSGSFQFNEEMIETLFGYAPDKNKNEHKKESSSQDPSPQYIQILDPKKAQNLSILLRALNVTIEEVCD
ALREGNELPVELLQNLLRMAPTADEELKLRLYSGELSQLGPAERFLKALVDIPFAFKRLEALLFMCILQEEVATTKE
SFETLEVACKELRNSRLFLKLLEAVLKTGNRMNDGTFRGGAQAFKLDTLLKLSDVKGIDGKTTLLHFVVQEIIRSEG
VRAARAGRESRSISSVSVKTDDLLEDISPDTEDNYSSLGLQVVSQLSSELENVKRAAVVDADSLTGSVAKLGQSVVV
TRNFLNKDMKNLEENSGFHETLKSFVQNAEVDIMSLLEEEKRIVALVKSTGDYFYGNAGKDEGLRLFIVVRDFLIIL
DKVCKEVGEAQKRSAKTRKKEASTASSPSHKHQQPSPDIRQRLFPAIAERRMGDSSSSSDDEG

>Potri.002G240200-PtFH3
MSTSTSTSTSITILLFFLLSYAPALHFSSTSSPHNRRILHQPFFPEGSIPPTEPPSSSPPSPPSSTTPQIPFSTSTP
NPPPFFPSYPSPPPPPSPTTFASFPANISSLILPQSSKPKPTSQKPLLVAISAVISALIVLSITIIVYYARRRRNRS
NFSDDKTYTGSNISNRNADTRVIGTSNNSYKLSITSTSSNFLYMDTLVNSTRLDESSDGSDRRKLESPELRPLPPLN
KENSTLKYGNGEVGYISSTTTNSRDGREEEEEEFYSPRGSLGGRDSPSGTGSGSRRVFAAGVGFDEKSSDSSSSYSS
STSASPSRSQSLSISPPLSLSSTPKSHTLLAAQSQPPPPPMMDVDNERKSPSSASSPDVSPRNVLSSASTSPRVSHR
NNVLMRSPSLSPARILNNNLSQNTPSSSPSSVSSSPGRALNDSAPFNAQSPSLSSVSTSPGNGVLEKTPPLIISFGL
DQTAQSPSLSIASTSPERGLEKSPPPSPIVSNVLGRIQMMFPSLSASKSSFSSTRISYPTSGPPPPPPPPPLPPLSI
LQPQRQWEAPSVDASSTPTDQPISKPPALIPPSRPFVLQSTTNVSPIELPPSSKTVQDAEETPKPKLKPLHWDKVRA
SSDREMVWDHLKSSSFKLNEEMIETLFVVKTPKPKATTPNSVSPTTSRENRVLDPKKAQNIAILLRALNVTIEEVCE
GLLEATKVSPAGACYAGNVDTLGTELLESLLKMAPTKEEERKLKEYKEDSPTKLGHAEKFLKAVLDVPFAFKRVDAM
LYVANFESEVEYLKKSFETLEAACEELRNSRMFFKLLEAVLKTGNRMNVGTNRGDAHAFKLDTLLKLVDVKGADGKT
TLLHFVVQEIIRTEGARLSSTNQTPNSISSEDAKWRRLGLQVVSGLSLELTHVKKAAAMDSDVLSSDVSKLSRGTEN
ISEVVRLIEKLGMVESNQKFSESMTMFMKMAEEEIIRIQAQESVALSLVKEITEYFHGNSAKEEAHPFRIFMVVRDF
LSVLDRVCKEVGMINERTIVSSALKFPVPVNPMLPVPVNPTLPQVFSGSNASKQYNSFDDESESP

>Potri.014G174700-PtFH4
MPTSFLASSFITISLFLLSYAPLHLSSSTTTNRRILHQPFFPQDSIPPTEPPSPSPPSPPSPPPPPSQIPFPTPTPN
QSPFFPSYPSPPPPPSPTAFASFPANISSLILPQSSKPKPTSQKLLVVAISAVISALIVLGIIAFYYARRRRNQTNF
SDNKTYGGSSNSNRLQVTADTRASSNSHKLRTSSTSSEFLYLGTLVNSRRLDESSNDNTNVRSDHHKLESPELRPLP
PLNRDNSRQNYGSGNFDRDGNEEEEEEEFYSPRGSSGGRESSSGSRRGFSAAAAGFDAKSIESSSSHSSSTSASPAR
SHSLSISPPVSLSPRPKSPESVTVLAAQSLPPPSPSIMDAHNERESSSSSCASSPYASPKITLNLERNVLSSASTSP
RVLNDLDRNNVIMRSPSLSPARILNSNLAQNRPSSSPSSVSTSPDRVLNDLARFNSPSLSVASSPERGFEKSLPTSP
IVSNVFDRIEMSPSLSVAKSTFSSPRISNVSDHTRMSPTLLSSPSSSPDRKSPDVSPTMTSSVLGLNARISNVFEQL
MSVPPPPPPPPPPPPMLLPRRQWESPSVNALSTPTDQPISKPPALIPPSRPFVLQSTTNVSPIELPPSSKTMEDAE
ETPKPKLKPLHWDKVRASSDREMVWDHLRSSSFKLNEEMIETLFVVNTPKPKPATPHSVSLTPNQENRVLDPKKAQN
IAILLRALNVTIEEVCEGLLEGNVDALGTELLESLLKMAPTKEEERKLKEYKDDSPTKLGHAEKFLKAVIDVPFAFK
RVDAMLYVANFESEVEYLKRSFETLEAACEELRNSRMFLKLLEAVLKTGNRMNVGTNRGDAHAFKLDTLLKLVDVKG
ADGKTTLLHFVVQEIIRTEGARLSGTNNTPNSTSSEDAKCRKLGLQVVSGLSSELGDVKKAAAMDSDVLSSDVSKLS
RGIENISEVVRLNETLGMVESCQRFSESMTRFMKMAEGELIRIQAQESVALSLVKEITEYFHGNSAKEEAHPFRIFM
VVRDFLSVLDRVCKEVGMINERTVVSSAHKFPVPVNPMLPVPVNPTLPQVFSGSNARKQCDSSDDESASP

>Potri.012G067900-PtFH5

MALFRRFFYRKPPDRLLEISERVYVFDCCFSTEVLEEDEYKVYLGGIVAQLQDHFPDASFMVFNFREGERRSQISDI
LSQYDMTVMDYPRQYEGCPMLPLEMIHHFLRSSESWLSLEGQQNVLLMHCERGGWPVLAFMLAGLLLYRKQYTGEHK
TLEMVYKQAPRELLHLLSPLNPQPSQLRYLQYISRRNFGSDWPPSDTPLQLDCLMLRSLPLFEGGKGCRPVVRVYGQ
DPSKPANRTSKLLFSTSKTKKHVRLYRQEECMLVKIDIRCRVQGDVVLECIHLDEDLVREEMMFRVMFHTAFVQANI
LMLVRDEIDFLWDAKDQFPKDFRAEVLFVDADAVVPNVTTVEANEDGNETESASPEEFFEVEEIFSNVVDGHEAKGY
GASHKVHDNMPVDVDGKEVWKEDSDLHSFEDCASDDGNHKQEGKLDSSVDAVKDIAVDDVKYKVDEKVDSDFLAVKD
ITVDDGEIKADSVVSATGTLIRKQTTEVIGDVDGELKKMEDEGDRENSATKKLESQDPPVELSADAGRQKLEQLMLP
SPRRQPTSNAKPAADSIITEQKTKHNEQEGAHGKQTKPNTIPRWVPPNRGPFSNSMHVAHPPSRYNSAPPALTFCAS
PEDSSAGGHVKISSVATGPGDIISNDFPSPTEAPPSLDPQQIALRGPPPPPLPYSNKSSFYDFQASSGGEAPPLHSQ
IADAVSFPPPPPPTSFSRQNIQMIPQHSSPPPPPPLPQLSNRQTIGMVLPPPPPPPWKSGNTPAVFTTTYSPPPPPPS
PLLPSGASTTNHGRLGIPNPPPPPPPPLSLAHTCSTPLAQSMPTHGVIPPPPPPPPSKPAQRAPPPSQPAHGAPPPPP
PPPMRGPPLPPLVSQAPPPPPMRGPPPPPPPPPPPMRGQPLPPLVSQAPPPPPPPPGRGAPPPPPPPPGRGAPPPPP
PPGRGPPPPPPPGARVPGPPASPRPPGSAPHPPPALGVKGAADARGLPSGRGRGFLRPSGMGTSATAPRRSSLKPLH
WSKVTRAIQGSLWEELQRHGESQIAPEFDVSELESLFSATVHKPADSGGKAGGRHKSVGSKTDKVHLIDLRRANNTE
IMLTKVKMPLSDMMAAVLAMDESILDVDQVENLIKFCPTKEEMELLKGYTGDKENLGKCEQYFLELMKVPRVESKLR
VFSFKIQFGSQISEFKKSLNTVNSACDEVRNSLKLKEIMKKILYLGNALNQGTARGSAIGFKLDSLLKLTDTRASNN
KMTLMHYLCKVLAAKSQALLDFHRDLVSLETASKIQLKSLAEEMQAIIKGLEKVKKELAASENDGPVSEVFRKTLKE
FISVAETEVASVTSFYAVVGRNADALALYFGEDPARCPFEQVTATLLNFVRLFRKAHEENLKQAELERKKAEKEAEM
EKARGINLTKKNME

>Potri.009G145700-PtEXT30
MARLSICLLVIFLAIVAEAATQKPKKAKKCRDKKNYPVCFKTKNLYCPPQCPRDCYVDCATCTPVCSKPSKSPPFLP
PPPHSLSPPPTRSTPPSLSPPPTTSTPPLSPPPTTSTPPLSPPPTTSTPPPTISTPPPPPATSTPPLSPPPTDFTPP
PSSTPPPATTTPPAQNPPPPPDSSESAPKRARCKNRNYATCYGQEYTCPSACPNQYCDRPGAVCQDPRFIGGDGITF
YFHGKKDRDFCIVSDSNLHINAHFIGRRNEKLTRDFTWVQSLGILFGTHKLFIGAQKTATWDDSVDRLSLALDGEPI
YLPDGEGMKWKAEISPSVTITRSSDANAVVIEAEDNFKIKAAVVPITQKDSRIHSYGIASENCFAHLDLSFKFYKLS
GDVNGVLGQTYGSNYVSRVKMGVLMPVLGGEKEFASSNIFATDCAVARFSGQHPSSNSSENFEFANLHCASGIDGRG
VVCKR

>Potri.014G115700-PtEXT31
MKDSRVSLLVFGISFICLFLCIQARRHYHAQHHKHSHLHKSSTIPEPPTPPPEPASPPPEPASPPPEPASPPPEPAS
PPPEPASPPPVPASPSPEPASPPPVPASPPPVPASPPPLPANPSGGSGNSTGVFDVRSFGAIGDGITDDTDAFKMAW
DAACNQDDSAVILVPYGFEFMIQSTIFTGPCQGGLVFQVDGTLMPPDGPESWPQKNSRRQWLVFYRINEMSLLGGGV
IDGRGEKWWDLPCKPHKGINGTTMPGPCDSPIAIRFFMSSNLTVQGLKIKNSPQFNFRFDNCKNVHVESIHITAPAL
SPNTDGIHIENTNGVEIYNSVISNGDDCVSIGSGCYDVDIRNITCGPSHGISIGSLGNHNSRACVSNITVRDSVIRV
SDNGVRIKTWQGGSGAVSGITFSNIHMDNVRNPIIIDQFYCLSKGCTNQTSALSVSDILYENIKGTYNIRSPPMHFA
CSDSVPCTNLTLSDVELLPAEGDLVLDPYCWNAYGNFRTLTIPPVSCLMEGIPRSNLNNEMDYC

>Potri.011G066900-PtEXT32
MESQKQQLTLFIFLLLAPLACLSSGLPGEYSTVSNDLHEGLTEEGITEVFKLWKEKHQKVYKHAEEAERRIGNFKRN
LKYIIEKNGKRKSGLEHKVGLNKFADLSNEEFREMYLSKVKKPITIEEKRKHRHLQTCDAPSSLDWRNKGVVTAVKD
QGDCGSCWSFSTTGAIEAINAIVTGDLISLSEQELVDCDTTNNYGCEGGDMDSAFQWVIGNGGIDTEADYPYTGVDG
TCNTAKEEKKVVSIEGYVDVDPSDSALLCATVQQPISVGMDGSALDFQLYTGGIYDGDCSGDPNDIDHAILIVGYGS
ENDEDYWIVKNSWGTEWGMEGYFYIRRNTSKPYGVCAINADASYPTKVPSPPSPPSPPPPPSPPPPPPSPPPPCPQP
SDCGDSSFCPSDETCCCILKLFSSCIIYGCCPYENAVCCAESTYCCPSDYPICDVDDGLCLRGQGDHLGVAARRRHM
ANYKFPWTKFEEIKETKQPVLQWKRSLRCNALNYME

>Potri.004G024500-PtAEH1
MNSLKFYVILLSLLTLAIITLAQEDADANYLNHFCQNATTSTINSTYRVNLNLLLSSLASNATRNNTNGFYNTSFGQ
NTDQVYGLFICRGDVSNTVCQNCVTFATKDIVQRCPIGIASIVYYDACILRYSNRNIFSKVDQSPGFTLLNTQNITT
EPQRFNNLVGAAVNDLAARAASAPPGAKKFAVNKTSFNAFQNIYSLAQCTPDLSSSDCNRCLSAAIAGLPNCCSSKI
GGRVLFPSCYIHYEITEFYDATAVAAESPPPPPPPVVIPSPPPPQKGSVSTVLIIAIVIPIAVSIALFCMCFCFLRR
ARKTRDYVPENDVGDEITTEESLQFDLSTIEAATNNCSPDNKLGEGGFGEVYKGTLPNGQQIAVKRLSRNSGQGAAE
FKNEVVLVAKLQHRNLVRLQGFCLEREEKILVYEFVSNKSLDYFLFDPERQGLLDWSRRYKIIGGIARGILYLHEDS
RLRIIHRDLKASNILLDGDMNPKISDFGLARIFVVDQTQASTNRIVGTYGYMSPEYAMHGRFSVKSDVYSFGVLILE
IITGKKNSSFYQTGGAPDLVSYVWNHWRDGTPLEVLDPTLTDTYSRNEVIRCIHIGLLCVQEDPAIRPAMATIVLTL
NSYLVTLPSPQEPAFFFRSTITDEVNISSKEFLLDQSKRKSIAYSVDEVSITEVYPR

>Potri.004G024800-PtAEH2
MNSLKFYIILLSFLTLAIITLAQEDANHLYHNCQNATTSTINSTYRVNLNLLLSSLASNATRNNTNGFYNTSFGQNT
DQVYGLFICRGDVSNTVCQNCVTFATKDIVQRCPIGIASIVFYDACHLRYSNRNIFSKVNQSPAYYKWNLQNITTEP
QRFNNLVGAAVNDLAARAASAPPGAKKFSVNKTSFDAFQKIYSLAQCTPDLSSSDCNRCLSAAIARLPICCSSKIGG
RVLFPSCYIHYEITEFYNATAVAAESPPPPPPPVALPSPPPPRSATIPEEKGGVSTVLIIAIVIPIAVSIALLSMCF
CFLRRARKTRDYVPENDVGDEITTEESLQFDLSTIEAATNNFSADNKLGEGGFGEVYRGTLPNGHQIAVKRLSKKSG
QGAAEFKNEVVLVAKLQHRNLVRVQGFCLEGEEKILVYEFVSNKSLDYFLFDHEMQGLLDWSRRYKIIGGIARGILY
LHEDSRLRIIHRDLKASNILLDGDMNPKISDFGLARIFVVDQTQASTNRIVGTYGYMSPEYAMHGHFSVKSDVYSFG
VLILEIITGKKNSSFYQTGGAADLVSYVWKHWRDGTPLEVLDPTLTDTYSRNEVIRCIHIGLLCVQEDPAIRPAMAT
IILTLNSYSVTLPSPQEPAFFFHSTITDEVNSSSKEFLLEHSKSKSVAYSVDEDSITEVYPR

>Potri.003G082300-PtAEH3
MHSWVIGPQTNQRNLQFFCPCNLYINLGTPPLMHLHRNVSLLFSSLKPQFISHPILIRVMAFSRAMLMLTLMLALIA
TSIAQDSISLPPIMALTSVSTPPSSSAAPPSNTPTPPPAMTPPPVSSPPPMMSPPPSGTPPMSPGSPSSPPSPKAPE
APAPAVSQPGNGAFVHGNRMALSALLGGVAFLFV

>Potri.003G184500-PtAEH4
MPKNMKSPMTSAVFLATALAFVGLIAINGAQARILPDRIGLKVATLANYGGTYSPPPPSPIPSPRSKELTSNYEKYS
SPPPQSPKADPSIGQVTPSYGRTTASPPPPPKPATPKAQLEIIRSQCTDGCISMITNLERPIPSSPPSPKPAPPSSQ
ITFDLEPKVHARISPPGQNVFST
