# Supplementary material for: Bioinformatic Identification and Analysis of Hydroxyproline-Rich Glycoproteins in Populus trichocarpa
Source: BMC Plant Biol. 2016 Oct 21;16:229. doi: 10.1186/s12870-016-0912-3 (PMC5073881; doi:10.1186/s12870-016-0912-3)
Supplement: Additional file 3: Figure S3. — Protein sequences encoded by the potential chimeric EXT genes in Populus trichocarpa. The colored sequences at the N and C terminus indicate the predicted signal peptides (green) and GPI anchor addition sequences (light blue) if present in the sequences. The SP3 (blue), SP4 (red), SP5 (purple), and YXY (dark red) repeats are also indicated in the sequences. The sequences typical of AGPs, specifically AP, PA, SP, TP, VP, and GP repeats, are also indicated (yellow) in the sequences. (PDF 23 kb) [file 12870_2016_912_MOESM3_ESM.pdf]

Supplemental Figure 3

>Potri.010G192500
DNATLPMPLKSKEKPPKPLVGTLRVALKDLPDPDDSTRIRSFELTRPSGRPQGKIRIKLGVRERPFSPPPPPQPVYR
ITSPQSYYYSGLPDYPLPPSSSPPPPPVPHICSYHDAYSPSPYYSGYYSSAPPPPCHQGPFLIGQWVVMAAGPPHRL
LTVLKRHKGGKRMGFGTGLAVGAVAGALGGRALEEGLKYEEEKIAEKVENDLAVRDDYSDYRSYY

>Potri.017G129800
MENPRLSLLSSSSPKLVMGYPTSLKNPTTPKFSISTTRPSLPFPHRTSKTVTHTSRISISALSQSHGPRRTSKNGSE
YFASISSLSGQQTASVGVNPQSVSPPPSQIGSPLFWVGVGVALSAIFSWVATRLKNYAMQQAFKSLTEQMNAQNNQF
NPAFSARSPFPFSPPPASQPATSPFQTASQPAVTVDIPATKVEAAPETDARKEKETDTLEEREIKEEPRKFAFVDVS
PEETSLNTPFSSVEDVIDTSSSKDVQFAKEASQNGATFKQGPSASEPSEGSQSSQKAGSLSVEALEKMMDDPTVQKM
VYPYLPEEMRNPTTFKWMLQNPQYRQQLEEMLNNMSGSSEWDSRMVDSLKNFDLSSPEVKQQFDQIGLTPEEVISKI
MANPDVALAFQNPRVQQAIMECSQNPLSIAKYQNDKEVMDVFNKISEILG

>Potri.009G158600
MAEVINMPVDSLDHRGGNGGGGGRDRKDKPPTDDPQSSPPPPPTQRRRERDSRERREDFDRPPNRRGGDYYDRNRS
PPPPQREREREYKRRSSVSPPPPVPYRDRRHSPPQRRSPPYKRSRREDGGYDARRGSPRGGFGGGDRRFGYDYGGGY
DREMGGRPGYGEERPHGRYMGRGGGYQGGPPDWELGRGGYGNTSNVVPAQREGLMSYKQFIQELEDDILPSEAERRY
QEYKSEYISTQKRVFFEAHKDEEWLKDKYHPTNLVAVIERRNELARKVAKDFLLDLQSGTLDLGPGVNAISSNKSGQ
ASDPNSDDDAETGGKRRRHGRPPAKDTDLSAAPKAHPVSSEPKRIQVDVEQAQTLVRKLDSEKGVEENILSGSENDK
MSREKSHGSSTGPVIIIRGLTSVKGLEGVELLDTLITYLWRVHGLDYYGMIETNEAKGLRHVRAEGKSSDTSNSGTE
WEKKLDSHWQERLRGQDPLEKMTAKEKIDATAVEGLDPYVRKIRDEKYGWKYGCGAKGCTKLFHAAEFVHKHLKLKH
LELVMELTAKVREELYYQNYMNDSDAPGGTPVMQQSMPKDKPQRRRLGPLKDERGNRRERDNRANGNERFDRSENPQ
SGDFQSNDGPDGGNRDEAMFDTFGGQGIHVPSPFPSDIAPPPVLMPVPGAGPLGPFVPAPPEVAMRMFRDQGGPPPF
EGGGRNARPGPQLGGPAPILLSPAFRQDPRRIRSYQDLDAPEDEVTVIDYRSL

>Potri.004G024300
MNSLKFYVILLSLLTLAILTLAQENYLYHYCQNASTSTLNSTYRVNLNLLLSSLASNATLNNTIGFYNTSFGQSTDQ
VYGLFICRGDLSSTTVCQNCVTFATKDIVQRCPIGIASIVYYDACILRYSNVNFFSKVDQIPGFSLLNTQNITTEPQ
RFNNLVGATANDLAARAASAPPGAKKFAVNKTSFDAFQNIYSLAQCTPDLSSSDCNRCLSAAIARLPICCSSKIGGR
VLFPSCYIHYEISEFYNAAESPPPPPVVLPSPPPPRSVTIPEGKVSLHDLSSFIVTIYRMHSSKFKFIS

>Potri.004G196400
MAEVINMPVDSLDRRGGSGGGGGGRDRKDKPNTDDQSSPPPPPQPQRRRERDSRERREDFDRPLNRRGGDYHDRNRS
PPPPQREREKEYKRRSSTSPPPPLPYRDRRHSPPPRRSPPYKRSRREDGGFDARRGSPRGGFGGGDRRFGYDYGGGY
EREMGGRPGYGEERSHGRYMGRGGGYQGGPSDWESGRGGHGDASNTVSAQREGLMSYKQFIQELEDDILPSEAERRY
QEYKSEYISTQKRVFFEAHKDEEWLKDKYHPTNLVAVIERRNELARKVAKDFLLDLQSGTLDLGPGVNASSSNKSGQ
ASDLNSDDEAEAGGKRRRHGRPPAKDADLLSAAPKAHPVSSEPKRIQVDIEQAQALVCKLDSEKGVEDNILSGSDNE
KMSREKSHGSSTGPVIIIRGLTSVKGLEGVELLDTLITYLWRVHGLDYYGMIETNEARGLRHVRTEGKSSDSSNSGT
EWEKKLDLRWQERLRGQDPLEEMTAKEKIDAAAVEGLDPYVRKIRDEKYGWKYGCGAKGCTKLFHASEFVQKHLKLK
HPELVIDLTAKVREELYFQNYLNDPDAPGGTPVMQQSLPKDKPQRRKLGPENRLKDERGNRRERENRANGNERFDRS
ENPQSGDFQSNDGPDGGNCDETMFDTFGGQGIRVPPPFPSDIAPPPVLMPVPGAGPLGPFVPAPPEVAMRMFRDQGG
PPPFESGGRNARPGPQLGGPAPILLSPAFRQDPRRIRSYQDLDVPEDEVTVIDYRSL

>Potri.004G025200
MAVNVNDIGDATDRNQFNTNLGGLMNQLITRAASSSNLFAMGDTNGTAFTRIYGMVQCTPDISPSQCRICLSGCVSF
IPTCCNGKQGGNVLTPSCSMRFETYAFYTAPPSPPPPASSPSPPPPPATSLNPSGERKASSRTIVYISVPTSAFVVL
LFSLCYCYVHKKARKEYNAIQEGNVGDEITSVQSLQFQLGTIEAATNNFAEENKIGKGGFGDVYRGTLPNGQHIAVK
RLSKNSGQGAAEFKNEVVLVARLQHRNLVRLLGYCLEGEEKILIYEFVPNKSLDYFLFDPAKQGLLNWSSRYKIIGG
IARGLLYLHEDSRLRIIHRDLKASNVLLDGEMNPKIADFGMAKIFGGDQSQGNTSKIAGTFGYMPPEYAMHGQFSVK
SDVYSFGVLILEIISGKKNSSFYQSDNGLDLVSYAWKQWKNGAVLELMDSSFGDSYSRNEITRCVHIGLLCVQEDPN
DRPTLSTIVLMLTSFSVTLPLPREPAYFGQSRTVPKFPTTELESDRSTSKSKPLSVNDMSITELYPR

>Potri.004G025600

MNSLKYYVILLSLLTLAIITLAQEDANYLHHNCQNASTSAINSTYRVNLNLLLSSLASNATRNNTNGFYNTSFGQNT
DQVYGLFICRGDLSNTVCRNCVTFATEDIVHRCPIGIASIVYYDECILRYSNVNIFSKVDQSPSFSFSLLNTQNITT
EPQRFNNLVGAAANDLAARAASAPPGAKKFAVNKTSFNAFQNIYSLAQCTPDLSSSDCNRCLSAAIAGLPNCCSSKI
GGRVLFPSCYIHYEITEFYNATAVAAESPPPPPPPVVLPSPPPPRSVTIPEGILAM

>Potri.004G087100
MENPRLALLSSSSPKLVMGYPTSLKNPTTPKFSISTTRPSLPFSLRISKTAPHASIFSISALANSHGHRRTSKNGKL
GSEYFASISSSSGKQTASVGVNPQPVSPPPSQIGSPLFWVGVGVGLSAIFSWVATRVKNYAMQQAFKSLTEQMNTQN
NQFNPAFSARPPFPFSPPPASHPSTSPSPAASQPAITVDIPATKVEAAPTTDVGKEKETDFLEERKIKEETKKYAFV
DISPEETSLNTPFSSVEDDNETSSSKDVEFAKKVFQNGAAFKQGPGAAEGSQSTPGPFLSVEALEKMMEDPTMQKMV
YPYLPEEMRNPTTFKWMLQNPQYRQQLEDMLNNMGGSGKWDSQMMDSLKDFDLNSAEVKQQFDQIGLTPEEVISKIM
ANPDVAMAFQNPRVQQAIMECSQNPINITKYQNDKEVMDVFNKISELFPGMTG

>Potri.013G105900
MAEESSPPPPVLEQAPPSPAIEKDKEDLPPPAPVEEAESQAVQEKEEVPPQPAAAEQKESRSSSLAAMIEKEESFSP
PPQPLFSPEEKTEVTEKSVATKEEVAVVPDTGKEKKVPLTLVSFKEESNALADLSHIERRALEELKQLVQEALSSHQ
FSSAPPKKEEKQSVSVIQETSKTEAPVPDSDVGTDMKPSAENQESKVEKTPEKESQEVAKEEQKVASSSPEEVPIWG
IPLLKDDRSDVVLLKFLRARDFKVRDAFVMIKNTIQWRRDFKIDELVDEDLGDDLEKVVFMHGYDREGHPVCYNVYG
EFQNKELYQKTFSDEEKRLKFLRWRIQFLERSIRKLDFSPGGISTIFQVNDLKNSPGPGKRELRLATKQALLSLQDN
YPEFVAKQVFINVPWWYLAFYTVMSPFMTQRTKSKFVFAGPSNSAETLFKYISPEQVPIQYGGLCVDFCDCNPEFTI
ADPATDITVKPATKQTVEIIIYEKCILVWELRVVGWEVSYSAEFMPEAKDAYTIIITKPTKMSPTDEPVVSNSFKVG
ELGKILLTVDNPTSKKKKLLYRFKINPFSD

>Potri.013G054100
MEFPHGYYPQTHHHRRNDEEEERREHYPPPSFDQTPPPLFYRENEFAPAPRPYSHYYQESPQPPRPYFNETNYSPPP
PPTSIQETQVFHTSSFDQTPPPLFYGENEFAPPPRPYSHYYQESPQPPRPYFNETNYSPPPPPTSIQETQVFHTSHH
QGVDPSLDYPPAPTQVTHVSHEQTEARHSFRPHMPSFNHQHTHQPGAASGLDLYNKPSFKVYSKAQPEFHLTIRGGK
VILAPSNPSDEFQNWYKDEKYSTRVKDSEGCPAFALVNKATGQAMKHSIGEAHPVQLIPYNPDVLDESILWTESKDL
GDGFRAVRMVNNTHLNVDAFHGDKKSGGVHDGTSIVLWKWNKGDNQRWKIIPTRY

>Potri.011G029800
MGSKTIVSLLFHVIIISITLTGAEVCYNTGNFTANSTYAKNRDLVLRSLASNVTANGGFYNTTIGLGNDTVYGLVLC
MASPSAENCSSCVNYAIQTLMAGCPNQKEAISWGGNPLPCIVRYANRYFFGSLETSPTSAYYNEGILDATFRQFEQF
WSGLGETVKNASTGSSRLMPAVETADLPSTQKIYVFMQCTPDVSPSNCSVCLQESVDYYKSCCYGNQGGLVLKPNCV
FRWEIYTYYDLFPQVTSPPPSPSPSPSPSPSPSPSSPPFVISSPPPTNTTIRKGKENTASRTVIVTIVPTAIFLALV
ILILTVFHFRKPKQEVENFDEISIAKCLEFKFATIKLATNDFSDDNKLGQGGFGAVYKGILADGQAIAVKRLSSNSG
QGEVEFTNEVRLLAKLDHRNLVRLLGFCLEGTEKLLIYEFVPNSSLDQFIHDPNKRFILDWEKRYKIIEGIARGILY
LHQDSQLRIIHRDLKPSNILLDGKMNAKISDFGMAKLMKTDQTHDAASRIAGTFGYIAPEYARQRQFSVKSDVFSFG
VLVLEIVSGQKPSFRDGDDIEHLTSHAWRRWREGTALDLIDPILRNDSTAAMMTCIHIGLLCVQENVADRPTMASVV
LMLSNSSFTLQIPSKPAFFISRRTYRPASSSTSYTSRMTQSHLKTVPPSKNEISITELDPR

>Potri.011G029100
MGSKTIVSLLFHVIIVSITLTGAEVCYNTGNFTANSTYAKNRDLVLRSLASNVTNNGGFYNTTIGLGNDTVYGLVLC
MASPSAENCSRCVSSAIQTLTAGCPNQKEAISWGGNPLPCIVHYANRYFLGSLEQSPNSILYNVGILDATFRQFEQF
WSGLGETVKNASTGSSRLMPAVETADLPSNQKAYVFMQCTPDVSPSNCSVCLQQSVNDYKSCCYGHQGGIVQKPNCV
FRWDLYPIYDLFPQVTSPPPSPSPSPSPPSPSPSPPFVISSPPPTNTTIRKGKENTASRTVIVTIVPTAIFLALVIL
ILTIFCFRKPKQEVKNFDEISITKCWEFKFATIKLATNDFSDDNKLGQGGFGAVYKGILADGQAIAVKRLSSNSGQG
EVEFKNEVRLLAKLDHRNLVRLLGFCLEGTEKLLIYEFVPNSSLDQFIHDPNKRFILDWEKRYKIIEGIARGILYLH
QDSQLRIIHRDLKPSNILLDGKMNAKISDFGMAKLMKTDQTHDAASRIAGTFGYIAPEYARQRQFSVKSDVFSFGVL
VLEIVSGQKPSFRDGDDMEHLTSHAWRRWREGTALDLIDPILRNDSTAAMMRCIHIGLLCVQENVADRPTMASVLLM
LSNSSFTLQIPSKPAFFISRRTYQPASSLISYTSRMTQSQLKTVPPSKNEISITELDEGR

>Potri.011G143100
MAKPTRGRPTSRSGSGSGSSSRSRSYSGSDSRSSSRSRSVSRSRSRSRSLSSSSSSPSRSASSGSRSPPPRKRSPAE
GARRGRSPPPQSKKVSPPPRKASPIRESLVLHVDSLTRNVNEGHLREIFSNFGEVVHVELAMDRTVNLPKGFGYVEF
KTRADAEKALLYMDGAQIDGNVVRAKFTLPPRQKLSPPPKPIAAAPKRDAPKTDNASADAEKDGPKRQREPSPHRKP
LASPRRRSPVARRGGSPRRQLDSPPRRRADSPGRRRVESPYRRGETPRRRPASPARGRSPSSPRRYRSPLRASPR

RMRGSPVRRRSPPPRRRTPPRRARSPPRRSPLRRRSRSPIRRPARSRSRSLSPRRGRAPGARRGRSSSYSGSPSPRK
ATRKISRSRSPRRPLRGRSSSNSSSSSSPPRKP

>Potri.011G030100
MGSKTIVSLLFHVIIVSITLTDAQFCYTTGNFTANSTYAKNRDLVLRSLASNVTNKGGFYNTTIGLGNDTVYGLVLC
MASPSAGICSSCVNSAIQTLMAACPNQKEAISWGGNPLPCIVHYANRYFLGSLEPSPSSAFYNTGILDATFRQFDQF
WSGLGETVTKASTASSRLMPVAETADLSSTQKIYVFMQCTPDVSPSNCSVCLQQSVDYYKSCCYGHKGGGAQKPNCF
FRWDLYPFYDLFPQVTSPPPSPSPSPSPPFVISSPPPTNTTIRKGKENTASRTVIVTIVPTAIFLALVILILTVFHF
RKPKQEVENFDEISIAKCLEFKFATIKLATNDFSDDNKLGQGGFGAVYKGILADGQAIAVKRLSSNSGQGEVEFKNE
VRLLAKLDHRNLVRLLGFCLEGTEKLLIYEFVPNSSLDQFIHDPNKRFILDWEKRYKIIEGIARGILYLHQDSQLRI
IHRDLKPSNILLDGKMNAKISDFGMAKLMKTDQTHDAASRIAGTFGYIAPEYARQRQFSVKSDVFSFGVLVLEIVSG
QKPSFRDGDDIEHLTSHAWRRWREGTALDLIDPILRNDSTAAMMTCIHIGLLCVQENVADRPTMASVVLMLSNSSFT
LQIPSKPAFFISRRTYQPASSLTSYTSRMTQSHLKTVPPSKNEISITELDPR

>Potri.011G029200
MGSKTIVSLLFHVIIISITLTGAEVCYNTGNFTANSTYAKNRDLVLRSLASNVTANGGFYNTTIGLGNDTVYGLVFC
MASASAEICSSCVNSAIQTLMAACPNQKEAISWGGNPLPCIVRYENRYFFRSLEPSPTSALYNTGILDATSRQFDQF
WSGLGETVRNASTGSSRLMPAVETADLPSTQKIYVFMQCTPDVSPSNCSVCLQQSVDYYTSCCYGHQGGIVLKPNCV
FRWDLYPFYDLFPQVTSPPPSPSPSPPSPPFVISSPPPTNTTIRKGKENTASRTVIVTIVPTSIFLALVILILTIFR
FRKPKQEVKNFDENSSTKCWEFKFATIKLATNDFSDDNKLGQGGFGAVYKGILADGQAIAVKRLSSNSGQGAVEFRN
EVGLLAKLAHRNLVRLLGFCLEGTEKLLIYEFVPNSSLDQFIHDPNKRLVLDWEKRNKIIEGIARGIVYLHQDSQLW
IIHRDLKPGNILLDGNMNAKISDFGMAKLMKTDQTHDATSRIAGTFGYIAPEYAWKGQFSVKSDVFSFGVLVLEIVS
GQKPSFRNGDDMEHLTSHAWRRWREGTALDLIDPILRNDSTAAMMRCIHIGLLCVQENVADRPTMASVVQMLSNSSL
TLQTPFEPASSLSYTSTMEQSQLKIIPLSKNEISITELDPR

>Potri.006G018700
MVIKVVDLECKKCHKKIKKVLCGISQIQNQIYDKKENTVTITLVGCCPEKIKTKIYCKGGEAVKCIEIKPPPPSPPP
SPPPPPSPPPSPPPPPPSPPPSPPPPPCTCTCCEKCRRGPCCHHFCMPTVPPYCYVPCRRSECDIWGDGCCSCRSRG
YYVCRSLYVYEEYYYPPTCRIM

>Potri.006G018400
MVIKVVDLGCEKCHKKIKKVLCGIPREEIQNQIYDKKENTVTITVVCCSPEKIKKKIYCKGGEAVKCIEIKLPPPPP
PPSPPPPPPPPPPPPPSPPPSPPPSPPPSPCTCTCCEKCRRGPCCHHFCMPIVPPYFHVPCRWSECDLWGDGCCSCR
SRGYYVCRSAYVYEEYYYPPTCK

>Potri.005G160500
MAYPYYSPPPPSPPPPPPTSQVLCNPITTSPPPKHHRRPPPHPQLKPPPPPPKHHHRPPPHPQLKSSPPPPRSPSYQ
YDCRHPPLTPVKAPPCQDFAPSPSPSTTPYSKPPTPTSTAPTQPPSHRKPTLPKPSSPLPSYAPSPAPPYASSPPKQ
TTTSPTPMPSPYKPTPPKPSSPPSHIVPAPEPTPTGHTPMPSPYKPTPPKPSSPPSHTPGPVPPPESTPTRPAPVPS
PYKHTPPKPSYPPSHTPGPVPPPEPTPTGRAPVPSPYKHTPPKPSYPPSHTPGPVPPPEPTPTRPAPVPSPYKPTPP
KPSYPPSHTPGPVPPPEPTPTRPAPVPSPYKPTPPKPSYPPSHTPGPVPPPEPTPTRPAPVPSPYMPGPPQPLKPPS
YAPGSAPPPKLAWAPSPDKPTSPKPPHATPPSYHVSPPTPLNGPVSPPKLPPYATPPQYNIPPPPKPSSAPPVYAAP
PTFNSVPPSSNVVSPPPGGNHTTVIVVCVSLGGVFFLAFLLIGLICLAKKKKPVMVPVAPVCIEEHEVIQETITTG
QSEEETATVSMEDDVRIHGVLGVVGSGSSSNTSSPSGPSSPSESGRQPKG

>Potri.014G124800
MCSACENPCQPLPSTPPPPPPVSTCPPPPAVVSGSPPPPSMPNSGTIYYSPPPPSMPNSGTIYYSPPPPNSVPTYAY
SPPPPPADVAGFYPPPRYGNYPAPPPPNPILPYFPFYYYNPPPSSSPNSVLLKMNPIVFFIFLLLSFFPLLLLN

>Potri.T090800
MNSLKFYIILLSLLALAILTLAQDDANYLYHNCQNATTSTINSTYRVNLNLLLSSLASNATRNNTNGFYNTSFGQNT
DQVYGLFICRGDLSNTVCRNCVTFATEDIVQRCPIGITSIVYYDACILRYSNVNFFSKVDQNPGFYMWNLQNITTEP
QRFNNLVGATVNDLAARAASAPPGAKKFAVNETSFDAFQKIYSLAQCTPDLSSSDCNRCLSAAIAGLPNCCSNKIGG
RVLFPSCYIRYESTEFYDAAAVAAESPPPPPPVALPSPPPPRSATIPEGKVSLHYLLFFSISSIATLTKSLLYIEC
TRRNLNLSLKFFG
