# Supplementary material for: Bioinformatic Identification and Analysis of Hydroxyproline-Rich Glycoproteins in Populus trichocarpa
Source: BMC Plant Biol. 2016 Oct 21;16:229. doi: 10.1186/s12870-016-0912-3 (PMC5073881; doi:10.1186/s12870-016-0912-3)
Supplement: Additional file 4: Figure S4. — Protein sequences encoded by the predicted PRP genes in Populus trichocarpa. The colored sequences at the N terminus indicate the predicted signal peptides (green). PPV (pink) repeats typical of PRPs are indicated. Repetitive motifs PPLP (teal) and PELPK (dark yellow) are also indicated. Additionally, EXT SP3 (blue) repeats, YXY (dark red) and sequences typical of AGPs, specifically AP, PA, SP, TP, VP, and GP repeats, are indicated (yellow) if present. Note that green font indicates a predicted signal peptide using the sensitive mode from the SignalP website. Internal green highlights indicate the presence of a predicted signal peptide only if amino acids at the N terminus are discarded. (PDF 47 kb) [file 12870_2016_912_MOESM4_ESM.pdf]

Supplemental Figure 4

>Potri.004G168600-PtPRP1
MRILPVFRGAVLCFYVSLLFAATFCYAGDKTVEVVGTGECADCAESNIKTVHAFSGLRVTIDCKPENGEFKTRGFGE
LDEEGKFKVSLPSEVVKDGKLKEECYAQLHSASAAPCPSHDGLESSKIVLKSKTDEKHTFGLAGKLKFSPVTCTSAF
LWPHFKYPPLPKLPPLPKWKLPPLKDFHHPYLFPPKVFPPFPPKVFPPLPPKVFPPKVFPPKPFPPPLPVYKKPLPP
PVPIYKPEPKPPVFKPPPVPIYKKPLPPPVPIYKPEPKPPVFKPPPVPIYKPKPPVFKPPPIPIYKKPLPPPVPIYK
PEPKPPVFKPPPVPIYKKPLPPPVPIYKPEPKPPVFKPPPVPIYKPKPPVFKPPPVPIYKKPLPPPVPIYKPEPKPP
VFKPPPVPIYKPKPPVFKPPPVPVYKPKPKPPIYKPLPPPVPISKPLPPIPKIPPFYKKPLPPLPKLPPHPKIPPIY
KPKPPVFKPPPVPIYKPEPKPPIYKPLPPPVPIYKPLPPIPKIPPFYKKPCPPLPKLPPLPKLPPHPKIPPKYLNHP
KFGKWPPLPPYSPIH

>Potri.016G015500-PtPRP2
MAKFALANLLILLVNLGTLLTSLACPYCPYPAPPSKPPQYPPKIPPVHPPPKVKPPPKYPPKIPPVHPPPKVKPPPK
YPPKIPPVHPPPKVKPPSKYPPKIPPVHPPPKVKPPPKCPPKIPPVHPPPTVKPPHDPKPPKPHPPKPPVVPKPPIV
HPPFKPKPPIVHPPYTPKPPIVHPPFKPKPPIVHPPFKPKPPIVHPPFIPKPPYVPKPPVLPPTPPALPPPKPPVIP
PIKPPTPPALPPPTLPPPKPPVTPPIKPPTPPTLPPPKPPVTPPIMPPTPPTLPPPKPPVTPPIMPPTPPTPPTLPP
PKPPVTPPIMPPAPPTLPPPKPPVTPPIKPPTPPIVNPPPPETPCPPPPPPPPKQETCSIDTLKLGACVDVLGGLVH
IGIGSSAKDACCPVLQGLLDLDAAICLCTTIKAKLLNISIIIPIALEVLVDCGKTPPEGFKCPA

>Potri.014G126200-PtPRP3
MALTHSSSATFLLLLSLSVIASAGGYGYDQKPDTPKPNYTYNPKPQPDTSKPKYSYDPKPQPDVVKPDLHKPYYDSN
PKPTLPKPKFTEPKPDNGYDSKPYLGQPTIPKQDTAKPNYGYNPKPEVPKPKLTMPKPNHGNDPKPKLIVPKPDTAK
PNYGYNPKPEVPKPNYEYVPKPNLLKPKMTVPKPDHGYDPKEKVYEQPKSTTPKPEIITPHDGYAQKPNLPEPKLYI
PKPGNDKLDYDYEYSPIGIEGFVLCKQGSNYTPIEGAVIRIACTAVDQYGYKKVPFSCLTEATNAKGYYFKTLPALK
LTECKAYLESSPLKTCNVPTDMNYGITGAPLSAYHILHDKKIKLYSMRTFFYTSTTPTSTPAGY

>Potri.014G126500-PtPRP4
MALTHSSSATFLLLLSLSVIASAGGYGYDQKPDTTKPNYTYNPKPQPDTSKPKYSYDPKPQPDVVKPDLHKPYYDSN
PKPTLPKPKFTEPKPDNGYDSKPYLGQPTIPKPDTAKPNYGYNPKPEVPKPKLTMPKPNYGNDPKPKLIVPKPDTAK
PNYGYNPKPDVPKPNYEHVPKPKMTVPKPDHGYVDPKEKVYEQPKSTTPKPEIITPHDGYAQKPNLPEPKLYIPKPS
NDKLDYEYSPLGIEGFVLCKQGSNYTPIEGAVIRIACTAVDQYGYKKVPFSCLTEATNAKGYYFKTLPALKLTECKA
YLESSPLKTCNVPTDMNYGITGAPLSAYHILHDKKIKLYSMRTFFYTSTTPTSTPAGY

>Potri.018G126000-PtPRP5
MDSTKISALLFICMIFISSATSILGCHSCGNPKNKHPKTPKGSITLPPIVKPPVTLPPIVKPPVTLPPLPVPPIVKP
PVTLPPLPVPPIVKPPVTLPPLPVPPIVKPPVTLPPLPVPPIVKPPVTLPPLPVPPIVKPPVTLPPLPVPPIVKPPV
TLPPLPIPPVLPVPPVTLPPLPIPPVLPVPPVTLPPLPIPPVLPVPPVTVPPVITNPPKGKPCPPPPSSKDTCPIDT
LKLGNCVDLLGGLVHVGLGDPVVNQCCPVLKGLVELEAAVCLCTTLKIKALNLNIYVPLALQLLVTCGKTPPPGYTC
SL

>Potri.009G129900-PtPRP6
MRILPVFRGALLCFYVSLVFAAAFCYADDSTAEVVGIGECADCAQSNIKTVHAFSGLKVTIDCKPENGEFKTRGVGE
LDEEGKFKVSLQNDVVKDGKLKEECYAQLHSASAAPCPAHNGLESSKIVFKSKTDEKHTFGLAGKLKFSPVTCTSAF
LWPHPPITKPLPLPTWKLPPSKNFHHPYLFPPKPKPKPPIFKPPPVPIYKPKPKPPIFKPLPPPIPIYKPLPPPVPI
YKPLPPIPKIPPFHKKPCPLPKLPPYPKIPPKYFHHPKFGKWPPLPPHSPIH

>Potri.003G111300-PtPRP7
MGSKFAAMLFIFMIFMAISLPPIYACTPCTQPHPPSYPHPPTRPIVPHPKPPTTKHPPHHGGHSPSKKPPLPPVVLP
PIIINPPPVITPPVIAPPITNPPVITPPPSSSYPPYPPGSGGPPFGGGGGGGGGGGGSIPGVNPPPTTQPTCPINA
LKLGACVDVLGGLVHVGLGNPVENVCCPVLKGLLELEAAICLCTSIRLKLLNLTIFIPLALQVLITCGQTPPPGFVC
PPL

>Potri.006G008300-PtPRP8
MAKFAVANLLILLLNLGALLTSLACPTCPYTPHPKPPKRPPIKPPKPPVTPPIKPPKPPIKPPKPPVTPPIKPPKPP
IKPPKPPVTPPIKPPKPPVTPPVIPIPPTLPPPKPPVTPPVIPTPPILPPPEPPVIPTPPIVKPPPTPPKQETCPID

TLKLGACVDVLGGLVHIGIGSSAKDECCPLLEGLVDLDAAVCLCTVIKAKLLNINLILPIALELLVDCGKNPPEGFK
CPS

>Potri.T162800-PtPRP9
MDKFALANLLILLVNSGTLLTSLAYPDCTYPVPPSKLPNYPPKLPPVHIPPPPPPLSPLKPLVTPSIKPPPPRRPPV
TPPIKPPPPSLPPLNSSVIPKPPIIKPPPPETPCPPHLSPPELCPPPPPPPKQETCPIDTLKLGACVDVLGGLVHIR
TGSSVNDECCPVLEGFIDLDVASCLDIVIKAKLLNINLIIPIALKVLAECGKTPPPGFKCPA

>Potri.006G008600-PtPRP10
MAKFALANLLILLVNSGTLLTSLAYPDCTYPLPPSKPPNYPPKLPPVHIPPPPPPPPPLRPPVTPPIKPPPPRRPPV
TPPIKPPPPSLPPLNPPVIPKPPIVKPPPPETPCPPHPSLLELCPPPPPKQETCPINTLKLGACVDVLGGLVHIRTG
SSAKDECCPVLEGLVDFDVASCLCTVIKAKLLNINLIIPIALEVLADCGKTPPPGFKCPA

>*Potri.002G201800*-PtPRP34
MALTRFFFAASILLLSSLVITSANDYSYDSRTDTVKPGYHPKSDANIYDNTPKPDLPKPTLTIPKSDNEKPNYGYDS
IPEAPLPIGIEGLVLCKSGSNYIPIKGALVRIACMAVDQNGYETTPFSCLTGATDANGYYYKTLPAFGLGDLKVTEC
KAYLESSPLETCKIPTDVNNGMSGALLSSYHILSKNIKLYSMRTFFYTSETTPTPAGGY

>*Potri.017G145800*-PtPRP35
MALTHFFCAASILLLSLLVIASAADYGYEPKPDIVKPETSYVPAPKPKPTYDTPNSGNDQPKASYPGYGYGPYGPKS
DLPKPKPDFKYNPKPNVDLPKVTVPKIPNHGYHYIPMPHHLPKPKLSHGKPGYEPESLLPICVEGLILCKSGSNYIP
VEGAKVRILCTGVDQNGYEATHFSCLTDAADAHGYYFKTLFPFGGLGHNLKLKECKAYLENSPLETCKIPTDVNNGI
NGALLSSYHVLSNKNIKLYSMRTFFYTSETTSTSTTPPGGY

>*Potri.001G060500*-PtPRP38
MSAPMAPIIILVFLATLFALPLSLIARPATNPTSRISVVGVVYCDTCSTNTFSRHSYFLPGADVHIQCIFNANSPKT
REKIEFSVNRTTDRYGIYKLEVPEVDGVDCEEGFAIESVCQASLIGSSSRVCNVPGLRISTNEISVKSKQNNLCIYS
LNALSYRPSKKNITLCGSHKEELPNSLNSSKFFLPYFPPYGFPWPPLPNLPPLPNFPPLPFPPLPPFPSLPFPPLPS
LPFPPLPPIPFLPFPHLPFPSPPSLPFPFPPLPPLPPFPPAPSLFHPPPPPAFNLGDPRTWIPNIPSLAPPPPPAFN
LRDPKTWIPYIPPSPPSSPQNQNP

>*Potri.003G167100*-PtPRP40
MAPIIIHALLATLFALPLSLIAEPANNPTSRIIIVGVVYCDTCSTNTFSRYSCFLPGADVHIQCTFQAISPKTTEKI
QFSANRTTDRYGIYKVTVPEVDGVDCAVGSTIELVCQASLIRSSYPACNVPGLKMSTDVISVKSKQNNLCIYSMNAL
SYRPSKKNATLCGKHKEELQISFNSSKFFLPYSPPYGFPWHTLPNMSPSPFPPLPTPSQPFPPLPSPRPPSLPFPFP
PLSPTPSLFHPTPPPPPVAFNLGNPRTWIPNTPSLAPPPPPEFNLRDPRTWIPYIPPSPPNNPQNQNP

>*Potri.007G114400*-PtPRP44
MANHIFPLFISPLIVIMSMSLIISQTILVEARQLLEVTLPELPKPEFPELPKPELPKLPEFPIPELPKFEIPKLPEL
PKPEFPELPKPEFPKLPEFPKPELPKFEIPKLPELPPFPHFPDLTKPTLPTIPSHSTTMLPYPASSSRVTPKLKVAK
LQHHKGFHIIVVESRGPSSLDGMPDFQFETTPDGLPLIDADTTLPMVPELPKPELPEMPPLPKVELPTIPKPELPEL
PKPEVPKLPELPSFPHFPELPKTTLPTIPALPKDIKPPQSTTSP

>*Potri.013G111600*-PtPRP46
MLGNGGKATFGTAGIVGKLGSGGRVGLGRDDWVMGKVGKLGCGRVGMVGKGGNVGFGKFVTAGRGGNCRRWRAAKPP
LMLKNDKAKKKEYKNTHLTMASFKCFILTLFIALAISNINVAQAARRLLQLPPLPSLPNLSKPTLPPLPTIPSLPQP
TLPTLPTTQPSLPKPTLPPLPSLPTMPSVPKVTLPPLPSMLPLPTIPTAIPSIPFLTPPPGN

>Potri.006G065500-PtPRP11
MDSTKISAFLFLCMIFISSAAPTLDCGSCGKHPKNKHPKTPKAPITLPPLPVPPIVKPPVTLPPLPVPPIVKPPVTL
PPVTLPPVTVPPITVPPVTTKPPKGKPCPPPPSPKDTCPIDTLKLGACVDLLGGLVHIGLGDPVVNQCCPVLTGLVE
LEAAVCLCTTLKIKALNLNIYVPLALQLLVTCGKTPPPGYTCSL

>Potri.001G350600-PtPRP12
MAFKAVCLMVVAFVLVTAKASYMNEDFKEKAVFSKSVVPASTPAPPEVKSPTPAPPVVTPSTPLYKPPTPAPPVKTP
PPAPPVNPPTPVKPPTTPAPPVYKPPSPAPPVNPPTPVKPPTGPMPPPVRTRSDCTPLCGQRCKLHSRKRLCVRACM
TCCDRCKCVPPGTYGNREKCGKCYTDMTTRRNKPKCP

>Potri.T162900-PtPRP13
MAKFAVANLVILLLSLGALLTSIACPSCPSPPHPKPPVKPPKVKPPFHPKPPIVKPPKPPVTPKPPVVKPPKPETPC
PPPPVLPTPPIVKPPPTPPIQETCPIDTLKLGACVDVLGGLIHIGIGSSAKDECCPLLEGLVDLDAAVCLCTVIKAK
LLNINLILPIALELLVDCGKTPPEGFKCPS

>Potri.010G072200-PtPRP14
MQITSLLVLFVGVVVLATPSFADYYKPPKYEPKPPYFKPPKVEKPFPEHKPPVYKPPKIEKPPVYKPPPVYKPPIEK
PPVYKPPPVYKPPIEKPPVYKPPPSTSHHQSTSHQLRSHQSTSHQLRSHQSTSLQRLRSHHPFTSHCHLMVTIRDTL
QLKMRNISSQKTELNFLYIDMQEYY

>Potri.006G008500-PtPRP15
MAKFAVANLVILLLSLGALLTSIACPSCPSPPHPKPPVKPPKVKPPPIVKPPKPPVTPKPPVVKPPKPEKPCPPPPV
IPTPPIVKPPPTPPKQETCPIDTLKLGACVDVLGGLIHIGIGSSAKDECCPLLEGLVDLDAAVCLCTVIKAKLLNIN
LILPIALELLVDCGKTPPEGFKCPS

>Potri.007G113900-PtPRP16
MASFNCFVLALLIAFSFPGGEAARNLLQLPPLPAVPNLPKPTLPPMPSIPTLAQPPLPTLPTTQPSLPKPTLPPLPS
LPTTPSLPKVTLPPLPSMPSNIPTIPIPTTVPTIPIPTTIPSIPFLSPPPAGN

>Potri.007G114100-PtPRP17
MASLNCFILVLFIALSFSAGEAARNLLQLPNLPKPTLPPLPSIPTLPQPTLPTLPTTQPSLPKPALPPLPSLPVLPT
MPAVPTVTLPPLPSMPSIPTIPIPTAIPSIPFFSPPPATTKP

>Potri.007G113700-PtPRP18
MASCKSFILALFIALTFSSMNVSIAARHLLQLPPLPKIPMPPLPSIPNLPQPTLPTLPTTQPSLPKPTLPPLPSIPT
IPTFPTVPKVTLPPLPSMPSIPTIPTIPSIPFLSPPPATTSP

>Potri.017G047400-PtPRP19
MASRKSFILAFFIALAFSSMTVSVAARHLLQLPTLPPLPSIPNLPQPTLPTLPTTQPSLPKPTLPPLPSIPTIPTIP
TVPKVTFPPLPSMPSIPTIPTIPSIPFLSPPPATSP

>Potri.019G082600-PtPRP20
MAYFKCFIFALSIALAASGINVALAARHLLQLPPLPSVPNMPKPTLPPLPTIPTLPTTQPSLPKPTLPPLPSLPTMP
SVPKVNLPPLPSMPSLPTIPTTIPSFPFLSPPPGN

>Potri.017G047200-PtPRP21
MASFNCFILALFILLSLSGGQAARHLLQLPNLPKPTLPPLPSIPALPQPTLPSLPTSQPSMPKPTMPPLPILPKIPK
ATLPPLPSMPTLPAVPKVTLPPLSSMPSIPTIPIPTAIPSIPFLSPPPATTKP

>Potri.017G045800-PtPRP22
MASFSCFILALFIALSISGGEAARQLLQLPPLPAVPNLPKPTLPPMPSIPTLPQPTLPTAQPSLPKPTLPPLPSLPT
MPSLPKVTLPPLPSMPSMPTIPIPTTIPSIPFLSPPPGN

>Potri.017G046700-PtPRP23
MASFNSFILALFIALSISGGEAARQLLQLPPLPAIPNLPKPTLPPMPSIPTLPQPTLPTAQPSLPKPTLPPLPSLPT
MLSLPKVTLPPLPSMPSMPTIPIPTTIPSIPFLSPPPGN

>Potri.017G046400-PtPRP24
MASFNSFILALFIALSISGGEATRQLLQLPPLPAVPNLPKPTLPPMPSIPTLPQPTLPTAQPSLPKPTLPPLPSLPT
MPSLPKVTLPPLPSMPSMPTIPIPTTIPSIPFLSPPPGN

>Potri.017G045900-PtPRP25
MASFSCFILALFIALSISGGEAARQLLQLPPLPLVPNLPKPTLPPMPYIPTLPQPTLPTAQPSLPKPTLPPLPSLPT
MPSLPKVTLPPLPSMPSIPTIPIPTTIPSIPFLSPPPGN

>Potri.017G047000-PtPRP26

MASFSCFILALFIALSISGGEAARQLLQLPPLPSVPNFPKPTLPPMPSIPTLPQPTLPTAQPSLPKPTLPPLPSLPT
MPSLPKVTLPPLPSMPSMPTIPIPTAIPSIPFLSPPPGN

>Potri.017G047100-PtPRP27
MAAFKCLILSLFIALSFLGGEAARNLLQLPNLPKPALPPLPSIPSLPQPTLPTLPTTQQPSLPKPSLPPLPSMPTLP
TVVPKATLPPLPSMPTLPTVVPKLSLPPLPSMPSIPNIPLPTTIPSIPFLSPPPAGN

>Potri.017G045600-PtPRP28
MASFSCFILALFIALSISGGEAARQLLQLPPLPAVPNLPKPTLPPMPSIPTLPQPTLPTAQPSLPKPTLPPLPSLPI
MPSTLPVVTLPPLPSMPSMPTIPIPTTIPTIPIPTTIPSIPFLSPPPGN

>Potri.017G046100-PtPRP29
MASFSYFILALFIAQSISGGEAARQLLQLPPLPAVPNLPKPTLPPMPSIPKLPQPTLLTAQPSLPKPTLPPLPSLPT
MPSLPKVTLPPLPSMPSMPTIPIPTTIPSIPFLSPPPGN

>Potri.T178800-PtPRP30
MAAFKCLILSLFIALSFLGGEAARNLLQLPNLPKPALPPLPSIPSLPQPTLPALPTTQPSLPNPNLPPLPSLPTMPT
MPAVPKVSLPPLPSMPTLPTVVPKASLPPLPSMPSIPNIPLPTTIPSIPFLSPPPAGN

>Potri.007G114200-PtPRP31
MASFNCFILALLIAFSFPGGEAARNLLQLPPLPSVPNLPKPTLPPMPSMPTLPQPPLPTLPTTQPSLPKPTLPPLPS
LPTMPSLPKVTLPPLPSMPSNIPTIPIPTTIPSIPFLSPPPAGN

>*Potri.017G045000*-PtPRP37
MAGFSCFLILAIFMALSVSGGEAARHLLQFPPLPSVPNLPKPTLPSMPTLPQLTLPTSPKSQLSLPKPTLPPLPSLP
TMPSLPKVALPPLPTMPSIPFLSPPPGN

>*Potri.002G201900*-PtPRP39
MAPTSSYFAFFMSLSMVAAIASATDGGYGSHPNPNLVKPKLNKEKPLSTMIGVQGLVYCRSGPKRFPLEGAVIRITC
LANDVYGYEAAPFSFLSEATDAKGYFFATLSPYEMQDNLKIKECKAFLELSPLETCKIPTDEKQGISGALLASYHYL
SDKKMKLFTVGPFVYTSAPNSGSNY

>*Potri.017G044800*-PtPRP41
MSTTMANHNFPLFILRFFPLFVISSLSLMNSQTILVEARQLLEVTLPELPKPELPKLPPLPEFPKPELPELPEFEIP
KLPELPPFLHFPELPKPALPTIPRGINPSHSTTSP

>*Potri.017G044900*-PtPRP42
MAYRHFPSSILPLMVISMSLMNSQTILVEARQLLEAPLPELPKPELPKPELPELPKPEFPELPPKPELPKFEVPQLP
ELPTFPHLPELPKPTLPTIPKDINPSHSTASP

>*Potri.018G146200*-PtPRP43
MASLRFLTFISPLLLITLSLVDNTSRIEARRILETTLPKVPELPKPELPELPQLPKVELPPLPKPEFPELQKPEVPK
LPELPPFPHLPELPKSTLPTIPALPKDIKPPQSTTSP

>*Potri.007G114700*-PtPRP45
MANHIFPLFISPLIVIMSMSLIMSQTILVEARQLLEVTLPELPKPEFPELPKPELPKLPEFPIPELPKFEIPKLPEL
PPFPHFTDLTKPTLPTIPKDINPSHSTTSP

>*Potri.017G046800*-PtPRP47
MLPQFFFLLQSHTVLYLLISCSYTLFQALRLSIKMASLNCFILALFIAVSLSRGEAARHLLQLPPLPSVPSLPKPTL
PPLPSIPSLPQPTLPTLPTTQQPSLPKPSLPPLPSMPTLPTVVPKATLPPLPSMPTLPTVVPKLSLPPLPSMPSIPN
IPLPTTIPSIPFLSPPPAGN

>*Potri.017G045700*-PtPRP48
MARFNCFILALFIALSFSDGEVARQLLQLPALPSLPNLPKPTLPPLPSIPTLPQPTLPTLPATQPSLPKPPLPPLPI
LPTMPAIPKVTLLGFRIKND

>*Potri.017G046500*-PtPRP49
MARFNCFILALFIALSFSDGEAAHQLLQLPPLPSLPNLPKPTLPPLPSIPTLPQPTLPTLPATQPSLPKPPLPPLPI
LPTMPAIPKVTLLGFRIKND

>*Potri.004G114300*-PtPRP32I
MPWFFIIFLLGFTFNNPSEASHGKKLPSAVVVGTVYCDTCFQEYFSRNSHFISGAHVAVECKDEKSRPSFREEAKTD
EHGEFKVHLPFSVSKHVKKIKRCSVELLSSSEPYCAVASTATSSSLRLKSRKEGTHIFSAGFFTFKPEKEPFLCNQK
PSIENPREFSSKEASLPSFDNPTFPPPLQDPKTPVLPPLPPLPILPPLPQLPPLPPLPGLPFLPPIPANTENTKTTE
SLKSTTLPDEKAVHHPNQFGFPTPPLFPPNPFQPPPILPPIIQPPPLFPPILPPNPLQPPPVPSLPLPPVPSLPLPP
YHPFRCHQYLA

>*Potri.004G114400*-PtPRP33I
MPWFFIIFLLGFTFNNPSEASHGKKLPSAVVVGTVYCDTCFQEYFSRNSHFISGAHVAVECKDEKSRPSFREEAKTD
EHGEFKVHLPFSVSKHVKKIKRCSVELLSSSEPYCAVASTATSSSLRLKSRKEGTHIFSAGFFTFKPEKEPFLCNQK
PSIENPREFSSKEASPPSFDNPTFPPPLQDPKTPVLPPLPPLPILPPLPQLPPLPPLPGLPFLPPIPANTENTKTTE
SLKSTTLPDEKAVHHPNQFGFPTPPLFPPNPFQPPPILPPIIQPPPLFPPILPPNPLQPPPIQSLPLTPIPSLPLPP
IPSLPLPPIPSLPLPPIPSLPLPPPPFPLIPPPPFSLIPPLPLLPPLIPGIPPASSS

>*Potri.017G100600*-PtPRP36I
MPWFFIIFLLSCTFNNLSAEASHGKKLPSAVVVGTVFCDTCFQEAFSRNSHFISGASVAVECKDEESRPGFREEVKT
DEHGEFKVHLPFSVSKHVKKIKRCSVKLLSSSEPFCAVASSATSSSLHLKSRKQGTHIFSSGFFTFKPEKQPILCNQ
KPSTENSREFSSRKASLPSIDNPTFPPPLQDPTTPYLPPLNQNYLPPLPVLPKLPPLPQLPPLPPLPGLPLLPPIPG
NTKKTKTSESFESTTLPDQKAVHHPNQFSFPTPPLFPPNTFQLPPLFPPNPIQPPPSPLFPFPPIPGLTPPPPPPLF
PPNPIQPPPSPLFPFPPIPGLTPPPPPPLFPPNPIQPPPSPLFPFPPIPGLTPPPHPPPPPPPFSLIPPLPLLPPL
PPLPHLPPLIPGIPPASSSPQKTSP
